# ZnH_2_ as a Precursor to Catalytically Active Ru–ZnH Heterometallic Complexes

**DOI:** 10.1021/acs.inorgchem.4c05360

**Published:** 2025-02-19

**Authors:** Anne-Frédérique Pécharman, Ambre Carpentier, John P. Lowe, Stuart A. Macgregor, Mary F. Mahon, Michael K. Whittlesey

**Affiliations:** a Department of Chemistry, University of Bath, Bath BA2 7AY, United Kingdom; b Institute of Chemical Sciences, School of Engineering and Physical Sciences, Heriot-Watt University, Edinburgh EH14 4AS, United Kingdom; c EaStCHEM School of Chemistry, University of St Andrews, North Haugh, St Andrews KY16 9ST, United Kingdom

## Abstract

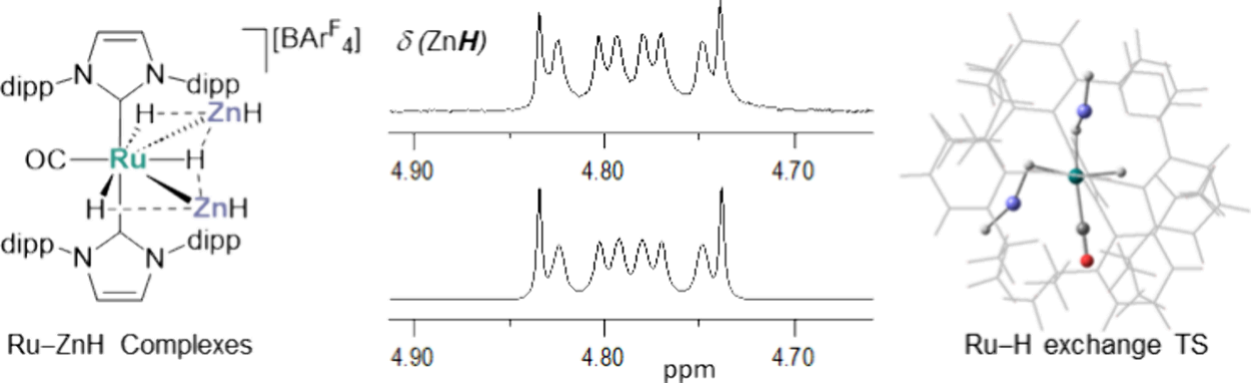

Reaction of [Ru(IPr)_2_(CO)H][BAr^F^_4_] (**1**; IPr = 1,3-bis(2,6-diisopropylphenyl)imidazol-2-ylidene; BAr^F^_4_ = B{3,5(CF_3_)_2_C_6_H_3_}_4_) with an excess of ZnH_2_ in THF gives the structurally characterized neutral Ru(ZnH) complex [Ru(IPr)_2_(CO)(ZnH)H_3_] (**6**) and Ru(ZnH)_2_ salt [Ru(IPr)_2_(CO)(ZnH)_2_H_3_][BAr^F^_4_] (**5**). Crystallographic and computational analyses show the presence of both bridging Ru–H–Zn hydrides and terminal Ru-hydrides in the two products. Calculations also identify a low-energy H/ZnH exchange pathway that rationalizes the experimentally observed (EXSY) fluxionality of the hydrides in **5**. At room temperature, this compound undergoes stoichiometric exchange with ZnMe_2_ to give [Ru(IPr)_2_(CO)(ZnMe)_2_H_3_][BAr^F^_4_] (**7**), and also proves to be catalytically active for the hydrogenation of 1-hexene and 5-hexene-2-one.

## Abstract 2

Zinc dihydride reacts with [Ru(IPr)_2_(CO)H][BAr^F^_4_] (IPr = 1,3-bis(2,6-diisopropylphenyl)imidazol-2-ylidene; BAr^F^_4_ = B{C_6_H_3_(3,5-CF_3_)_3_}_4_) to afford the heterometallic ZnH-containing compounds [Ru(IPr)_2_(CO)(ZnH)_2_H_3_][BAr^F^_4_] and [Ru(IPr)_2_(CO)(ZnH)H_3_]. Experiments and calculations have been used to probe the solution fluxionality and ZnH/ZnMe exchange of the former, which also proves to be catalytically active for alkene hydrogenation.

## Introduction

**Scheme 1:**
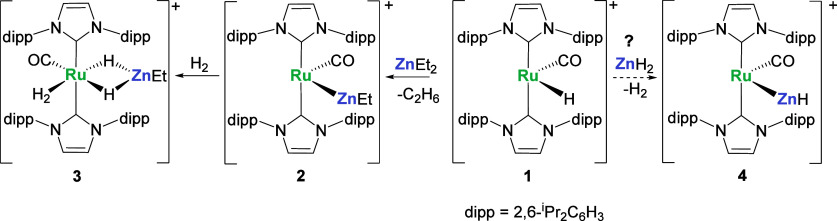
Synthesis and H_2_-addition Chemistry of the [BAr^F^_4_]^−^ (Ar^F^ = 3,5-(CF_3_)_2_C_6_H_3_) Salt of [Ru(IPr)_2_(CO)H]^+^ (**1**) with ZnEt_2_ and Potential for a ZnH Analogue **4**. [BAr^F^_4_]^−^ anions, as well as any agostic interactions, are omitted for clarity.

Alkane elimination is a tried and tested methodology for the synthesis of transition metal-main group metal (TM-MGM) heterometallic complexes. Most commonly, TM-hydride precursors are combined with MGM-alkyls,^1−6^ on the basis that there are a wide range of the latter that either are commercially available (ZnMe_2_, AlMe_3_ etc.) or readily synthesized as straightforwardly handled reagents. We employed this approach to prepare the Ru–Zn heterobimetallic salt [Ru(IPr)_2_(CO)(ZnEt)][BAr^F^_4_] (**2**; IPr = 1,3-bis(2,6-diisopropylphenyl)imidazol-2-ylidene; BAr^F^_4_ = B{3,5-(CF_3_)_2_C_6_H_3_}_4_) through combining [Ru(IPr)_2_(CO)H][BAr^F^_4_] (**1**) with ZnEt_2_ and ejecting ethane (Scheme 1).^7^ As a result of the coordinative unsaturation of both the Ru and Zn centers (so-called “dual unsaturation”), **2** readily activates H_2_ across the Ru–Zn bond, to give, in combination with the coordination of a second molecule of H_2_ at Ru, the dihydrogen dihydride salt **3**.

We were interested in the possibility of preparing a ZnH analogue of **2** (**4**, Scheme 1), which we envisaged might be accessible through loss of H_2_ following treatment of **1** with ZnH_2_.^8^ Although zinc dihydride has been known for over 75 years,^9,10^ its low thermal stability and poor solubility has restricted studies of its reaction chemistry.^11,12^ Moreover, most of the reported literature syntheses of ZnH_2_ either utilize, or afford as side products, MGM-hydride reagents (e.g., LiAlH_4_, [AlH_3_]_*x*_) that are likely to be at least as reactive toward TM-H compounds like **1**.^13,14^ Further obstacles include not only the difficulty of removing such MGM-hydrides, but also quantifying the amounts remaining in batches of ZnH_2_. Okuda and co-workers have recently described a synthetic route that appears to circumvent most of these issues. This involves the generation of ZnH_2_ by reacting ZnEt_2_ with AlH_3_·NEt_3_.^15^ The sole byproduct is AlEt_3_·NEt_3_, which remains in solution as ZnH_2_ precipitates out.

Intrigued by this approach, we now report the use of Okuda’s synthesis of ZnH_2_ for a study of its reactivity with **1**. Contrary to the anticipated loss of H_2_ and formation of **4**, we find instead that the di-ZnH salt [Ru(IPr)_2_(CO)(ZnH)_2_H_3_][BAr^F^_4_] (**5**) and the neutral mono-ZnH complex [Ru(IPr)_2_(CO)(ZnH)H_3_] (**6**) form via reactions that involve not only ZnH_2_, but also cationic [ZnH]^+^. Salt **5**, which adds to the group of TM(Zn)_2_ di- and poly hydride compounds that are increasingly prevalent,^16−18^ is active for the catalytic hydrogenation and isomerization of alkenes.

## Experimental Section

### General Comments

All manipulations were carried out at room temperature under argon using standard Schlenk, high vacuum and glovebox techniques using dry and degassed solvents. C_6_D_6_, C_6_D_5_CD_3_ and THF-*d*_8_ were vacuum transferred from potassium. NMR spectra were recorded at 298 K (unless otherwise stated) on Bruker Avance 400 and 500 MHz NMR spectrometers and referenced as follows: C_6_D_6_ (^1^H, δ 7.16; ^13^C, δ 128.0), C_6_D_5_CD_3_ (^1^H, δ 2.09), THF-*d*_8_ (^1^H, δ 3.58; ^13^C, δ 25.3). ^31^P{^1^H} NMR spectra are referenced to H_3_PO_4_ at δ = 0.0. IR spectra were recorded on a Bruker ALPHA ATR-IR spectrometer. Elemental analyses were performed by Elemental Microanalysis Ltd., Okehampton, Devon, U.K. [Ru(IPr)_2_(CO)H][BAr^F^_4_] (**1**),^19^ ZnH_2_^15^ and [Ru(PPh_3_)_3_(ZnMe)_2_H_3_][BAr^F^_4_]^16^ were prepared according to literature methods. ZnMe_2_ in toluene (Merck) was used as supplied. 1-Hexene and 5-hexene-2-one were freeze–pump–thaw degassed and stored over 3 Å molecular sieves.

#### [Ru(IPr)_2_(CO)(ZnH)_2_H_3_][BAr^F^_4_] (5)

A THF-*d*_8_ (0.5 mL) suspension of [Ru(IPr)_2_(CO)H][BAr^F^_4_] (40 mg, 0.023 mmol) and ZnH_2_ (4.5 mg, 0.067 mmol) was shaken in a J. Youngs resealable NMR tube for 6 h. The tube was evaporated to dryness, the residue redissolved in C_6_H_5_F and filtered. The filtrate was layered with hexane and then cooled to −35 °C to afford yellow crystals of **5** in 67% yield (29 mg). ^1^H NMR (500 MHz, THF-*d*_8_, 298 K): δ 7.79 (br m, 8H, BAr^F^_4_), 7.57 (br s, 4H, BAr^F^_4_), 7.56–7.49 (m, 8H, Ar + NC*H*=CHN), 7.46–7.42 (m, 8H, Ar + NCH = C*H*N), 4.79 (m, *AA’* part of *AA’BXX’* pattern, 2H, Zn*H*), 2.32 (sept, ^3^*J*_HH_ = 6.8 Hz, 8H, C*H*Me_2_), 1.24 (d, ^3^*J*_HH_ = 6.8 Hz, 24H, CH*Me*_2_), 1.03 (d, ^3^*J*_HH_ = 6.8 Hz, 24H, CH*Me*_2_), −8.94 (tt, ^2^*J*_HH_ = 15.6 Hz, ^3^*J*_HH_ = 5.0 Hz, *B* part of *AA’BXX’* pattern, 1H, Ru-*H*), −9.48 (m, *XX’* part of *AA’BXX’* pattern, 2H, Ru-*H*). ^13^C{^1^H} NMR (126 MHz, THF-*d*_8_, 298 K): δ 200.3 (s, Ru-*C*O), 179.3 (s, Ru-*C*_NHC_), 163.0 (1:1:1:1 quart, ^1^*J*_CB_ = 50 Hz, BAr^F^_4_) 146.7 (s, N*C*_ipso_), 138.4 (s, NC*C*_ipso_^i^Pr), 135.8 (s, BAr^F^_4_), 132.8 (s, N*C*H = CHN), 130.3 (br quart, ^2^*J*_CF_ = 32 Hz, BAr^F^_4_), 128.2 (s, *p*-*C*H_dipp_), 127.5 (s, *m*-*C*H_dipp_), 125.7 (quart, ^1^*J*_CF_ = 272 Hz, *C*F_3_), 118.4 (br s, BAr^F^_4_), 30.0 (s, *C*HMe_2_), 26.2 (s, CH*Me*_2_), 23.0 (s, CH*Me*_2_). ATR-IR (cm^–1^): 2030 (ν_CO_; calc. ν_CO_ 1991), 1872 (calc. ν_ZnH_ 1931, 1898), 1615 (calc. ν_RuH_ 1650), 1595 (calc. ν_transHRuH(asymm)_ 1602). Calcd for C_87_H_89_BF_24_N_4_ORuZn_2_: C, 54.84; H, 4.71; N, 2.94. Found: C, 54.75; H, 4.55; N, 2.72.

#### [Ru(IPr)_2_(CO)(ZnH)H_3_] (6)

Complex **6** was characterized spectroscopically (i) in reaction mixtures of **1** and ZnH_2_ at early times or (ii) upon reformation in THF solutions of **5**. The cleanest NMR spectra resulted from combining these two approaches, as follows for a typical preparation: A THF (0.5 mL) suspension of **1** (20 mg, 0.011 mmol) and ZnH_2_ (2 mg, 0.030 mmol) was shaken in a J. Youngs resealable NMR tube for 30 min and then evaporated to dryness. The residue was extracted with benzene (ca. 1–2 mL), filtered and evaporated to dryness. ^1^H NMR spectroscopy (THF-*d*_8_) showed the presence of **5** and **6**. Upon standing at room temperature for up to 72 h, there was depletion of **5** and an increase in the amount of **6**, albeit alongside [IPrH][BAr^F^_4_]. Selected ^1^H NMR (500 MHz, THF-*d*_8_, 298 K): δ 5.54 (m, 1H, Zn*H*), 2.56 (sept, ^3^*J*_HH_ = 6.8 Hz, 8H, C*H*Me_2_), 1.10 (d, ^3^*J*_HH_ = 7.0 Hz, 12H, CH*Me*_2_), 0.98 (d, ^3^*J*_HH_ = 6.6 Hz, 12H, CH*Me*_2_), 0.94 (d, ^3^*J*_HH_ = 7.0 Hz, 12H, CH*Me*_2_), 0.92 (d, ^3^*J*_HH_ = 6.9 Hz, 12H, CH*Me*_2_), −7.42 (dd, *J*_HH_ = 26.5 Hz, *J*_HH_ = 1.9 Hz, 1H, Ru-*H*), −7.61 (br d, *J*_HH_ ∼ 19 Hz, 1H, Ru-*H*), −7.81 (br s, 1H, Ru-*H*). Selected ^1^H NMR (400 MHz, THF-*d*_8_, 238 K): δ 5.54 (ddd, *J*_HH_ = 26.9 Hz, *J*_HH_ = 18.9 Hz, *J*_HH_ = 5.4 Hz, 1H, Zn*H*), −7.46 (dt, *J*_HH_ = 26.6 Hz, *J*_HH_ = 6.8 Hz, *J*_HH_ = 5.6 Hz, 1H, Ru-*H*), −7.62 (dd, *J*_HH_ = 19.4 Hz, ^2^*J*_HH_ = 4.9 Hz, 1H, Ru-*H*), −7.89 (t, *J*_HH_ = 5.6 Hz, 1H, Ru-*H*).

#### [Ru(IPr)_2_(CO)(ZnMe)_2_H_3_][BAr^F^_4_] (7)

ZnMe_2_ (48 μL of a 0.72 M toluene solution, 0.03 mmol) was added to a C_6_H_6_ (1 mL) solution of **5** (22 mg, 0.01 mmol) in a J. Youngs resealable NMR tube. Upon shaking for ca. 1 min, an insoluble colorless solid began to appear. The tube was shaken for 60 min and then evaporated to dryness. The residue was extracted into a minimum amount of C_6_H_5_F and filtered. The filtrate was layered with hexane and cooled to −35 °C to afford colorless crystals of **7** in 63% yield (14 mg). ^1^H NMR (400 MHz, THF-*d*_8_, 226 K): δ 7.86 (br s, 8H, BAr^F^_4_), 7.76–7.64 (m, 12H, Ar + BAr^F^_4_), 7.61–7.56 (m, 2H, Ar), 7.43 (t, ^3^*J*_HH_ = 8.0 Hz, 2H, Ar), 7.34 (d, ^3^*J*_HH_ = 7.6 Hz, 2H, NC*H*=CHN), 7.17 (d, ^3^*J*_HH_ = 7.6 Hz, 2H, NCH = C*H*N), 2.77 (sept, ^3^*J*_HH_ = 6.4 Hz, 2H, C*H*Me_2_), 2.45 (sept, ^3^*J*_HH_ = 6.4 Hz, 2H, C*H*Me_2_), 2.31 (sept, ^3^*J*_HH_ = 6.0 Hz, 2H, C*H*Me_2_), 1.44 (d, ^3^*J*_HH_ = 6.8 Hz, 6H, CH*Me*_2_), 1.41 (d, ^3^*J*_HH_ = 6.8 Hz, 6H, CH*Me*_2_), 1.14 (d, ^3^*J*_HH_ = 6.4 Hz, 6H, CH*Me*_2_),* 1.09 (d, ^3^*J*_HH_ = 6.4 Hz, 6H, CH*Me*_2_), 1.07–1.00 (m, 18H, CH*Me*_2_), 0.90 (d, ^3^*J*_HH_ = 6.4 Hz, 6H, CH*Me*_2_), −0.77 (s, 6H, Zn*Me*), −8.63 (t, ^2^*J*_HH_ = 4.8 Hz, 1H, Ru-*H*), −9.28 (d, ^2^*J*_HH_ = 4.8 Hz, 2H, Ru-*H*).*: the fourth C*H*Me_2_ resonance was obscured by the THF solvent at δ 1.73 as shown by ^1^H COSY. ^13^C{^1^H} NMR (126 MHz, THF-*d*_8_, 231 K): δ 198.4 (s, Ru-*C*O), 179.7 (s, Ru-*C*_NHC_), 163.1 (1:1:1:1 quart, ^1^*J*_CB_ = 49 Hz, BAr^F^_4_) 148.5 (s, NC*C*_ipso_^i^Pr), 146.6 (s, NC*C*_ipso_^i^Pr), 145.1 (s, NC*C*_ipso_^i^Pr), 144.4 (s, NC*C*_ipso_^i^Pr), 139.4 (s, N*C*_ipso_), 137.2 (s, N*C*_ipso_), 135.6 (s, BAr^F^_4_), 132.8 (s, *p*-*C*H_dipp_), 132.0 (s, *p*-*C*H_dipp_), 130.1 (br quart, ^2^*J*_CF_ = 32 Hz, BAr^F^_4_), 129.2 (s, *m*-*C*H_dipp_), 129.0 (s, *m*-*C*H_dipp_), 128.3 (s, *m*-*C*H_dipp_), 128.1 (s, *m*-*C*H_dipp_), 125.6 (quart, ^1^*J*_CF_ = 272 Hz, *C*F_3_), 118.5 (br s, BAr^F^_4_), 31.0 (s, *C*HMe_2_), 30.1 (s, *C*HMe_2_), 30.0 (s, *C*HMe_2_), 29.4 (s, *C*HMe_2_), 26.6 (s, CH*Me*_2_), 26.0 (s, CH*Me*_2_), 25.7 (s, CH*Me*_2_), 24.2 (s, CH*Me*_2_), 23.4 (s, CH*Me*_2_), 22.8 (s, CH*Me*_2_), 22.7 (s, CH*Me*_2_), 2.2 (s, Zn*Me*). ATR-IR (cm^–1^): 2039 (ν_CO_), 1615, 1595, 1577. Recrystallized samples of **7** always contained traces of [IPrH][BAr^F^_4_], precluding accurate CHN analysis.

### Catalytic Alkene Hydrogenation and Isomerization Reactions

A J. Young’s resealable NMR tube was charged with Ru compound (5 mg; equiv to 0.0026 mM for **5**) and 1,3,5-(MeO)_3_C_6_H_3_ (2 mg, internal standard) in either THF-*d*_8_ or C_6_D_6_ (0.45 mL). Alkene (10 equiv) was then added. Hydrogenation reactions were conducted by freeze–pump–thaw degassing the tube (3 cycles) and filling with 1 atm H_2_. The progress of reactions was monitored by ^1^H NMR spectroscopy by integration of the alkenic resonances against the internal standard. Organic products were identified by comparison to authentic samples and/or literature data.^20,21^

### X-ray Crystallography

Data for **5** and **6** were obtained at 150 K using an Agilent SuperNova instrument and a Cu–Kα source. SHELXT^22^ provided the solutions for both structures which were subsequently refined using SHELXL^23^ via the Olex-2 interface.^24^ Only noteworthy points follow. The asymmetric unit in the structure of **5** contains one cation and one [BAr_4_^F^]^−^ anion and a region of very diffuse solvent. The resolution of the former was sufficient to allow the five Ru and Zn-based hydrides to be located. These were refined with free *U*_iso_ values subject to symmetric pairs having similar M-H distances (i.e., related by rotation of approximately 180° from each other about an axis based on Ru1, C55 and O1). Disorder prevailed in the anion for the CF_3_ group based on C70, and the entire substituted phenyl moiety based on C80 which were each modeled as two components in 50:50 and 55:45 ratios, respectively. Solvent was treated using the masking algorithm available in Olex-2 and a consequent allowance made for one molecule of hexane per unit cell, in the formula as presented. The data for **5** were compromized by the presence of a small twin that accounted for approximately 5% of the diffraction. The minor component related to the major one by a 105° rotation about the reciprocal [0.22 0.97–0.03] direction. Integration as a twin and subsequent refinement using the reduced data from only the major component provided the optimal refinement of the model in this structure. Finalization of the twin-integrated data only served to degrade the statistics relative to a data reduction that entirely ignored the very minor component, probably due to scaling difficulty when the latter has a relatively small contribution to the diffraction.

In **6**, the asymmetric unit comprises one molecule of the mixed-metal complex and one molecule of THF. Disorder (75:25) was modeled for the zinc center and carbonyl ligand, with chemically equivalent bonds bearing distance similarity restraints across both components. ADP restraints were also included for the disordered atoms, to assist convergence. H1–H4 were located and refined freely (including their respective *U*_iso_ values) with full site-occupancy relative to the major component of the disordered zinc center. No effort was made to locate the 25% occupancy hydride fractions in the minor disordered component, as to do so would be futile with X-ray data.

### Computational Studies

DFT calculations were run with Gaussian 16 (Revision A.03).^25^ Ru and Zn centers were described with Stuttgart RECPs and associated basis sets^26^ with 6-31G** basis sets used for all other atoms.^27,28^ Optimizations employed the BP86^29,30^ functional. All stationary points were fully characterized via analytical frequency calculations as either minima (all positive eigenvalues) or transition states (one negative eigenvalue). Transition states were also characterized via IRC calculations and subsequent geometry optimizations to confirm the adjacent minima. Electronic energies were recomputed with the def2-TZVP^31,32^ basis set, corrected for the effects of THF or benzene solvent using the PCM approach^33^ and for dispersion (D3^34^ with Becke-Johnson damping^35^). These corrected electronic energies were combined with the thermochemical corrections from the BP86-optimized geometries to give the free energies quoted in the text.

Quantum theory of atoms in molecules (QTAIM)^36^ analyses used the AIMALL program^37^ and were performed on the experimental crystal structures with the heavy atoms fixed and the H atoms optimized. All BCP metrics are provided in atomic units and fuller listings including Laplacian (∇^2^ρ(r)) and total energy densities (H(r)) are provided in the Supporting Information (Figures S36–S37). Computed geometries are displayed with ChemCraft^38^ with all geometries supplied as a separate XYZ file.

## Results and Discussion

### Synthesis, Structure and Reactivity of [Ru(IPr)_2_(CO)(ZnH)_2_H_3_][BAr^F^_4_] (5)

**Scheme 2:**
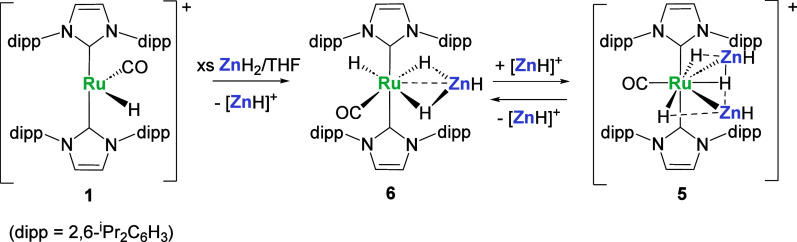
Pathway to [Ru(IPr)_2_(CO)(ZnH)_2_H_3_][BAr^F^_4_] (**5**) and [Ru(IPr)_2_(CO)(ZnH)H_3_] (**6**). [BAr^F^_4_]^−^ anions, as well as agostic interactions in **1**, are omitted for clarity

Addition of THF^39,40^ to a mixture of **1** and excess ZnH_2_ (2–3 equiv)^41^ gave an orange/yellow suspension which, upon shaking, converted to a gray/white suspension over a period of a few hours. ^1^H NMR spectra recorded after ca. 6–7 h showed one major species in solution, which was characterized as [Ru(IPr)_2_(CO)(ZnH)_2_H_3_][BAr^F^_4_] (**5**, Scheme 2) by NMR spectroscopy, elemental analysis and X-ray crystallography. This salt was isolated as a pale-yellow solid in 67% yield from cold C_6_H_5_F/hexane.

**Figure 1:**
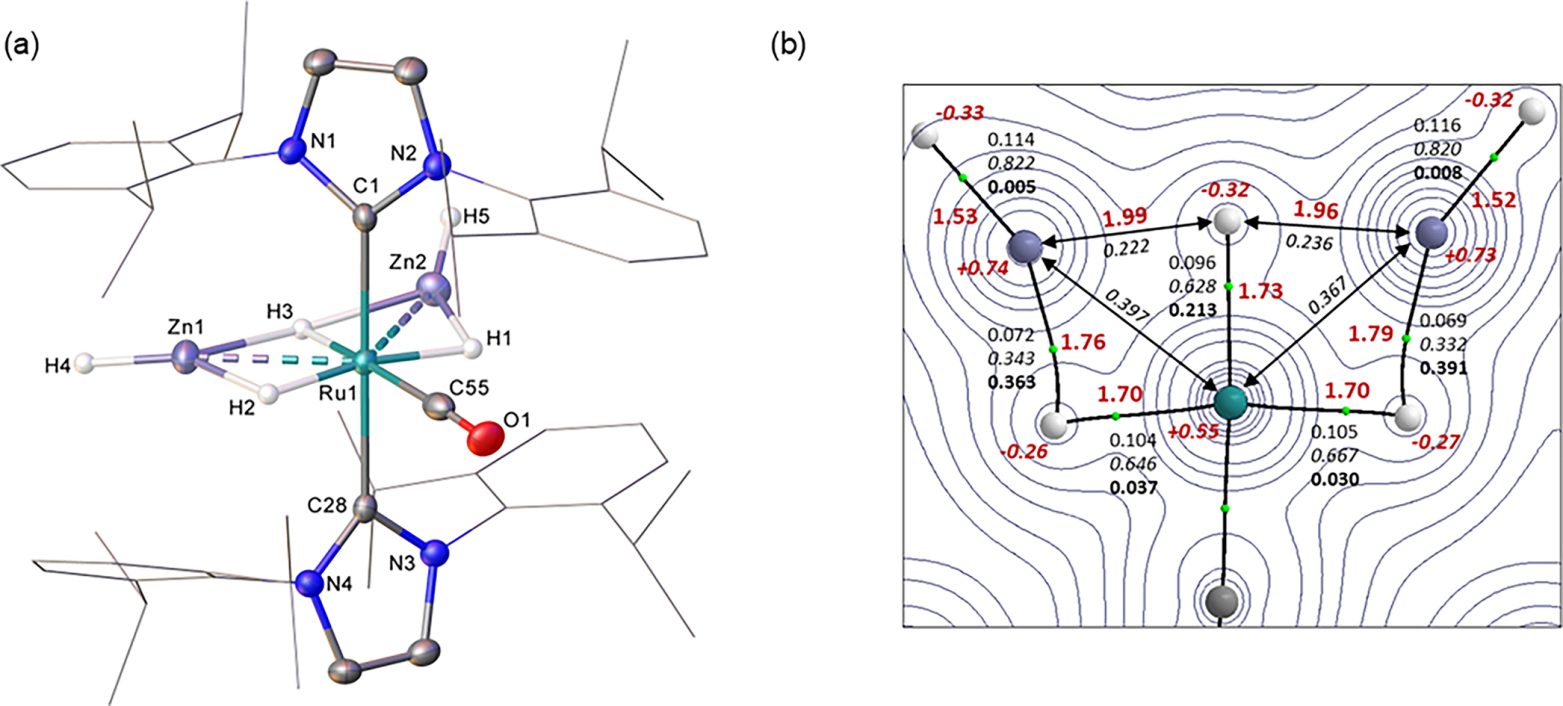
(a) Structure of the cation in **5**. Ellipsoids are displayed at 30% probability, the hydrogen atoms (hydrides excepted) are omitted for clarity and the NHC substituents displayed as wireframes for visual ease. (b) QTAIM molecular graph (X-ray structure with optimized H atom positions) showing density contours in the {Zn1Ru1Zn2} plane with computed Ru–H and Zn–H distances (Å, in red, plain text) and QTAIM atomic charges (in red, italics). BCPs (green spheres) have the associated ρ(r) (au) shown in plain text, delocalization indices in italics and, for bond paths to hydrogens, ellipticities in bold. Delocalization indices between selected atoms not linked by a bond path are also indicated.

The X-ray structure of the cation in **5** (**5**^**+**^, Figure 1A) displayed six equatorial ligands around Ru; one hydride trans to the CO ligand and two other hydrides (approximately trans to one another; H1–Ru1–H2, 169.1(19)°) each bridging across a Ru-ZnH group. As in the related bis-ZnMe salt, [Ru(PPh_3_)_3_(ZnMe)_2_H_3_][BAr^F^_4_],^16^ the Ru–H1 and Ru–H2 bond lengths (both 1.68(3) Å)) were shorter than the Zn1–H2 and Zn2–H1 distances (both 1.75(3) Å),^42^ suggestive of the hydrides being more closely associated with the ruthenium metal center. This pattern was confirmed by the calculated structure based on the experimental geometry with optimized H atom positions (distances in red, Figure 1B). A QTAIM analysis on this structure shows the same arrangement of bond paths around the {RuZn_2_H_3_} core as seen in [Ru(PPh_3_)_3_(ZnMe)_2_H_3_]^+^. For **5**^**+**^, the Ru1–H1 and Ru1–H2 bond critical points (BCPs) have higher electron densities and delocalization indices (average values: ρ(r) = 0.105 au; DI = 0.657) and much lower bond ellipticities (ε = 0.034) than the Zn1–H1 and Zn2–H2 BCPs (ρ(r) = 0.071 au; DI = 0.337; ε = 0.377). This suggests two Ru-hydride bonds are present that bridge to zinc. Although no Zn–H3 bond paths are seen, average Zn1···H3 and Zn2···H3 DIs of 0.229 indicate some Zn···H3 bridging interaction is present; this is also consistent with a weaker Ru–H3 bond (ρ(r) = 0.096 au; DI = 0.628) that has an increased BCP ellipticity (ε = 0.213) and a higher negative charge on H3.^18^ The hydrides of the stronger terminal Zn2–H4 and Zn1–H5 σ-bonds (ρ(r) = 0.115 au; DI = 0.821; ε = 0.006) also exhibit large negative charges. No Ru–Zn bond paths are seen despite the Ru–Zn distances (Ru1–Zn1 = 2.5167(6) Å, Ru1–Zn2 = 2.4881(6) Å) being well within the sum of the covalent radii (2.68 Å).^43^ Apparently the hydrides in **5**^**+**^ perturb the topology of the electron density sufficiently to lose the Ru–Zn bond path; in contrast the direct Ru–Zn bonds in [Ru(PPh_3_)_2_(Ph_2_PC_6_H_4_)(ZnMe)_2_]^+^ have similar Ru–Zn distances and do exhibit Ru–Zn bond paths.^16^ The average Ru···Zn DI value of 0.382 indicates significant Ru···Zn interactions are retained in **5**^**+**^.

The ^1^H NMR spectrum of a crystalline sample of **5** redissolved in THF-*d*_8_ displayed three highly coupled hydride resonances in a relative ratio of 1:2:2 (Figure S1–S2). The hydride trans to CO appeared as a triplet of triplets at δ −8.94 with a ^2^*J*_HH_ coupling to the two Ru–H–Zn hydrides of 5 Hz and a splitting of 16 Hz to the two terminal ZnH. It exhibits a *T*_1_ value of 620 ms (298 K; 599 ms at 233 K; 400 MHz). The two Ru–H–Zn bridging hydrides appeared at δ −9.48 as a somewhat distorted doublet of triplets, with a similar *T*_1_ value (733 ms (298 K), 695 ms (233 K); 400 MHz). An unusual looking multiplet at δ 4.79^12,44^ (*T*_1_ = 1100 ms (298 K), 777 ms (233 K); 400 MHz) was seen for the ZnH ligands. Successful simulation (Figure S3) of the resonance was achieved upon inclusion of an exchange process between the Ru–H and Ru–H–Zn hydrides, a process that was verified by EXSY (Figure S4) and computational studies (vide infra). No exchange processes involving the ZnH ligands themselves were detected. In addition to an obvious ν(CO) band, the IR spectrum of **5** (Figure S6) displayed weaker, lower frequency features at ca. 1870 and 1600 cm^–1^, which we attribute to ν(ZnH) and ν(RuH) by comparison to calculated frequencies.

**Scheme 3:**
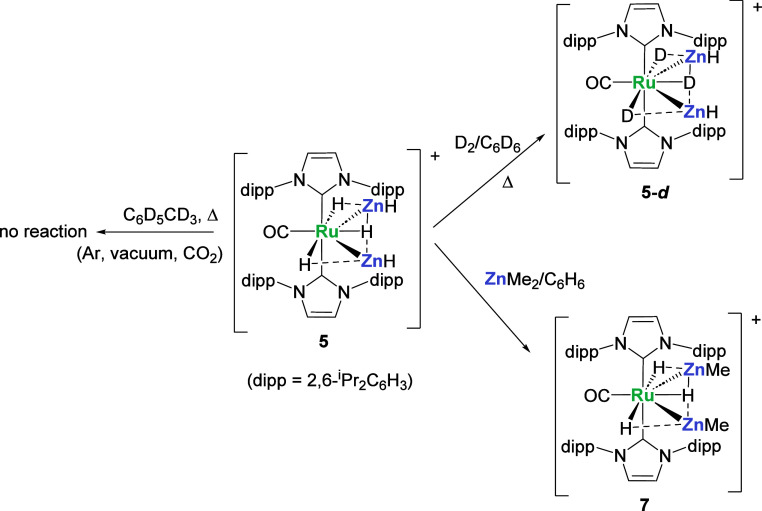
Reactivity of **5** in Aromatic Solvents. [BAr^F^_4_]^−^ anions are omitted for clarity.

Arene solutions of **5** were unchanged upon heating to 60 °C under Ar, vacuum, and even CO_2_ (Figure S7). In contrast, H/D exchange to give **5**-**d** occurred at 60 °C under D_2_ (Scheme 3, Figure S8) as shown by the broadening and weakening in intensity of the Ru–H resonances in the ^1^H NMR spectrum (corresponding signals were apparent in the ^2^H NMR spectrum) and formation of free HD (δ 4.42; 1:1:1 triplet,^1^*J*_HD_ = 43 Hz). The Zn–H resonance became more complex in appearance, which we attribute to overlapping *J*_Ru–H_ and *J*_Ru-D_ splittings; there was no evidence for deuteration at the ZnH sites by ^2^H NMR spectroscopy.

**Figure 2:**
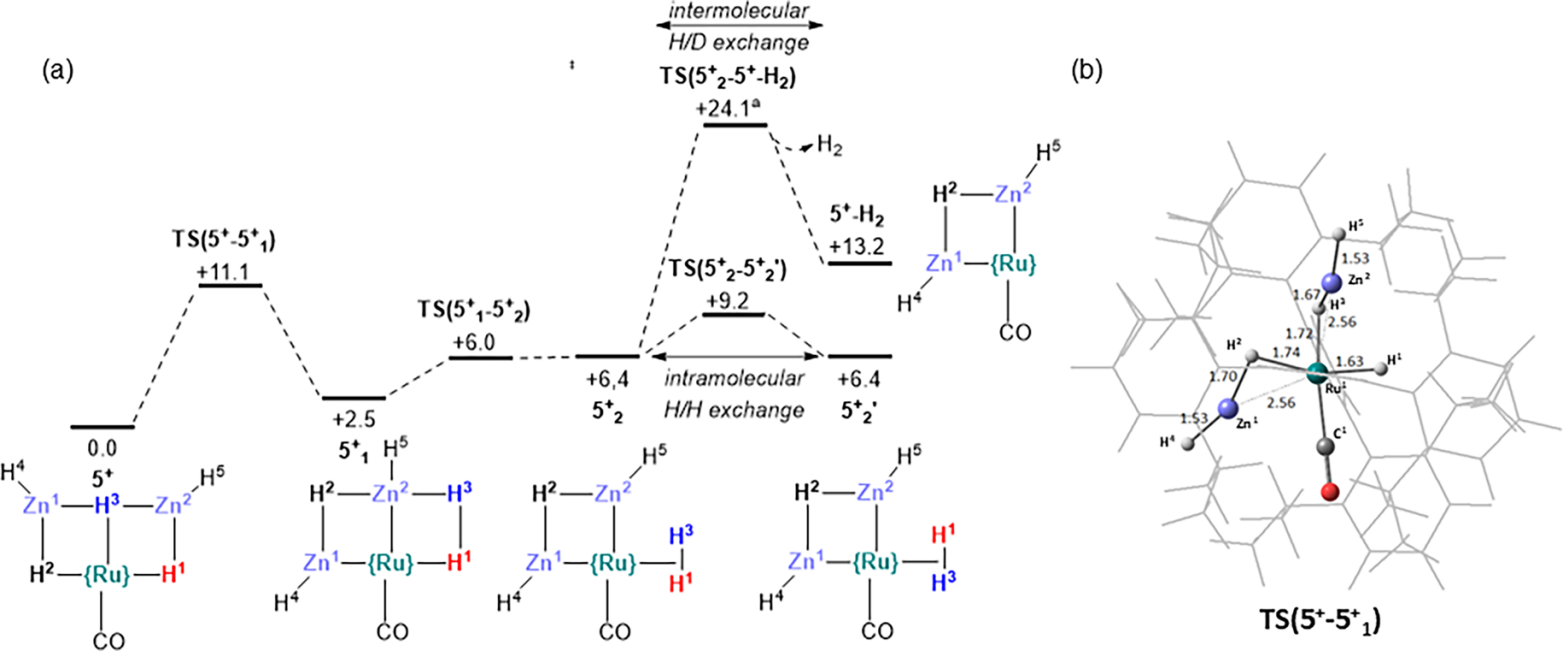
(a) Computed free energy profiles (BP86-D3(PCM = THF)/Def2-TZVP)//BP86-SDD(Ru,Zn), 6-31G**; kcal/mol) for intramolecular H-exchange and intermolecular H/D exchange in **5**^**+**^ (axial IPr ligands are omitted for clarity). (b) Computed geometry of **TS(5**^**+**^**-5**^**+**^_**1**_**)** highlighting selected distances (Å); participating atoms in ball and stick mode, spectator NHC ligands depicted as wireframe. ^a^The overall barrier recomputed in benzene (the solvent used experimentally for H/D exchange studies) was 24.2 kcal/mol.

Possible mechanisms for both intramolecular H/H exchange and the related intermolecular H/D exchange in **5**^**+**^ were modeled computationally (Figure 2). As seen in previous studies,^17,18^ H/ZnR exchange can readily occur, here via **TS(5**^**+**^**-5**^**+**^_**1**_**)** with a barrier of 11.1 kcal/mol that exchanges H^3^ and Zn^2^H^5^ to place H^3^ adjacent to H^1^ in **5**^**+**^_**1**_. Unusually, this transition state entails a second H/ZnR exchange, with H^2^ and Zn^1^H^4^ also swapping positions. This may be driven by positioning of H^2^ between the two ZnH ligands in **5**^**+**^_**1**_ and the resultant stabilizing Zn^δ+^···H^δ-^ interactions.^18^ H^3^ and H^1^ then couple to form a dihydrogen ligand that can rotate via **TS(5**^**+**^_**2**_**-5**^**+**^_**2**_**’)** at +9.2 kcal/mol. The microscopic reverse steps then return the system to **5**^**+**^ in which H^3^ and H^1^ have exchanged positions. The overall barrier for this process is 11.1 kcal/mol, consistent with the room temperature exchange seen experimentally; an analogous series of events starting with H^2^/Zn^1^H^4^ exchange will lead to H^3^/H^2^ exchange. H_2_/D_2_ exchange can also proceed from **5**^**+**^_**2**_ via the H_2_ dissociation transition state **TS(5**^**+**^_**2**_**-5**^**+**^**-H**_**2**_**)** at +24.1 kcal/mol. D_2_ addition and reversible H/D exchange via the intramolecular pathway would then account for the formation of HD. Intermolecular exchange has a barrier of 24.1 kcal/mol and is therefore considerably harder than intramolecular exchange, as is the case experimentally.

Addition of an excess of ZnMe_2_ (2.5 equiv) to a benzene solution of **5** resulted in substitution to give the bis-ZnMe analogue [Ru(IPr)_2_(CO)(ZnMe)_2_H_3_][BAr^F^_4_] (**7**, Scheme 3), which started to precipitate within a few minutes of mixing.^45^ The ^1^H NMR spectrum of **7** (THF-*d*_8_) revealed two Ru–H resonances with very similar chemical shifts and *T*_1_ values (δ −8.6 (*T*_1_ = 725 ms (298 K), 723 ms (233 K); δ −9.3 (*T*_1_ = 732 ms (298 K), 616 ms (233 K)) to those of **5**. Both signals were broad at room temperature (Figure S9) and shown to be in exchange by EXSY (Figure S12). Cooling to <253 K (Figure S10) resolved them to a triplet and doublet respectively (with a mutual ^2^*J*_HH_ coupling of 4.6 Hz) in a 1:2:6 relative integral ratio with a singlet at δ −0.8 for the two ZnMe groups. This facile ZnH/ZnMe substitution chemistry is reminiscent of that displayed by another Ru–Zn heterometallic complex, [Ru(PPh_3_)_2_(ZnMe)_4_H_2_], which we reported undergoes replacement of all four ZnMe ligands by ZnPh groups (also at room temperature) upon treatment with ZnPh_2_.^17^ A transition state for the first ZnMe/ZnH exchange process, analogous to that for ZnMe/ZnPh exchange in our previous study, was located and gave a barrier of 23.0 kcal/mol (Figure S39).

### Formation of [Ru(IPr)_2_(CO)(ZnH)H_3_] (6)

Given the 6–7 h time scale needed for complete conversion through to **5**, ^1^H NMR spectroscopy was employed to interrogate the early stages of the reaction (Figure S17). Some residual **1** and small amounts of **5** could be seen within 30 min of mixing **1** and ZnH_2_, but the major species was the neutral, mono-ZnH complex [Ru(IPr)_2_(CO)(ZnH)H_3_] (**6**, Scheme 2). Over ca. 1–3 h, **6** depleted, leaving **5** as the dominant species after ca. 7 h.

In the room temperature ^1^H NMR spectra of these crude solutions, the ZnH and three RuH resonances of **6** were all broad, but sharpened at 246 K into four sharp, highly coupled signals in a relative ratio of 1:1:1:1 (Figure S17-Figure S22). The ZnH resonance appeared at δ 5.6 as a doublet of doublet of doublets, with large couplings (26.8 and 19.3 Hz) to the two bridging Ru–H–Zn hydrides at δ −7.4 and −7.6, and a small coupling of 5.5 Hz to the terminal Ru–H at δ −7.9.^46^ The room temperature spectra of **6** using (i) material isolated from “quenched” reactions (i.e., reactions evaporated after ca. 40 min and then extracted into benzene) or (ii) redissolved crystalline material (vide infra) still showed quite broad resonances at δ −7.6 and −7.9, but a sharpened doublet at δ −7.4 and a distorted multiplet at δ 5.6 (Figure S20). In both cases, four sharp resonances appeared in the low temperature spectra.

**Figure 3:**
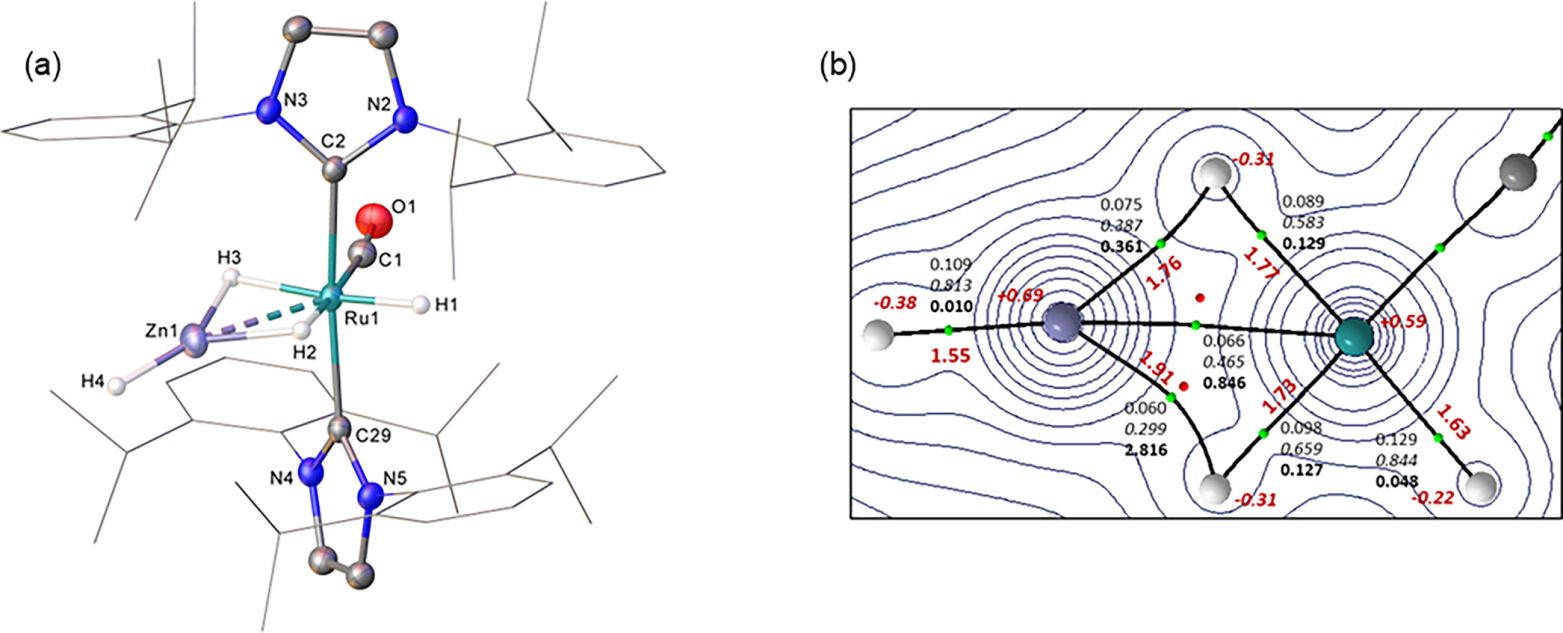
(a) Molecular structure of **6**. Ellipsoids are displayed at 30% probability, the hydrogen atoms (hydrides excepted) are omitted for clarity and the NHC substituents displayed as wireframes for visual ease. Solvent and the minor disordered component have also been omitted. (b) QTAIM molecular graph (X-ray structure with optimized H atom positions) with density contours in the {Zn1Ru1H3} plane with computed Ru–H and Zn–H distances (Å) and QTAIM atomic charges in red. BCP (green spheres) ρ(r) values (au) are in plain text, delocalization indices in italics and, for bond paths to hydrogens, ellipticities in bold. Ring critical points are shown as red spheres.

Serendipitous isolation of a small crop of X-ray quality single crystals provided unequivocal confirmation of the structure of **6** by X-ray crystallography (Figure 3a). The computed geometry and QTAIM molecular graph of **6**^**+**^ (Figure 3b) show H2 and H3 bridging the Ru1–Zn1 vector with H3 (trans to H1) more symmetrically positioned than H2 (trans to CO). Both H2 and H3 exhibit higher negative charges (i.e., bridging character) than H1 and H2 in **5**^**+**^, while H1 in **6**^**+**^ has the lowest charge, reflecting dominant terminal character in that case. In contrast, the properties of the terminal Zn1–H4 bond in **6**^**+**^ are very similar to the Zn1–H4 and Zn2–H5 bonds in **5**^**+**^. A Ru–Zn bond path is seen in **6**^**+**^ with a corresponding DI of 0.465; this higher value compared to **5**^**+**^ (average Ru···Zn DI = 0.382) reflects the shorter experimental Ru–Zn distance of 2.4117(8) Å.

### Relationship of 5 and 6

To account for the formation of neutral **6** as the first-formed product from salt **1** and ZnH_2_, together with the need for an excess of the latter, we propose that the reaction proceeds by initial formation of an unobserved cationic [Ru(ZnH_2_)_2_]^+^ species, which upon loss of [ZnH][BAr^F^_4_], generates **6**. The formation of [ZnH]^+^ was confirmed upon repeating the reaction of **1** and ZnH_2_ in the presence of tmeda (Me_2_NCH_2_CH_2_NMe_2_). In addition to the ^1^H NMR signals for **6**, there was a ZnH resonance at δ 3.67 (Figure S23) matching that reported for [(tmeda)ZnH(THF)][BAr^F^_4_].^47^ As the subsequent transformation of **6** through to **5** was slowed down (only small amounts of the latter were apparent even after 2 days (Figure S23)), it suggests that transformation of **6** into the final salt **5** results from reaction of the initially eliminated [ZnH][BAr^F^_4_].

In contrast to the stability of **5** in aromatic solvents noted above, it proved less stable in THF, yielding ^1^H NMR resonances for **6**, as well as the imidazolium salt [IPrH][BAr^F^_4_], over 12–72 h at room temperature (Figure S25–S26). Addition of tmeda had no effect on these processes, nor did it afford any [(tmeda)ZnH(THF)][BAr^F^_4_] (Figure S24). In a further contrast, THF solutions of the ZnMe analogue **7** were still intact after a week at room temperature (Figure S15).

### Catalytic Hydrogenation and Isomerization of Alkenes

The resurgence of interest in TM-MGM heterometallic chemistry over the past few years stems largely from the potential of the different metal centers to act cooperatively in catalysis.^48−50^ In contrast to the extensive studies of TM-group 13 element heterometallics,^51−53^ there have been very few reports of catalysis with TM-Zn systems,^54−58^ although it is known that ZnR-ligands can influence catalytically relevant process, such as reductive elimination.^59−62^

Salt **5** proves ideal for catalytic testing. Parallel studies with hydride **1** provide a benchmark for establishing cooperative effects from Zn, while further comparisons with **7** and [Ru(PPh_3_)_3_(ZnMe)_2_H_3_][BAr^F^_4_] allow us to probe the influence of ZnH vs ZnMe and IPr vs PPh_3_ ligands. Given the known reactivity of [Ru(NHC)_2_(CO)H]^+^ for alkene hydrogenation and isomerization,^63^ these processes were tested for both 1-hexene and 5-hexene-2-one with all four compounds. Reactions were carried out in J. Young’s resealable NMR tubes and followed by the depletion of the alkene resonances in the ^1^H NMR spectra (Figures S28–S34). The results are summarized in Table 1.

**Table 1:** Preliminary Studies of Catalytic Alkene Hydrogenation and Isomerization by **1**, **5**, **7** and [Ru(PPh_3_)_3_(ZnMe)_2_H_3_][BAr^F^_4_].^a^^,^^b^

	**1**		**5**		**7**^d^	**[Ru(PPh**_**3**_**)**_**3**_**(ZnMe)**_**2**_**H**_**3**_**][BAr**^**F**^_**4**_**]**^e^
	THF	benzene	THF	benzene	THF	benzene
1-Hexene						
Hydrogenation	15/100/0.66^c^	40/100/0.25	15/100/0.66	3.3/100/3	-	-
Isomerization	0.6/100/18	0.6/100/18	0.09/37/42	0.25/100/40	-	0.03/28/90
5-Hexene-2-one						
Hydrogenation	40/100/0.25	20/100/0.5	40/100/0.25	-	2.5/100/4	-
Isomerization	0.14/25/18	0.14/26/18.5	-	0.34/72/21	-	0.38/100/26

a 10 mol % [Ru] complex, 0.45 mL THF-*d*_8_ or C_6_D_6_, 298 K.

b 1 atm H_2_ used for hydrogenations.

c Values of *turnover frequency* (TON/h^–1^), % conversion and time (h) for loss of alkene (measured by ^1^H NMR spectroscopy) vs 1,3,5-(MeO)_3_C_6_H_3_ as internal standard.

d Insolubility precluded studies of **7** in C_6_D_6_.

e [Ru(PPh_3_)_3_(ZnMe)_2_H_3_][BAr^F^_4_] rapidly decomposed in THF (Figure S27).

Under a set of standard conditions (10 mol % Ru precursor, 1 atm H_2_, room temperature), 1-hexene and 5-hexene-2-one were fully hydrogenated to hexane and 2-hexanone^64^ respectively by **5** and **1** in both THF-*d*_8_ and C_6_D_6_.^65^ The former has somewhat lower activity in benzene as a result of its slightly lower solubility. There is no clear evidence from the TOF values for the ZnH ligands providing any cooperative enhancement of catalysis. At the same time, the notable difference in activity of **5** and **7** with 5-hexene-2-one supports there being some influence of the Zn substituent; further studies are required to probe this in more detail. It is also clear from the presence of intact **5** at the end of a reaction with this substrate (Figure S31), that the Lewis acidic ZnH groups do not compromize catalysis with donor-bearing substrates.

Although all four compounds show some ability to isomerize alkenes (1-hexene to cis-/trans-2-hexene; 5-hexene-2-one to cis-/trans-4-hexene-2-one; Figure S32–S33),^20,21^ the activity is only low in all cases,^66^ with the long reaction times leading to clear detrimental catalyst degradation processes in some cases (Figure S33).

## Conclusions

Herein, we report what we believe to be the first use of ZnH_2_ for the preparation of heterometallic complexes featuring a transition metal center.^67,68^ Neutral [Ru(IPr)_2_(CO)(ZnH)H_3_] (**6**) forms as the initially observed product following addition of excess ZnH_2_ to [Ru(IPr)_2_(CO)H][BAr^F^_4_] (**1**) in a reaction that generates [ZnH][BAr^F^_4_], as proven by the trapping of the cation with tmeda. [ZnH]^+^ reacts onward to afford [Ru(IPr)_2_(CO)(ZnH)_2_H_3_][BAr^F^_4_] (**5**) as the final product of the reaction. Experimental and computational methods show that both the structure and reactivity of **5** are in line with recent reports from our groups on TM(Zn)_2_ heterometallic compounds.^16−18^ Thus, the three Ru-hydrides are predominantly terminal in character but bridge to varying degrees with the ZnH ligands. These Ru-hydrides also readily exchange positions with the ZnH ligands, a process that is facilitated by the formation of stabilizing electrostatic RuH^δ-^···ZnH^δ+^ interactions. Although H/D exchange in **5** proves quite challenging, we observe ZnH/ZnMe metathesis at room temperature within minutes. Lastly, we demonstrate that **5** exhibits some aptitude for the catalytic hydrogenation of alkenes, thereby providing a rare example of TM-Zn-mediated catalysis.

It is notable that the facile nature of both the ZnR exchange and hydrogenation chemistry take place despite the coordinative saturation of **5**, suggesting that it is the unsaturation of the Zn ligand that is of particular importance. In this respect, the use of ZnH_2_ for the synthesis of new TM-Zn heterometallics could offer real benefits over more widely used Lewis-base stabilized Zn–H reagents.^55,57,69−71^

## Supporting Information Available

NMR/IR spectra, computational details (PDF)
Computed geometries (XYZ)

## Supplementary Material

ic4c05360_si_001.pdf

ic4c05360_si_002.xyz
