# Supplementary material for: ZnH2 as a Precursor to Catalytically Active Ru–ZnH Heterometallic Complexes
Source: Inorg Chem. 2025 Feb 19;64(8):4043–51. doi: 10.1021/acs.inorgchem.4c05360 (PMC11881041; doi:10.1021/acs.inorgchem.4c05360)
Supplement: Supplementary file 1 — ic4c05360_si_001.pdf [file ic4c05360_si_001.pdf]

SUPPORTING INFORMATION

ZnH_2 as a Precursor to Catalytically Active Ru-ZnH Heterometallic Complexes

Anne-Frédérique Pécharman,[a] Ambre Carpentier,[b] John P. Lowe,[a] Stuart A. Macgregor,[c] Mary F. Mahon[a] and Michael K. Whittlesey[a*]

[a]*Department of Chemistry, University of Bath, Bath, BA2 7AY, United Kingdom*

[b]*Institute of Chemical Sciences, School of Engineering and Physical Sciences, Heriot-Watt University, Edinburgh EH14 4AS, United Kingdom*

[c]*EaStCHEM School of Chemistry, University of St Andrews, North Haugh, St Andrews KY16 9ST, United Kingdom*

[*](Corresponding author): m.k.whittlesey@bath.ac.uk

Table of Contents

S1. NMR and IR Spectra S2

S2. Computational Studies S37

S2.1. QTAIM Studies S37

S2.2. Computed Reaction Profiles S39

S2.3. Computed Structures and Energies S41

S3. References S62

S1. NMR and IR Spectra

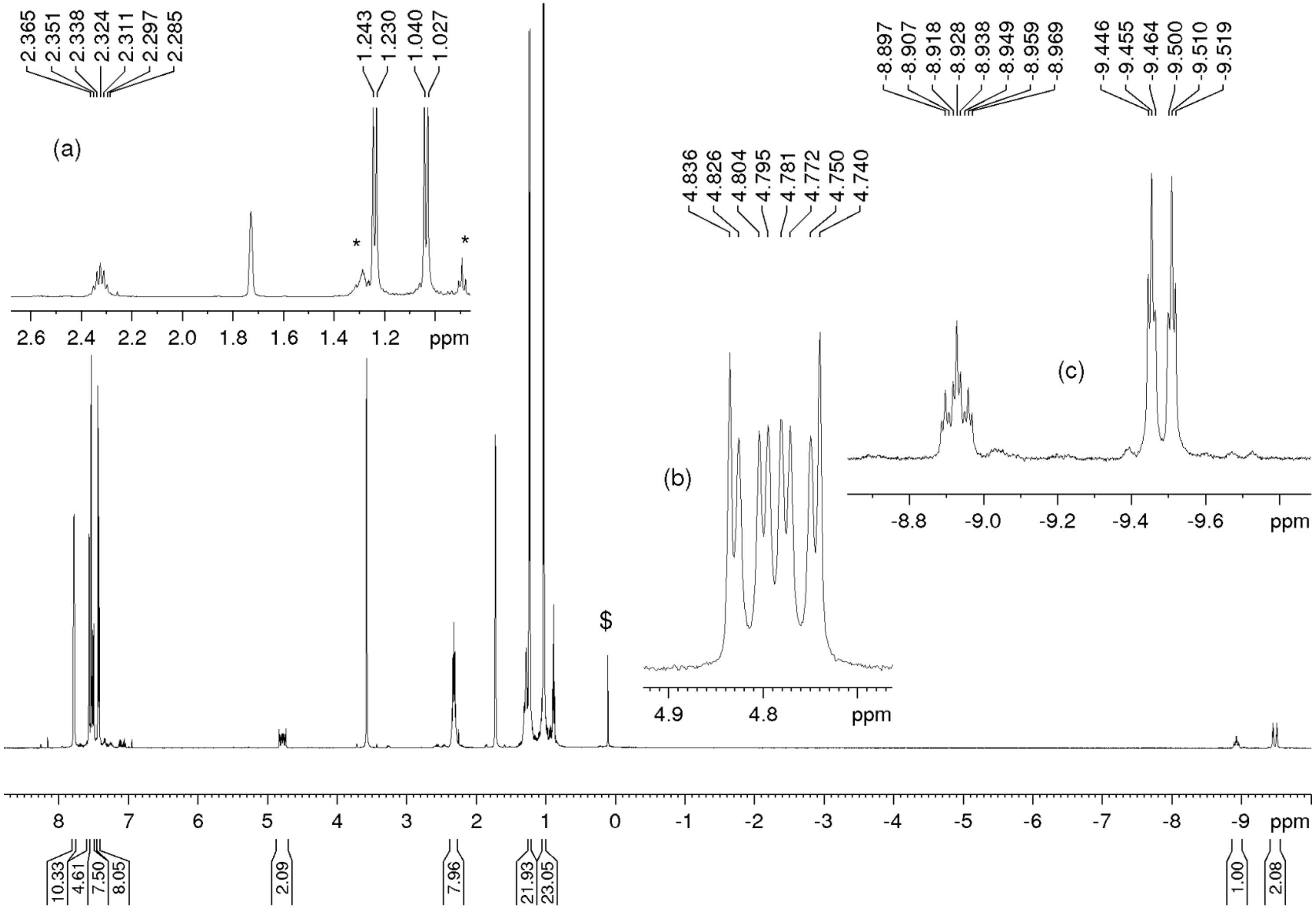

Figure S1. ^{1}H NMR spectrum (THF-d_8, 500 MHz, 298 K) of $[Ru(IPr)_2(CO)(ZnH)_2H_3][BAr^{F}_4]$ (**5**). Insets show expansions of (a) ^{i}Pr, (b) ZnH and (c) RuH regions (* = hexane; $ = Si grease).

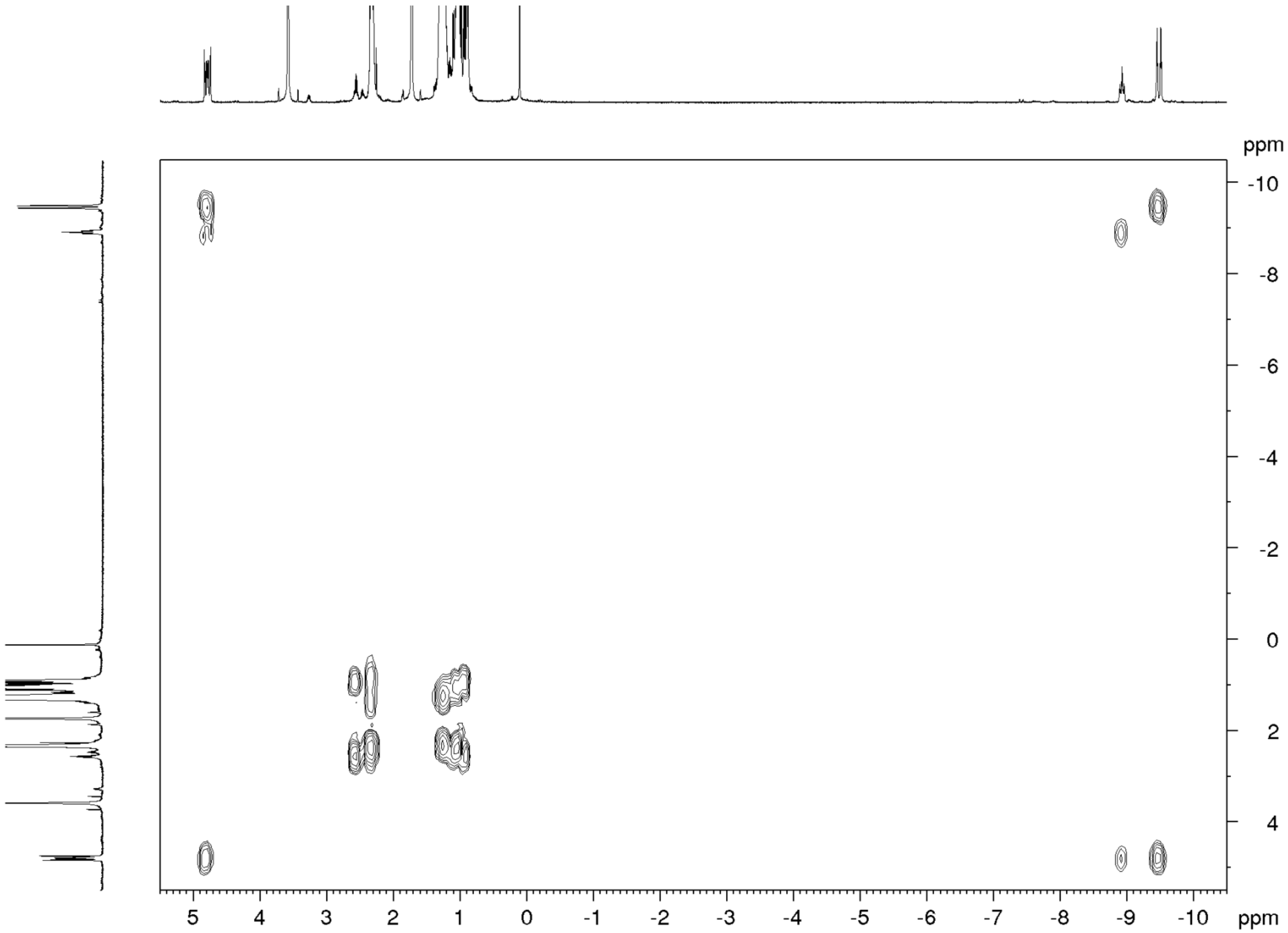

Figure S2. ^{1}H COSY spectrum (500 MHz, THF-d_8, 298 K) of $[Ru(IPr)_2(ZnH)_2H_3][BAr^F_4]$ (**5**), highlighting the coupling between the ZnH (δ 4.8) and RuH (δ −8.9 and −9.5) resonances.

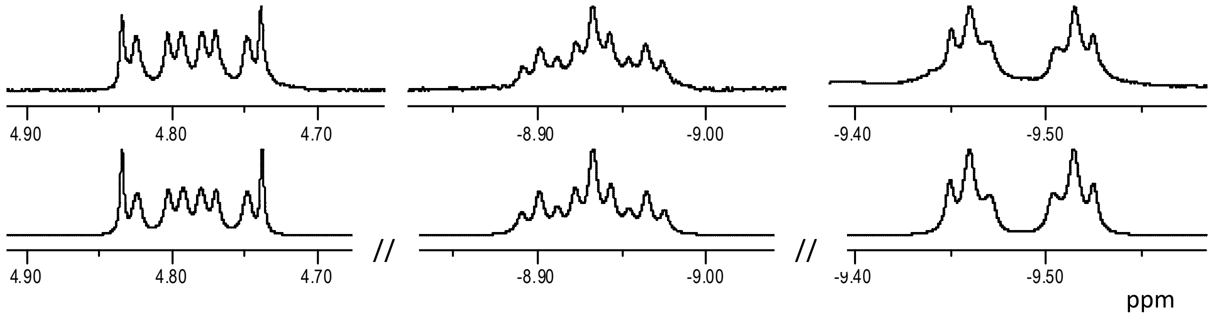

Figure S3. (Top) Experimental and (bottom) simulated (gNMR 5.0) ^{1}H spectrum (500 MHz) of $[Ru(IPr)_2(ZnH)_2H_3][BAr^F{}_4]$ (**5**), showing (left) Zn–*H*, (center) Ru–*H* and (right) Ru–*H*–Zn resonances. Spectra were simulated as an AA'BXX' system where A = Ru–*H*–Zn, B = Ru–*H* and X = Zn–*H*. Data were simulated with AA' = 1.5 Hz, AB = 5.3 Hz, AX = 27.3 Hz, AX' = 5.0 Hz, BX = 15.9 Hz, XX' = 1.0 Hz, and exchange processes between A and B at a rate of 2.5 s^{-1}

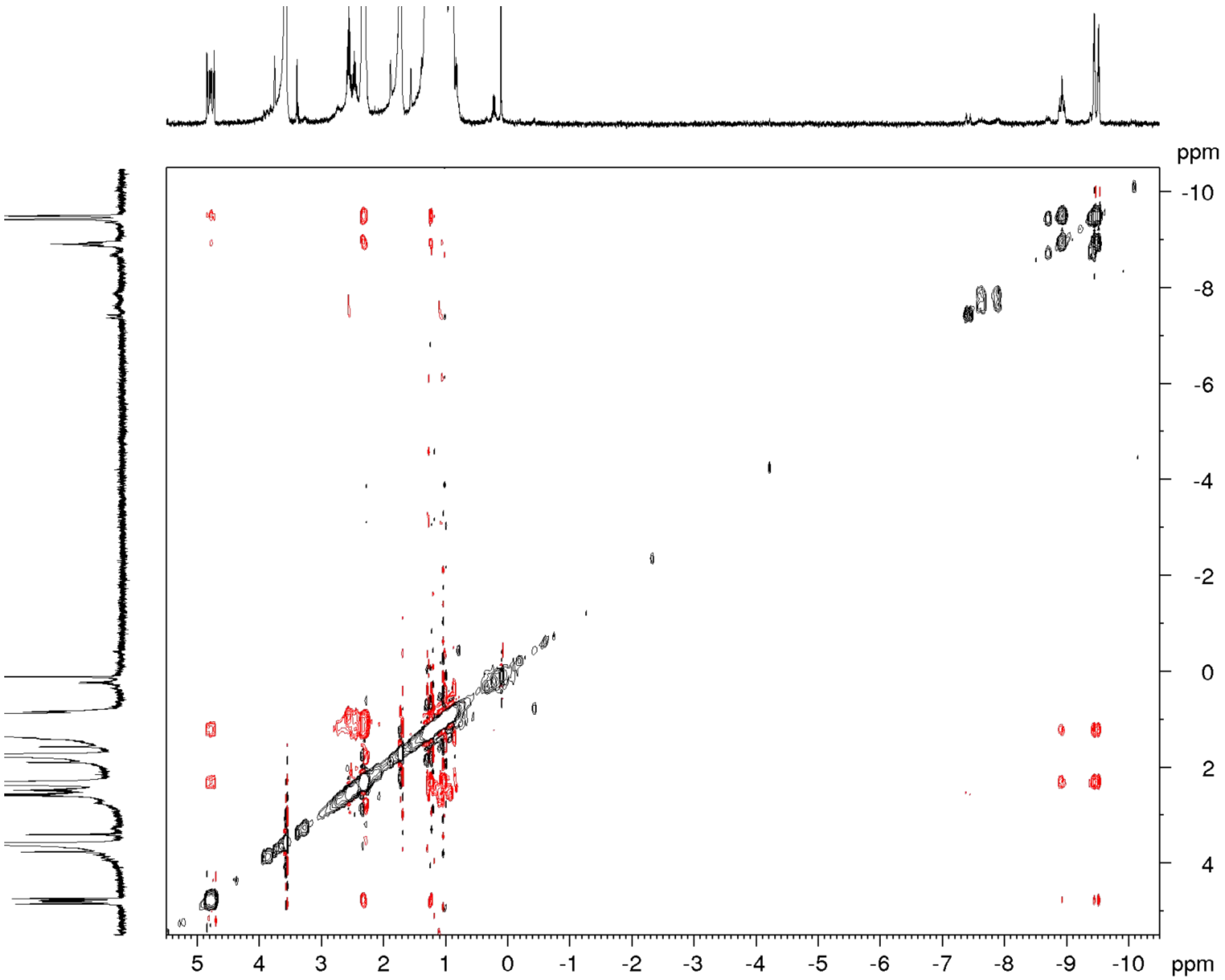

Figure S4. ^{1}H NOESY spectrum (THF-d_8, 400 MHz, 298 K) of [Ru(IPr)$_2$(CO)(ZnH)$_2$H$_3$][BAr$^F{}_4$] (**5**). EXSY peaks between the two RuH signals at δ –8.9 and –9.5 appear in black, with NOESY peaks of both to the ZnH resonance at δ 4.8 shown in red. The batch of sample also contains some [Ru(IPr)$_2$(CO)(ZnH)H$_3$] (**6**), which is responsible for the hydride resonances at δ –7 to –8 (c.f. Figure S17).

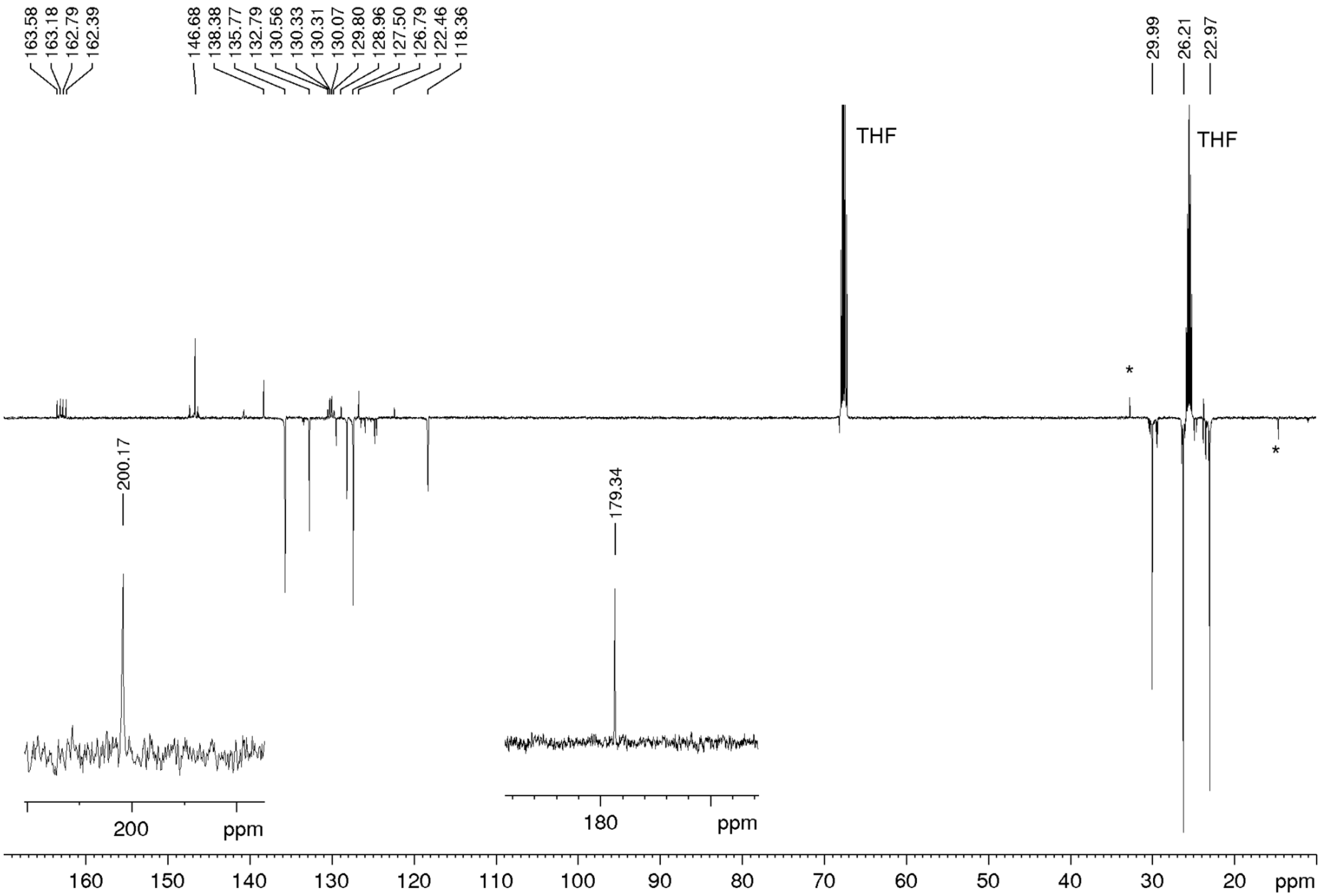

Figure S5. $^{13}C\{^{1}H\}$ DEPTQ NMR spectrum (126 MHz, THF-d_8, 298 K) of $[Ru(IPr)_2(CO)(ZnH)_2H_3][BAr^F{}_4]$ (**5**) (* = hexane). Insets show the high frequency Ru*C*O and RuC_{IPr} resonances.

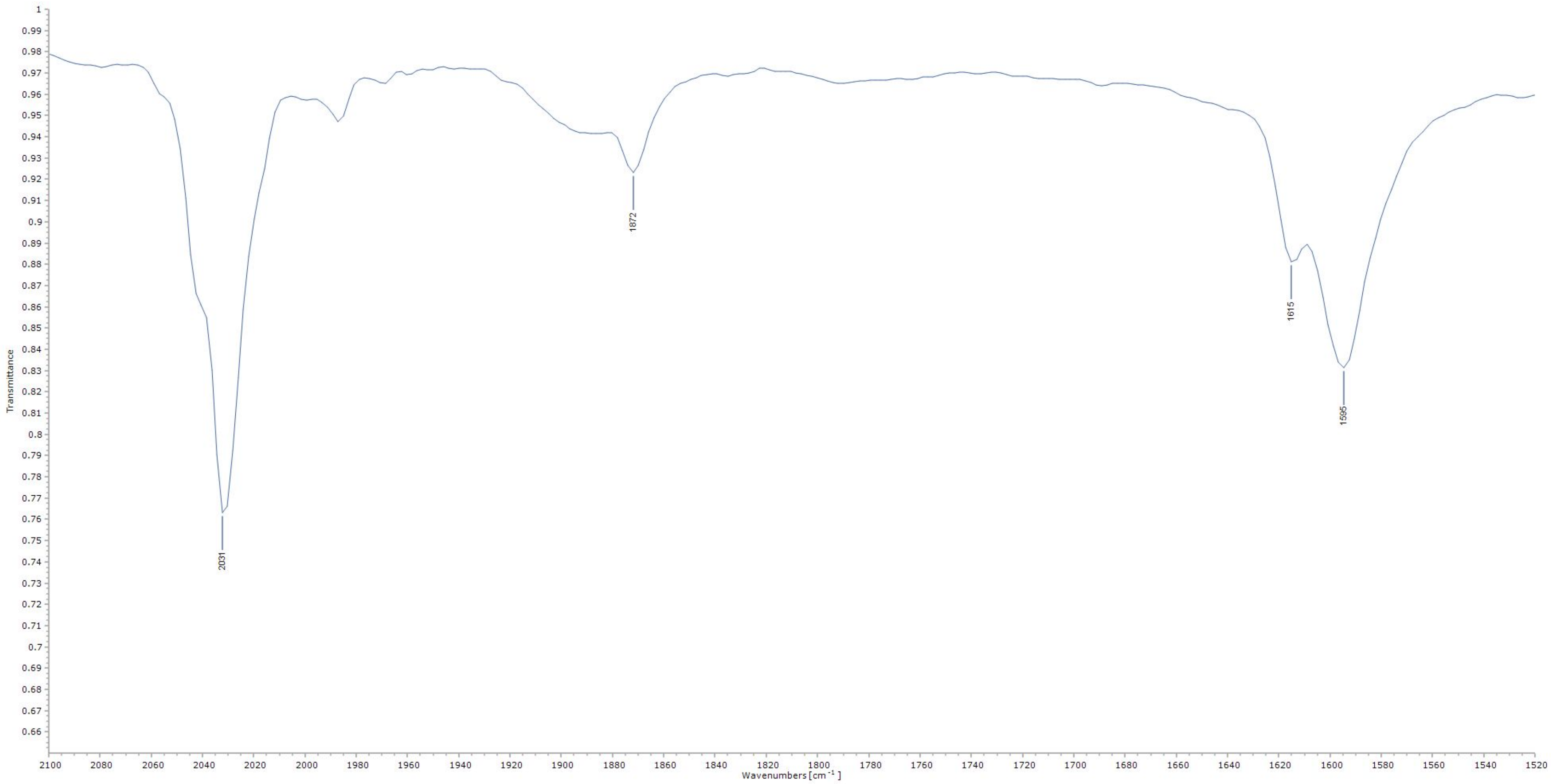

Figure S6. ATR-IR spectrum of $[Ru(IPr)_2(CO)(ZnH)_2H_3][BAr^F{}_4]$ (**5**).

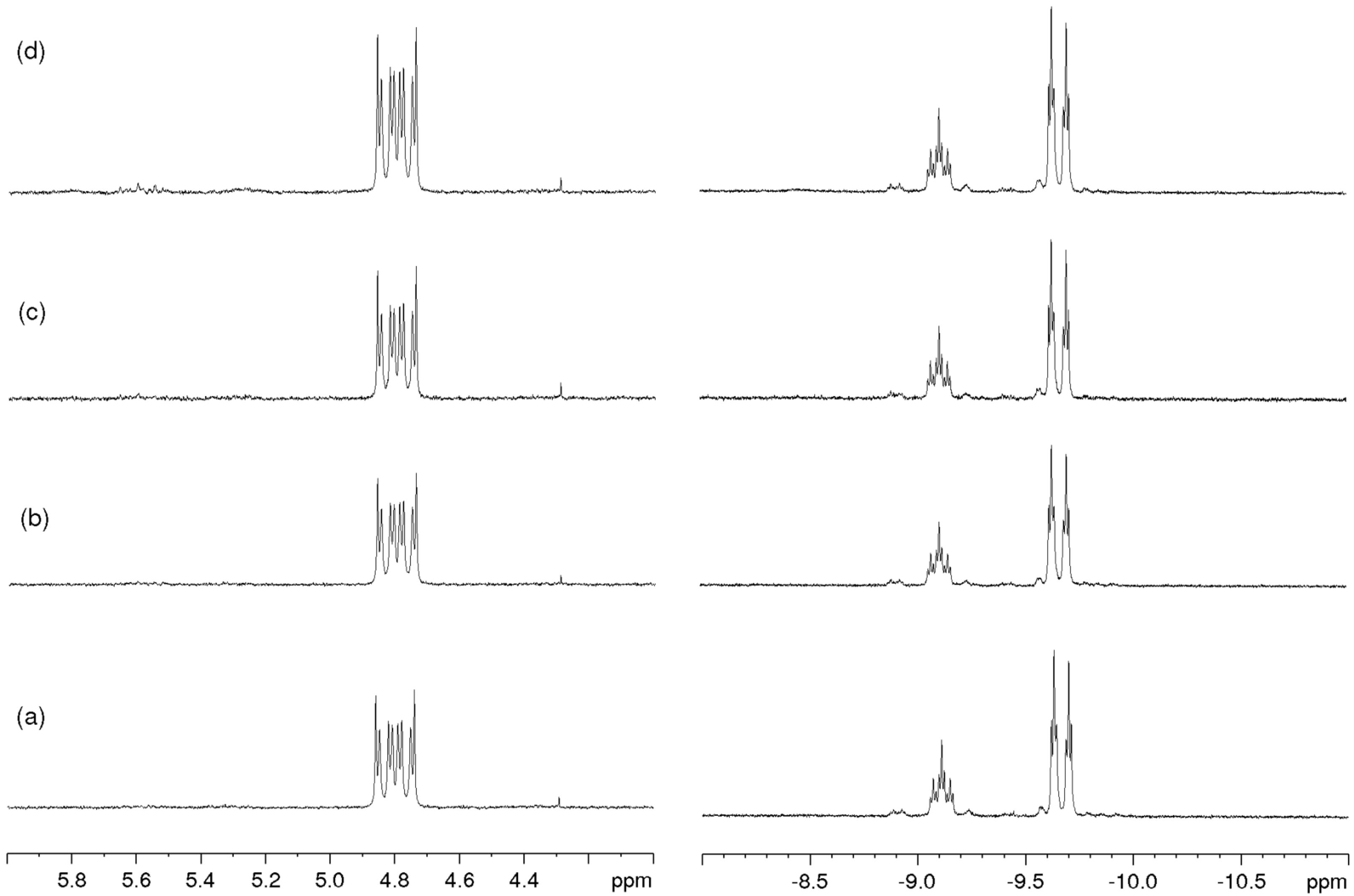

Figure S7. ZnH (left) and RuH (right) resonances (500 MHz, 298 K) of (a) [Ru(IPr)$_2$(CO)(ZnH)$_2$H$_3$][BAr$^F{}_4$] (**5**) following heating for (b) 55 min at 40 °C, then (c) 65 min at 50 °C and (d) a further 30 min at 60 °C in $C_6D_5CD_3$.

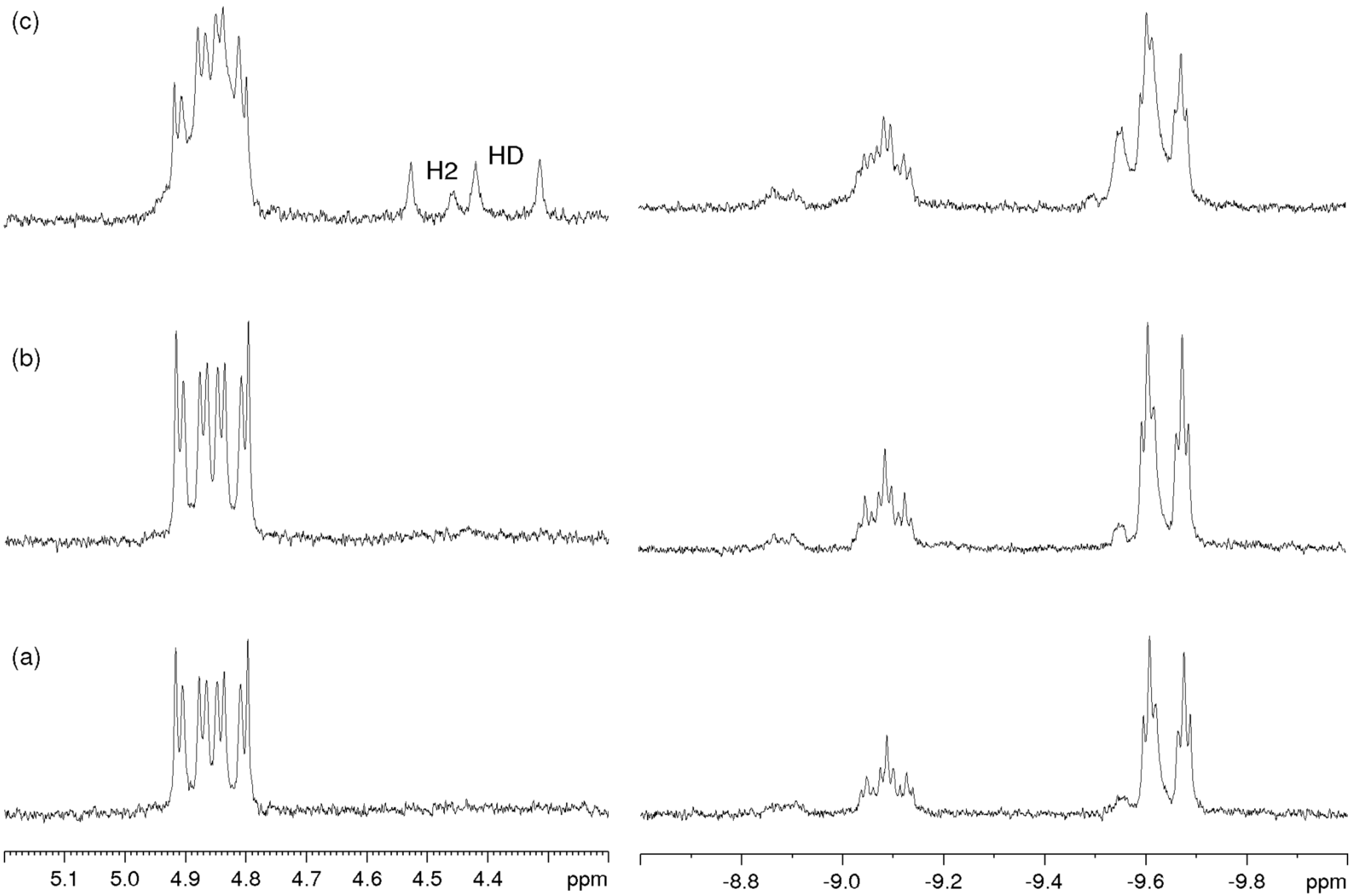

Figure S8. ZnH (left) and RuH (right) resonances of $[Ru(IPr)_2(CO)(ZnH)_2H_3][BAr^F{}_4]$ (**5**) in (a) C_6D_6 (400 MHz, 298 K), (b) after degassing and addition of 1 atm D_2 and (c) after then heating for 120 min at 60 °C.

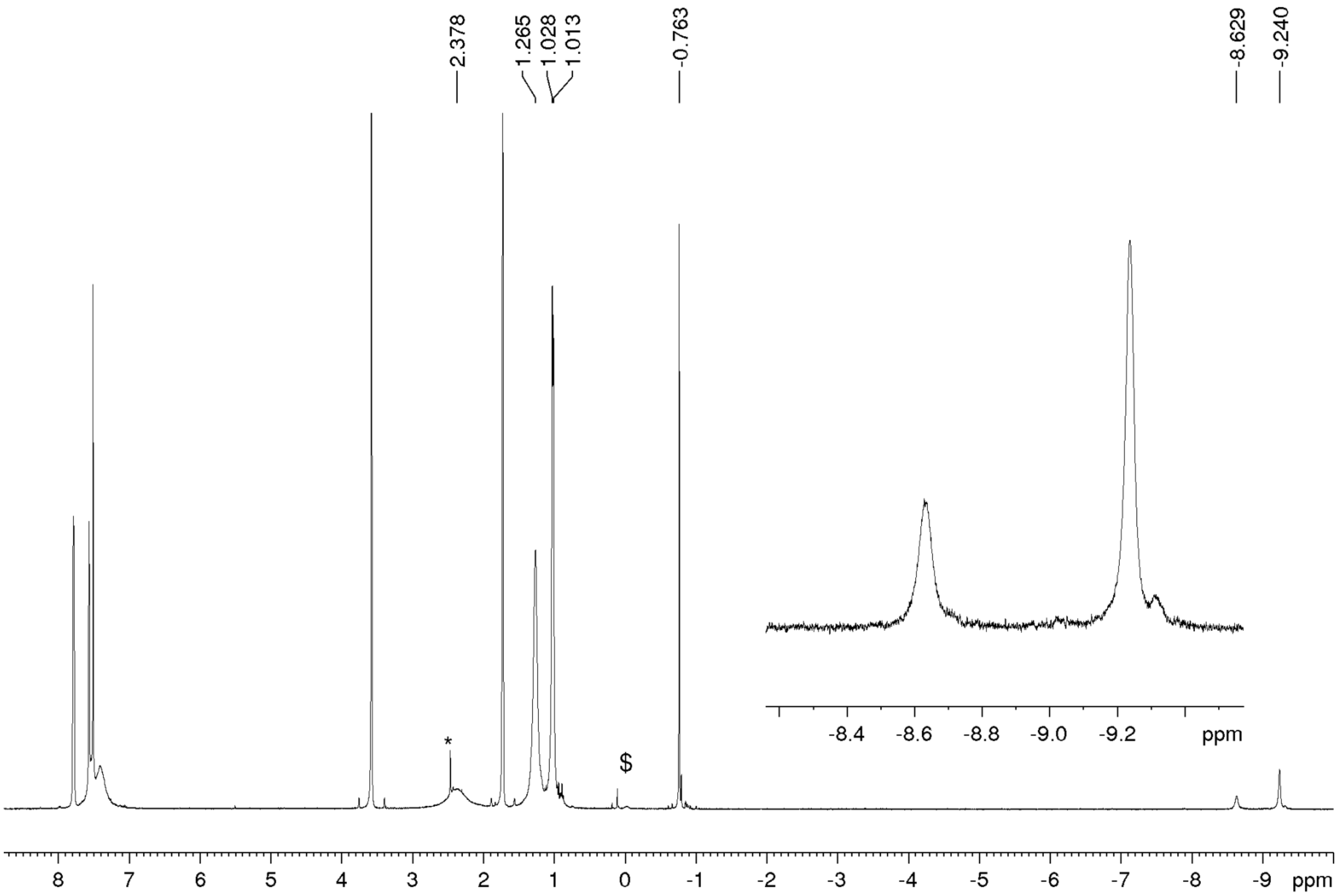

Figure S9. ^{1}H NMR spectrum (THF-d_8, 400 MHz, 298 K) of $[Ru(IPr)_2(CO)(ZnMe)_2H_3][BAr^F{}_4]$ (**7**), with expansion of RuH region shown in the inset (* = unknown impurity; $ = Si grease).

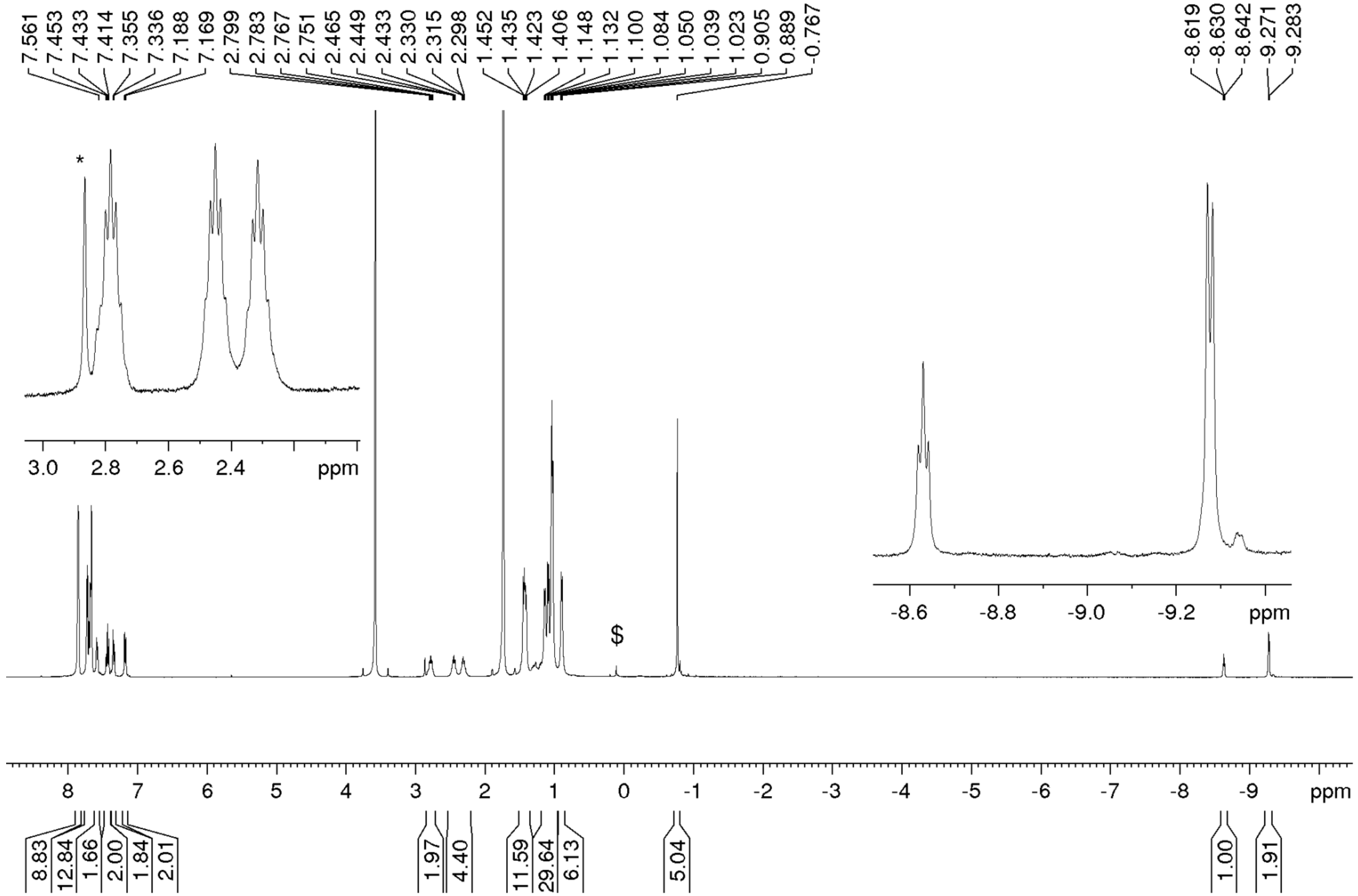

Figure S10. Low temperature (226 K) ^{1}H NMR spectrum (THF-d_8, 400 MHz) of $[Ru(IPr)_2(CO)(ZnMe)_2H_3][BAr^F{}_4]$ (**7**). Insets show expansions of ^{i}Pr methine and RuH regions (* = unknown impurity; $ = Si grease).

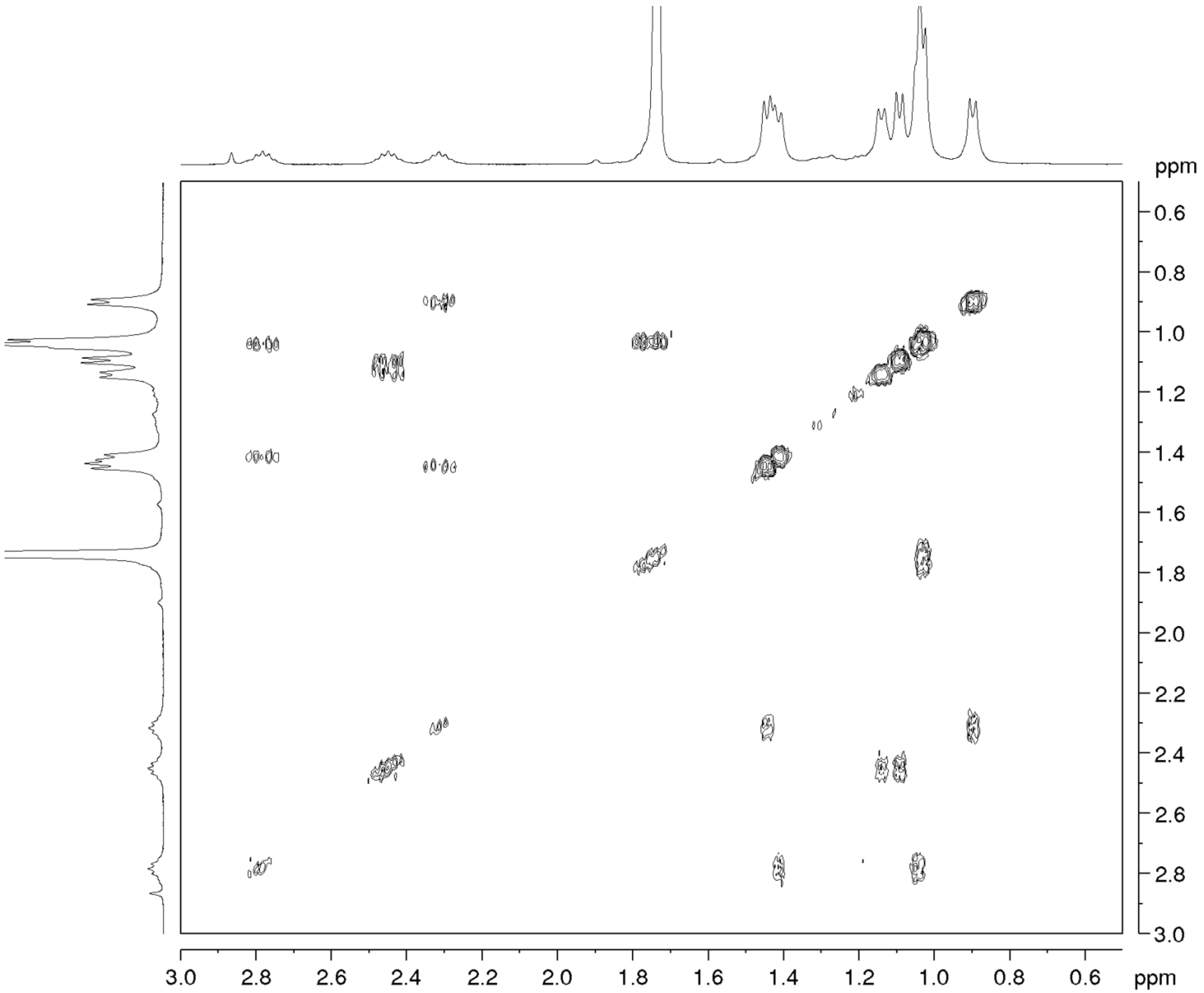

Figure S11. ^{1}H COSY spectrum (THF-d_8, 400 MHz, 226 K) of $[Ru(IPr)_2(CO)(ZnMe)_2H_3][BAr^F{}_4]$ (**7**) confirming the presence one ^{i}Pr methine resonance underneath the THF signal at ca. δ 1.7.

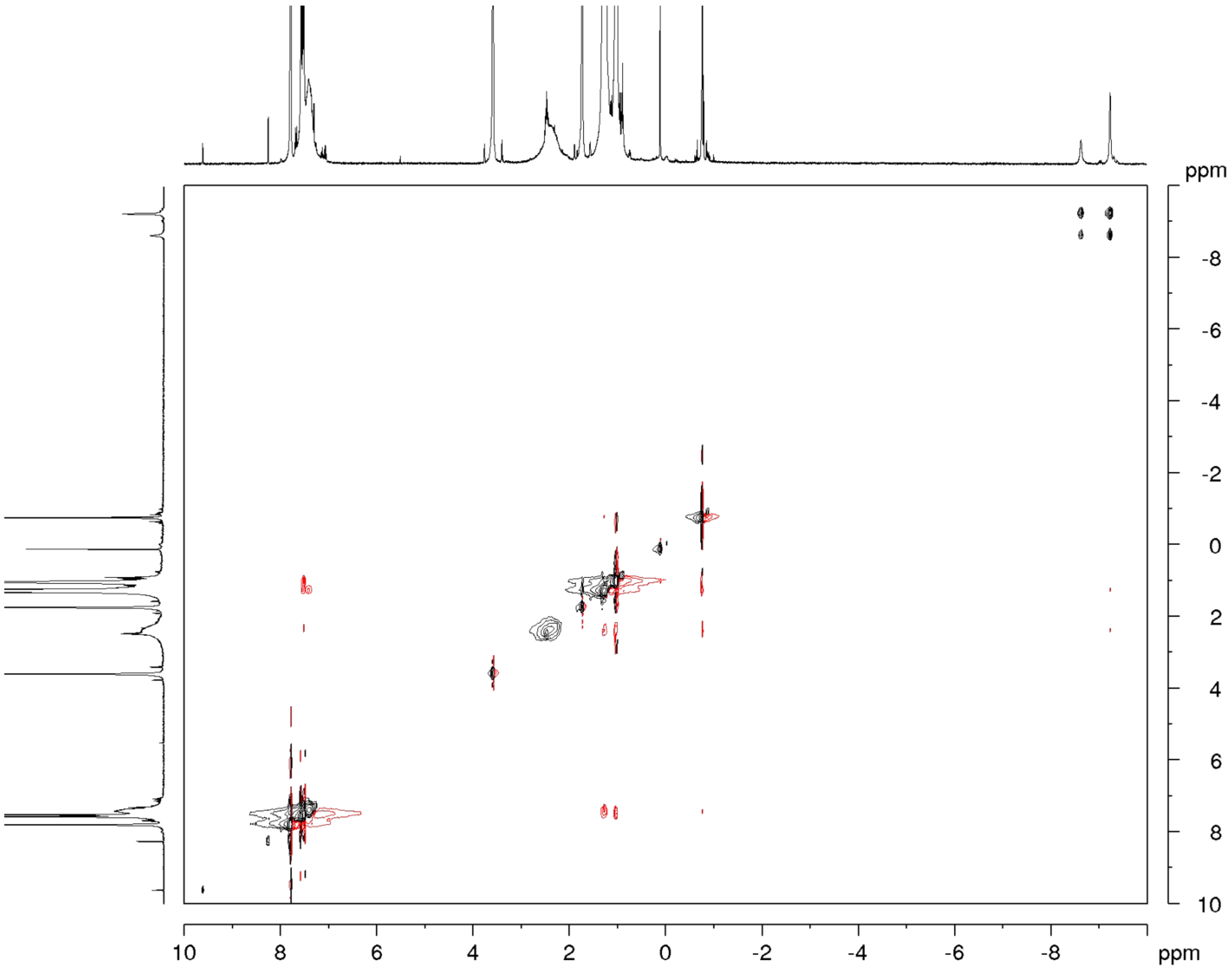

Figure S12. ^{1}H NOESY spectrum (THF-d_8, 400 MHz, 298 K) of $[Ru(IPr)_2(CO)(ZnMe)_2H_3][BAr^F{}_4]$ (**7**). EXSY peaks in black, NOE signals in red.

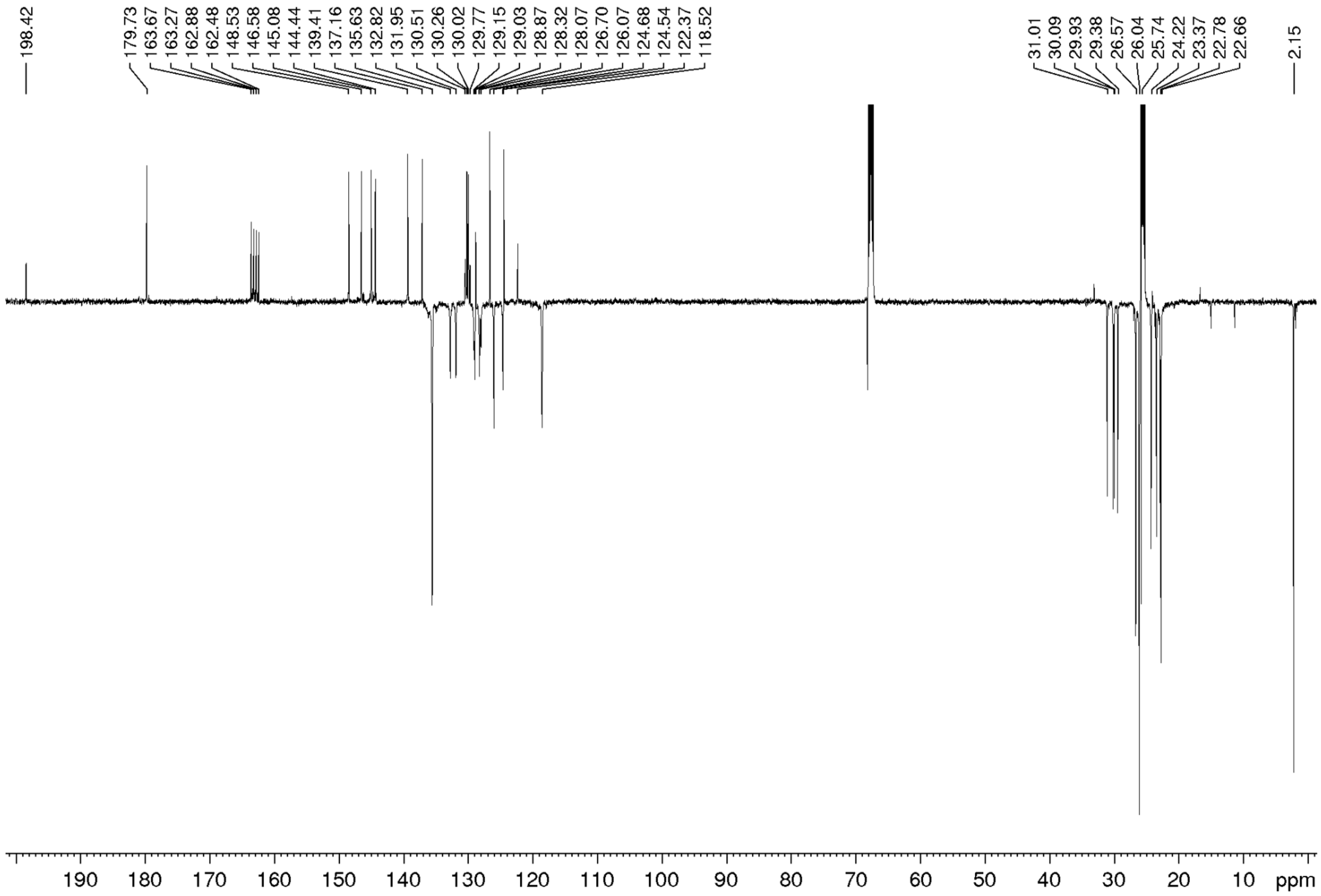

Figure S13. $^{13}C\{^1H\}$ DEPTQ NMR spectrum (THF-d_8, 126 MHz, 231 K) of $[Ru(IPr)_2(CO)(ZnMe)_2H_3][BAr^F_4]$ (**7**).

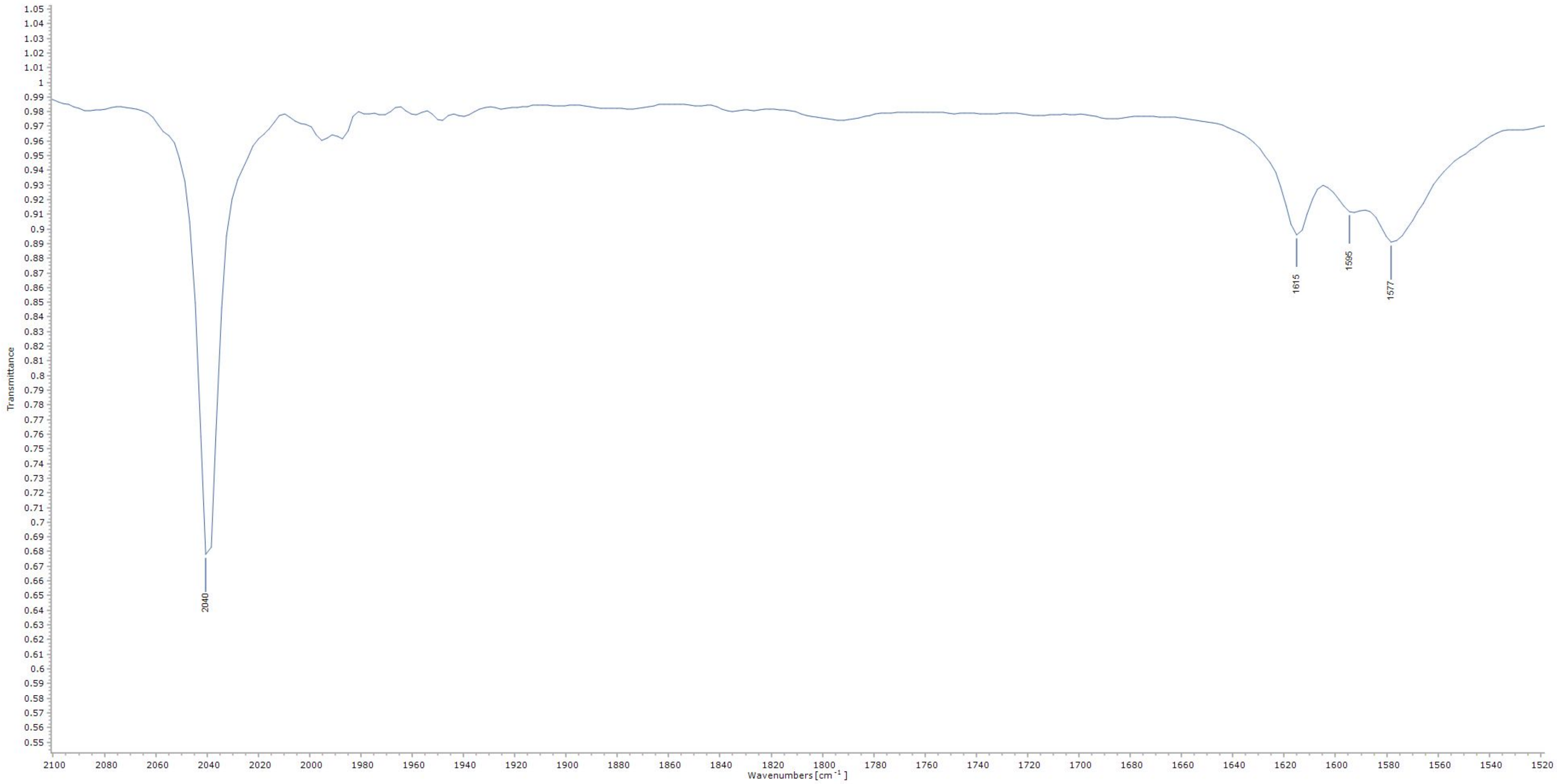

Figure S14. ATR-IR spectrum of $[Ru(IPr)_2(CO)(ZnMe)_2H_3][BAr^F{}_4]$ (**7**).

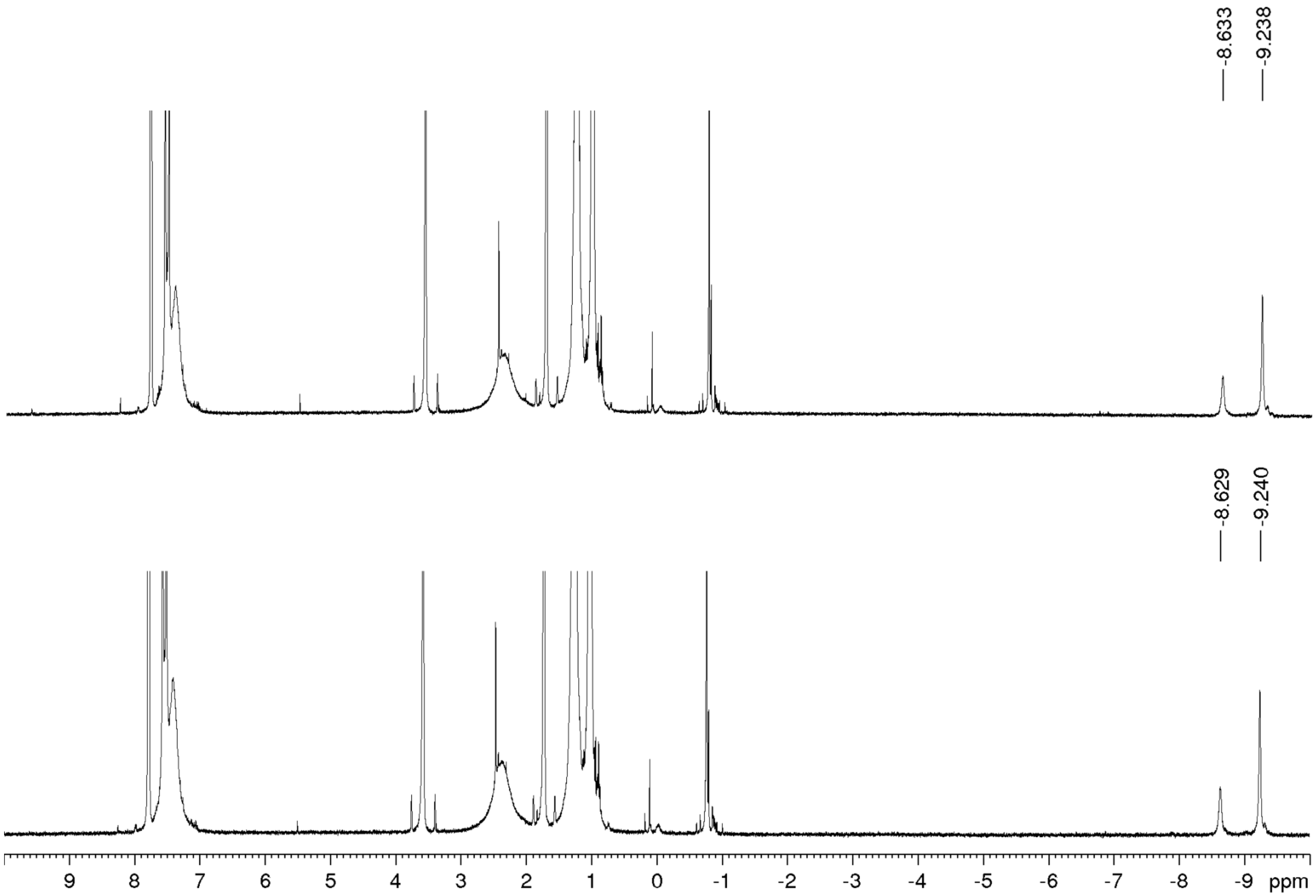

Figure S15. (Bottom) ^{1}H NMR spectrum (400 MHz, 298 K) of $[Ru(IPr)_2(CO)(ZnMe)_2H_3][BAr^F_4]$ (**7**) in THF-d_8 and (top) after 6 days in solution at room temperature, showing small amounts of the high frequency resonances of $[IPrH][BAr^F_4]$.

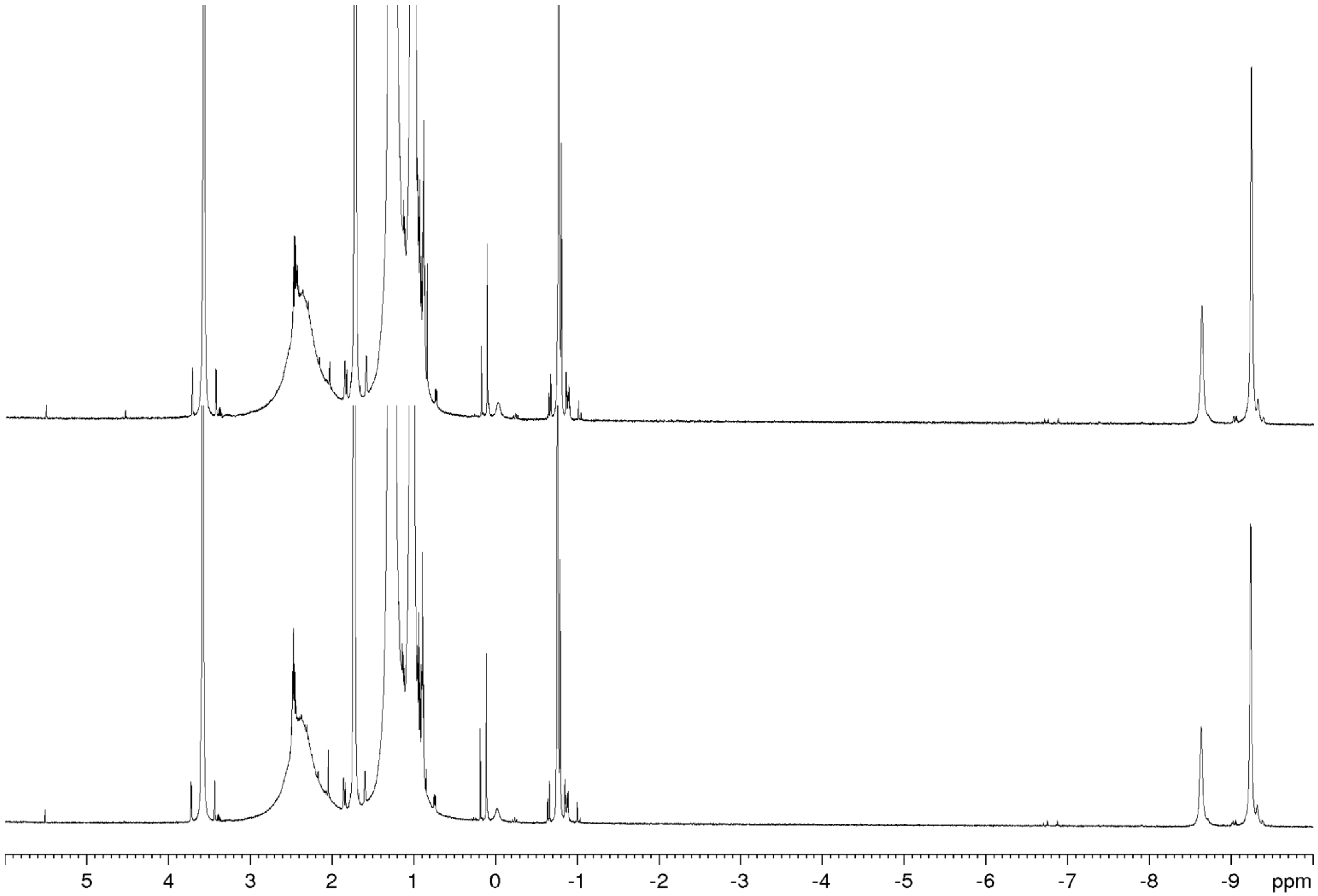

Figure S16. Low frequency region of the ^{1}H NMR spectrum (500 MHz, THF-d_8, 298 K) of [Ru(IPr)$_2$(CO)(ZnMe)$_2$H$_3$][BAr$^{F}_4$] (**7**) before (bottom) and (top) 6 h after addition of excess ZnH_2 showing the lack of any substitution reaction.

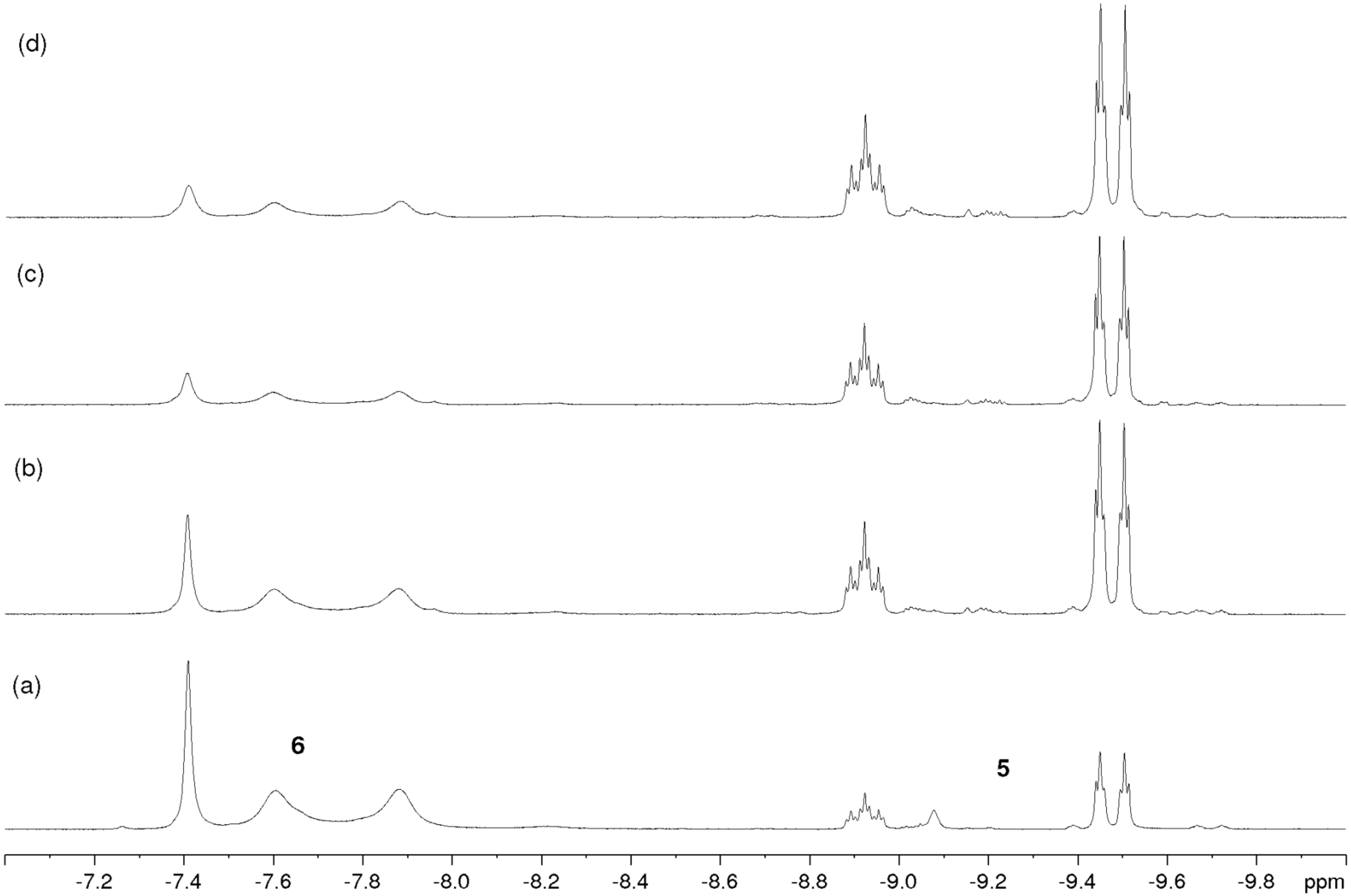

Figure S17. Low frequency RuH region of the ^{1}H NMR spectrum (500 MHz, THF-d_8, 298 K) recorded (a) 1, (b) 3, (c) 5.5 and (d) 7 h after mixing $[Ru(IPr)_2(CO)H][BAr^F{}_4]$ (**1**) with ZnH_2 (2 eq). Formation of $[Ru(IPr)_2(CO)(ZnH)H_3]$ (**6**) precedes conversion through to $[Ru(IPr)_2(CO)(ZnH)_2H_3][BAr^F{}_4]$ (**5**).

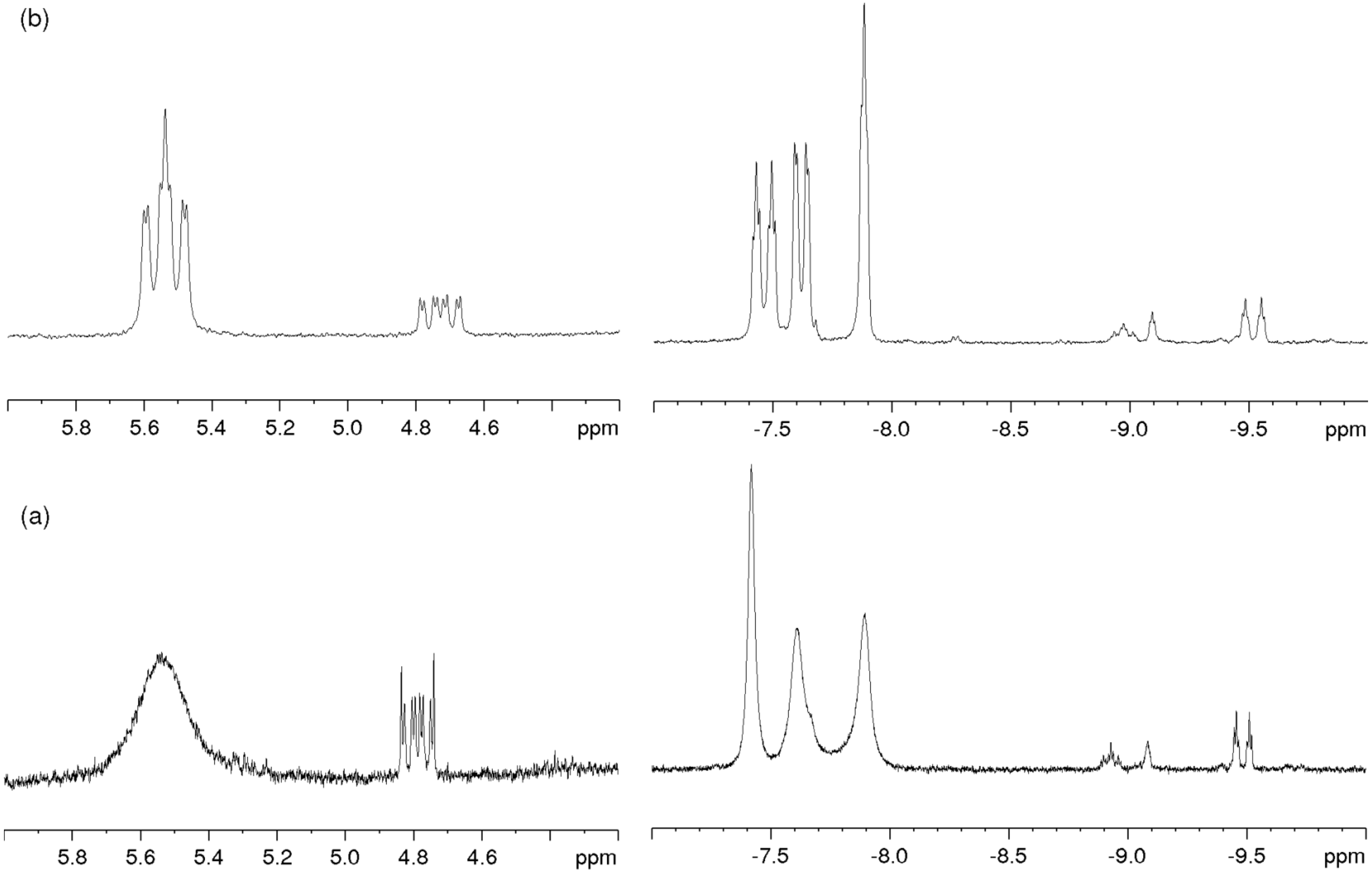

Figure S18. (a) ZnH (left) and RuH (right) regions of the 298 K ^{1}H NMR spectrum (500 MHz, THF-d_8) recorded 1 h after mixing [Ru(IPr)$_2$(CO)H][BAr$^F{}_4$] (**1**) with ZnH_2 (3 eq). The spectrum in (b) shows the result of then cooling the sample to 246 K (measured at 400 MHz) to highlight the sharpening of the signals of [Ru(IPr)$_2$(CO)(ZnH)H$_3$] (**6**) c.f. Figure S20.

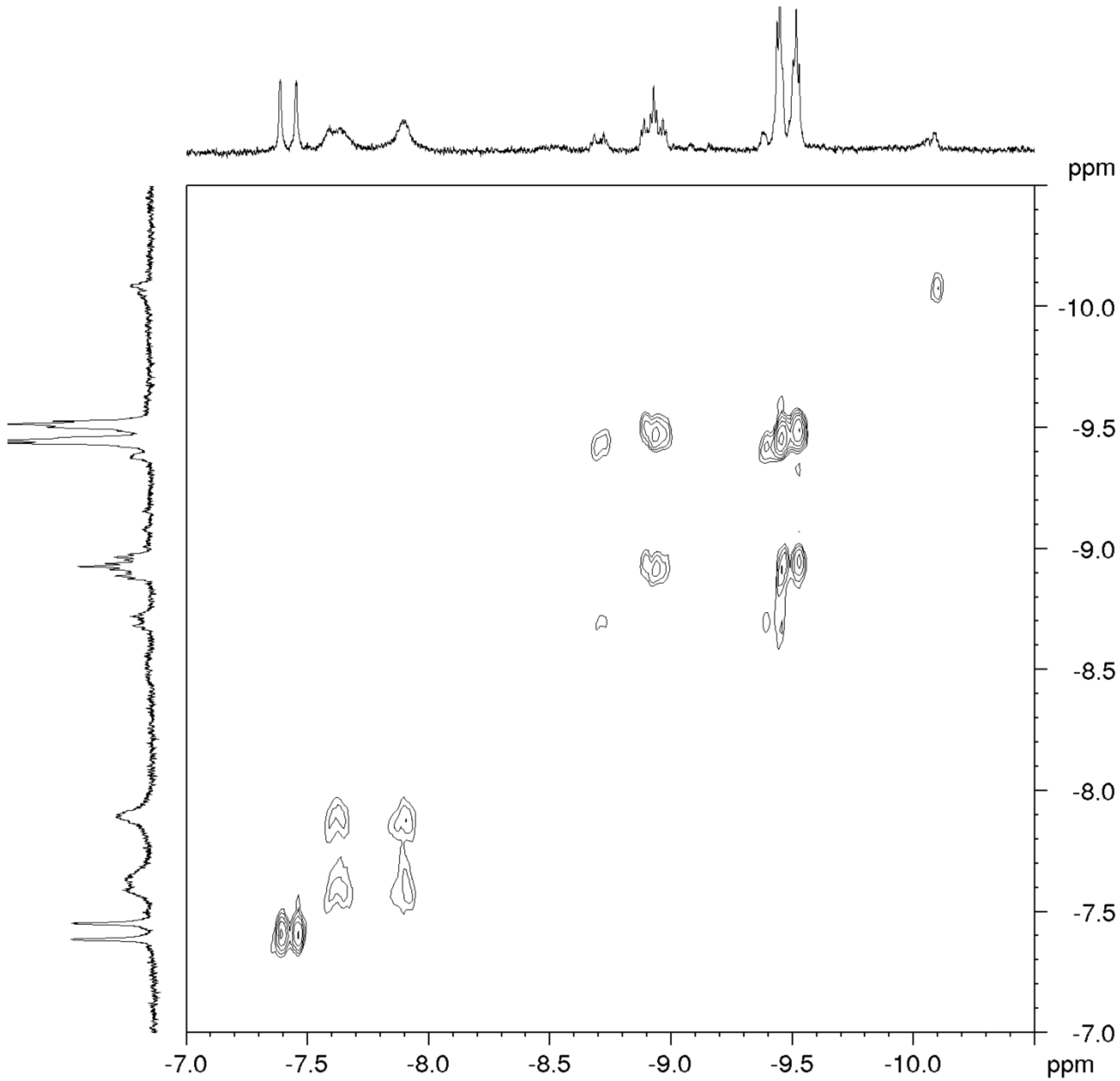

Figure S19. ^{1}H NOESY spectrum (400 MHz, 298 K) of a THF-d_8 sample containing both [Ru(IPr)$_2$(CO)(ZnH)$_2$H$_3$][BAr$^F{}_4$] (**5**) and [Ru(IPr)$_2$(CO)(ZnH)H$_3$] (**6**), to highlight the EXSY peaks between the two broad, lowest frequency RuH resonances of the latter.

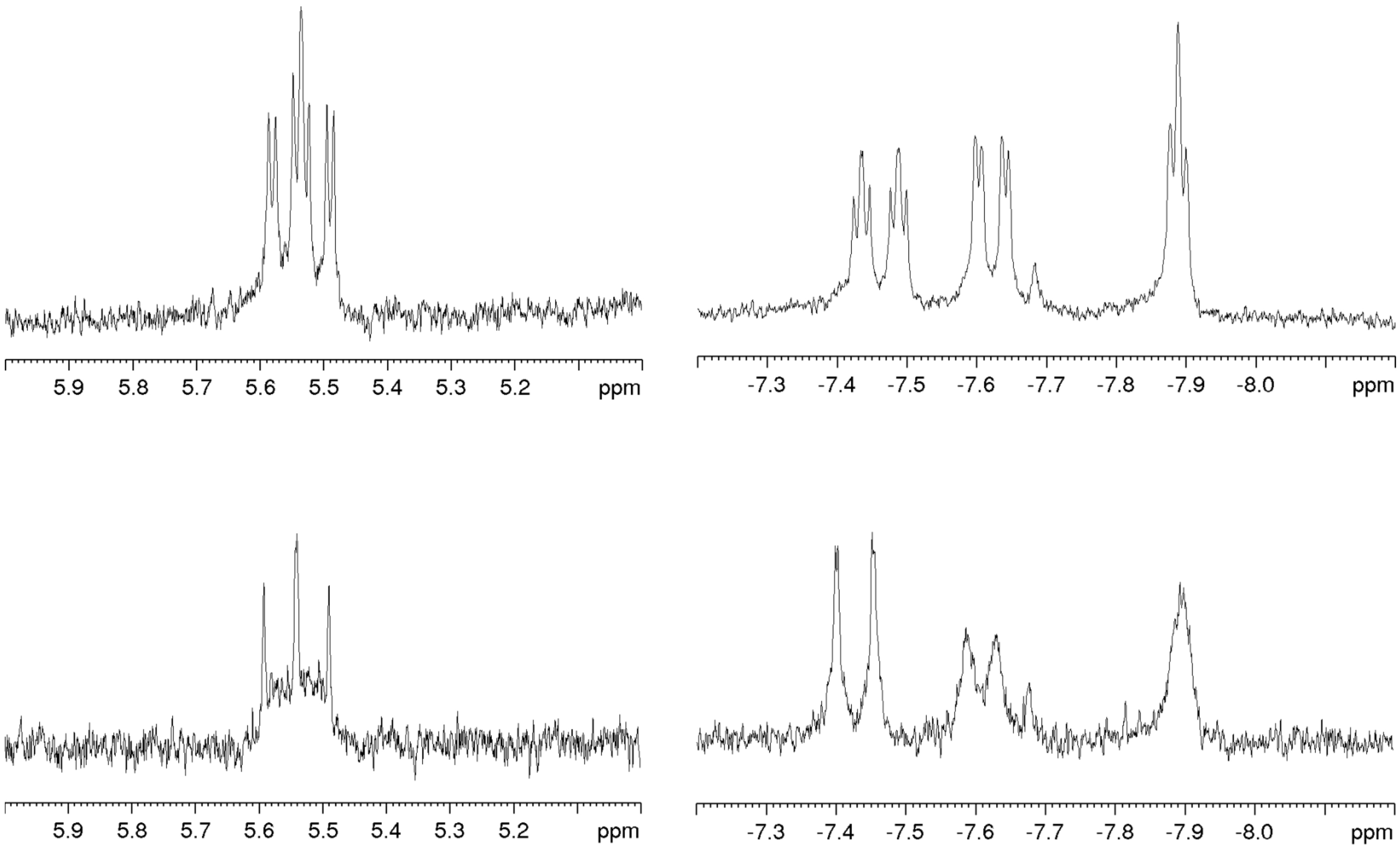

Figure S20. (Left) ZnH and (right) RuH regions of the (bottom) 290 K and (top) 238 K ^{1}H NMR spectrum (THF-d_8, 400 MHz) of redissolved crystalline $[Ru(IPr)_2(CO)(ZnH)H_3]$ (**6**).

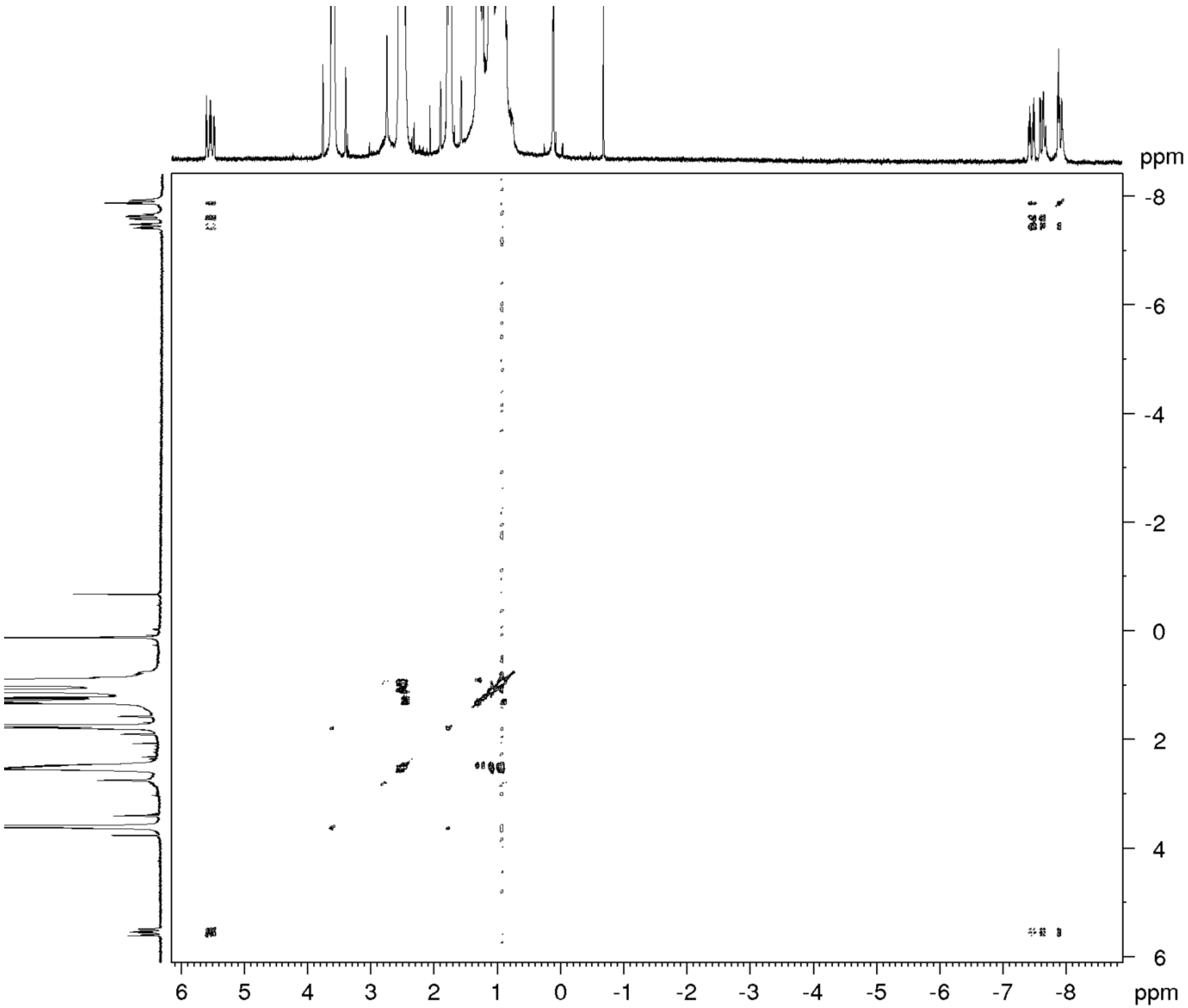

Figure S21. ^{1}H COSY spectrum (THF-d_8, 400 MHz, 246 K) of [Ru(IPr)$_2$(CO)(ZnH)H$_3$] (**6**) highlighting the correlation of the ZnH resonance at δ 5.6 to all three RuH resonances.

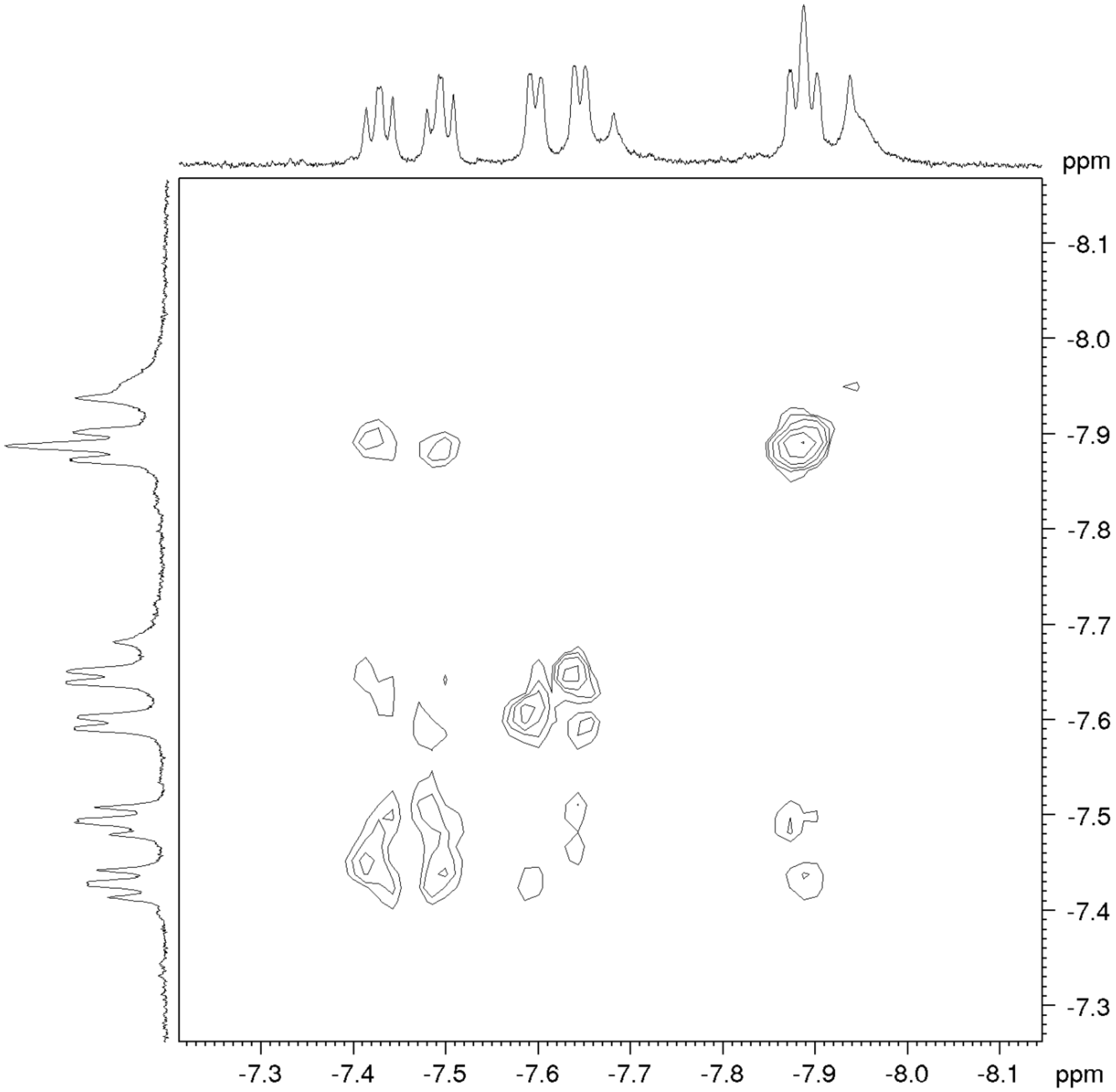

Figure S22. RuH region of the ^{1}H COSY spectrum (THF-d_8, 400 MHz, 246 K) of $[Ru(IPr)_2(CO)(ZnH)H_3]$ (**6**).

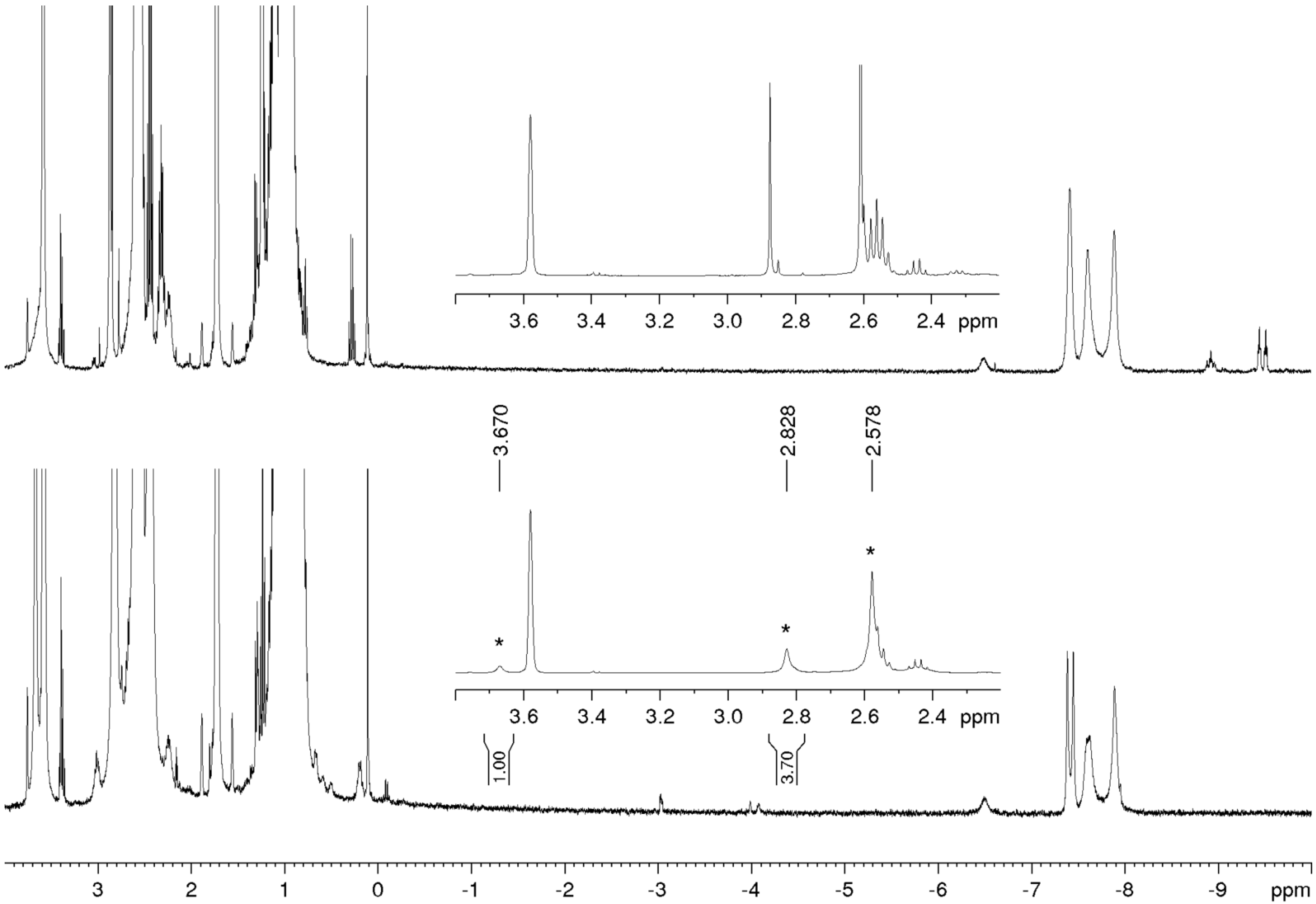

Figure S23. Low frequency region of the ${}^1H$ NMR spectrum (400 MHz, THF-d_8, 298 K) of the reaction of $[Ru(IPr)_2(CO)H][BAr^F{}_4]$ (**1**) with ZnH_2 (3 eq) in presence of 1.2 equiv TMEDA (bottom) 10 min after mixing (inset highlights signals (*) indicative of $[(tmeda)ZnH(THF)][BAr^F{}_4]$)[1] and (top) 20 h after mixing, illustrating the slowing down of $[Ru(IPr)_2(CO)(ZnH)H_3]$ (**6**) conversion to $[Ru(IPr)_2(CO)(ZnH)_2H_3][BAr^F{}_4]$ (**5**).

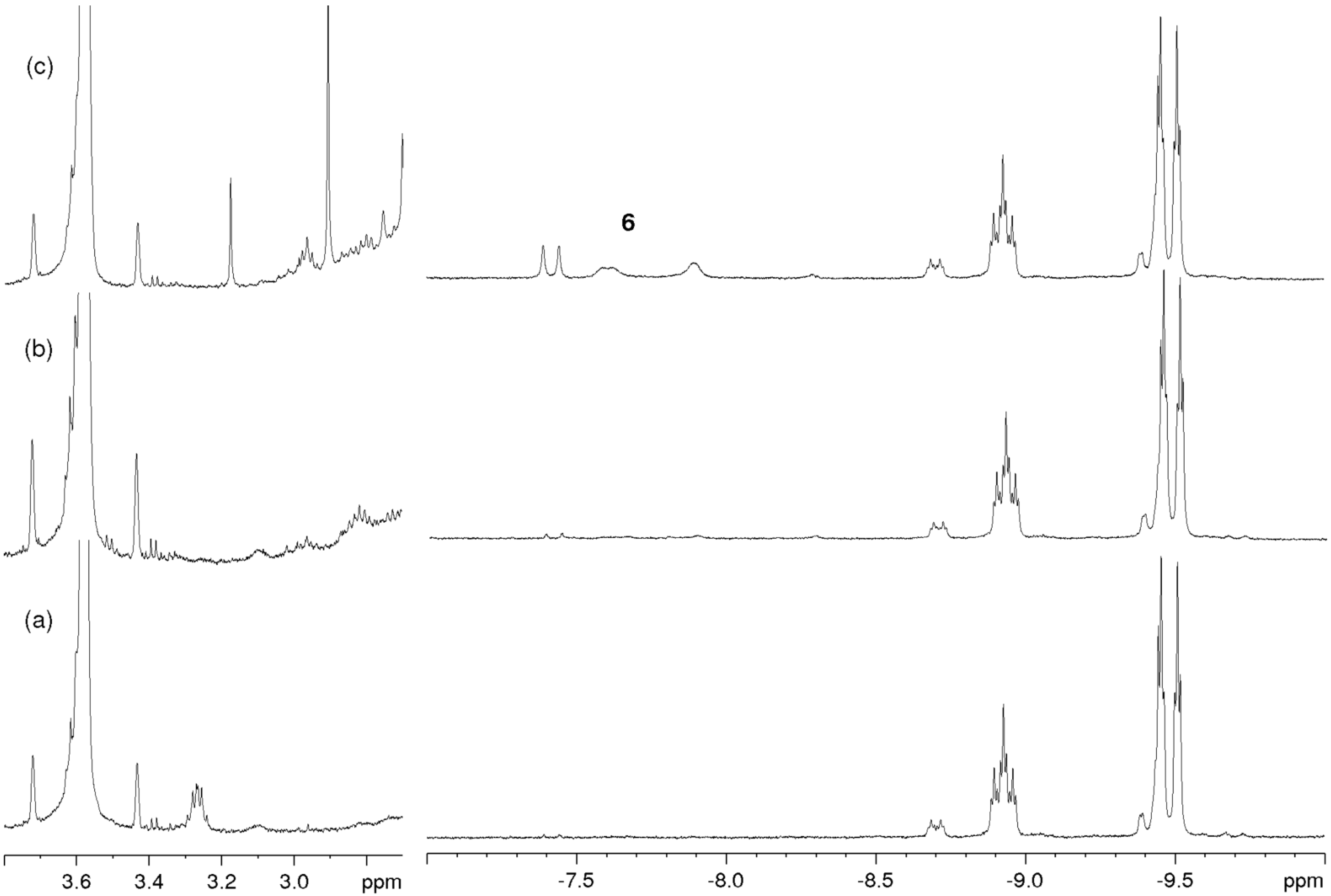

Figure S24. ^{1}H NMR spectrum (500 MHz, 298 K) of $[Ru(IPr)_2(CO)(ZnH)_2H_3][BAr^F{}_4]$ (**5**) in THF-d_8 (a) before and (b) 5 min after addition of 1.2 equiv TMEDA. Spectrum (c) was recorded 4 h after addition. Of note is the absence of any signals for $[(tmeda)ZnH(THF)][BAr^F{}_4]$[1] (see Figure S23 for details) and the absence of any impact on conversion to $[Ru(IPr)_2(CO)(ZnH)H_3]$ (**6**).

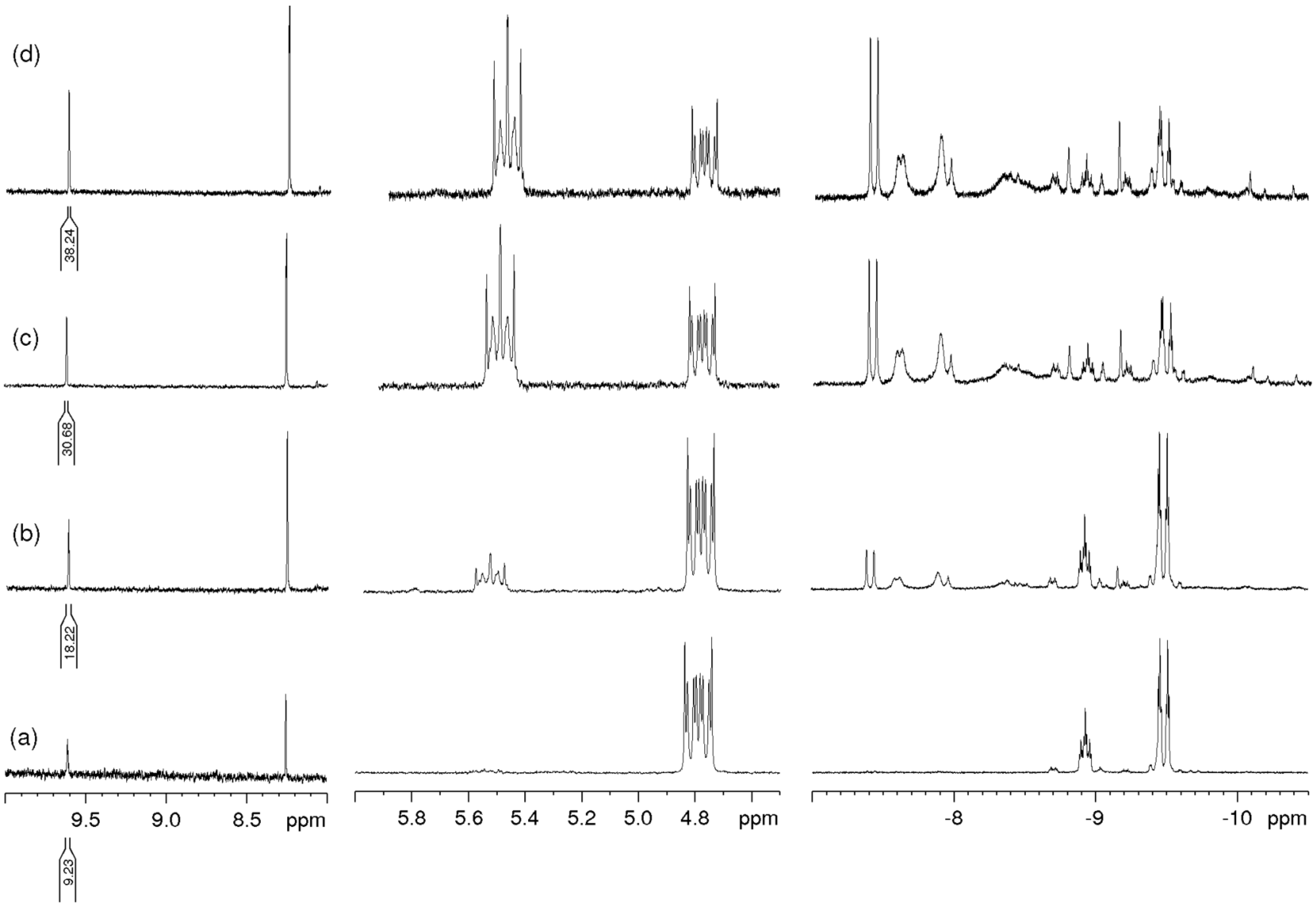

Figure S25. ^{1}H NMR spectrum (THF-d_8, 500 MHz, 298 K) of (a) $[Ru(IPr)_2(CO)(ZnH)_2H_3][BAr^F{}_4]$ (**5**) and appearance of resonances for $[Ru(IPr)_2(CO)(ZnH)H_3]$ (**6**) and $[IPrH]^+$ upon heating the sample at 60 °C for (b) 45 min and (c) 130 min. The sample was then left for 12 h at room temperature, whereupon (d) was recorded. The integration of the $[IPrH]^+$ resonance uses a Si grease impurity (integral = 100) as an internal reference.

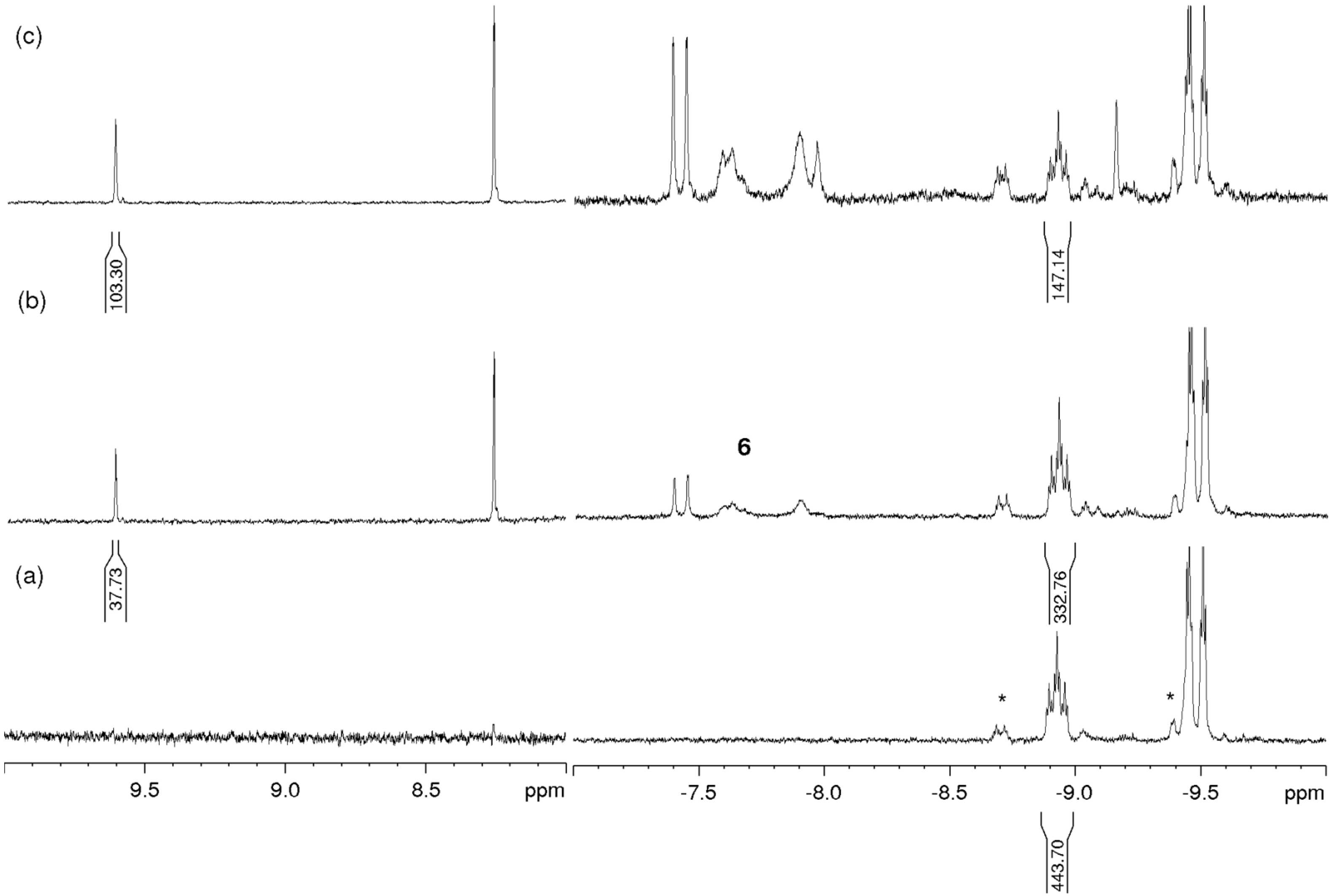

Figure S26. Partial ^{1}H NMR spectrum (500 MHz, 298 K) of (a) $[Ru(IPr)_2(CO)(ZnH)_2H_3][BAr^F{}_4]$ (**5**) in THF-d_8 and appearance of resonances for $[Ru(IPr)_2(CO)(ZnH)H_3]$ (**6**) and $[IPrH]^+$ over (b) 12 h and (c) 3 days at room temperature. Integrals relative to a Si grease impurity (integral set to 100) as internal reference. * designates an unknown Ru-H containing impurity sometimes found in batches of **5**.

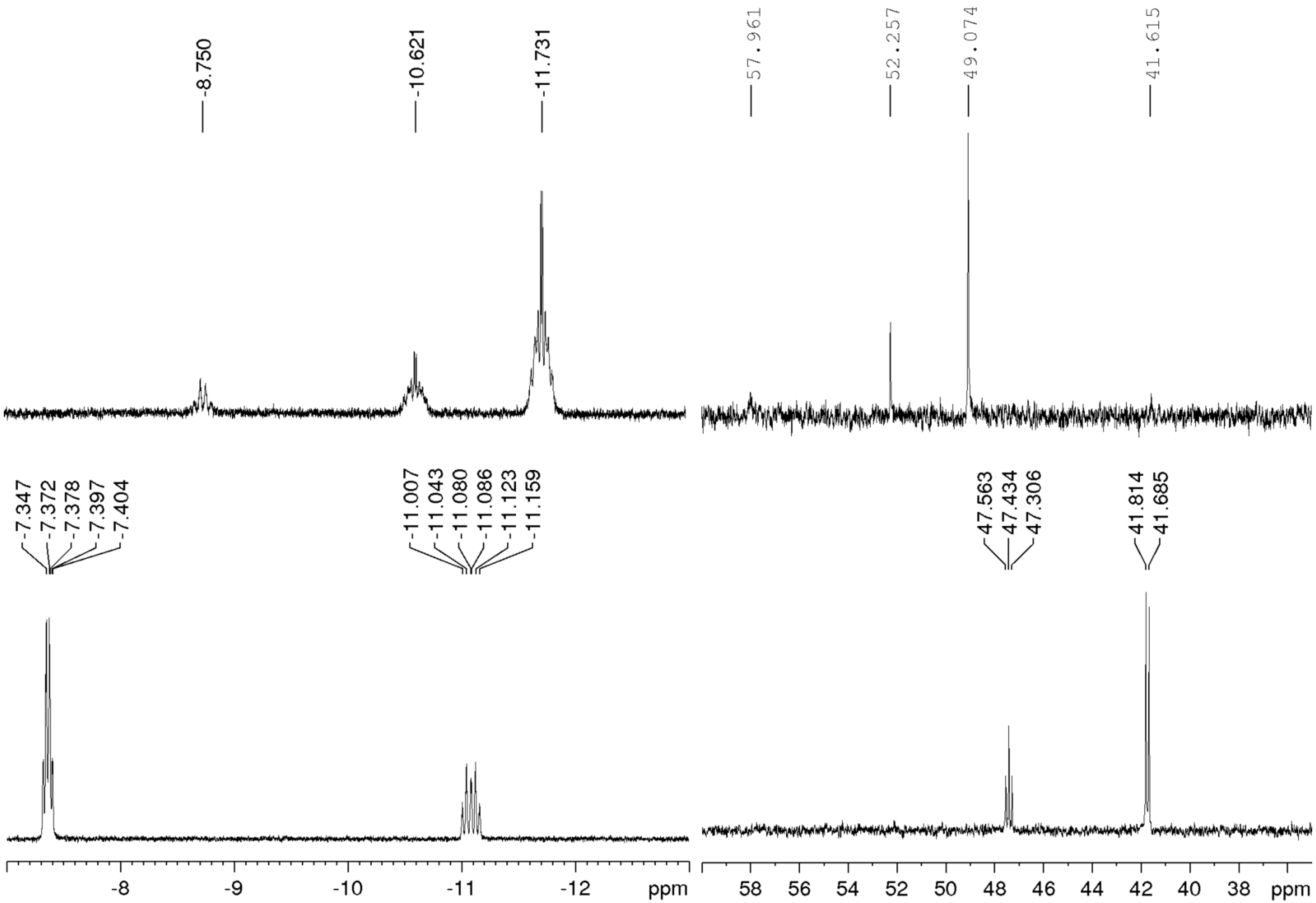

Figure S27. (Bottom, left) Hydride region of the ^{1}H NMR spectrum (500 MHz, 298 K) and (bottom, right) $^{31}P\{^{1}H\}$ NMR spectrum (202 MHz) of $[Ru(PPh_3)_3(ZnMe)_2H_3][BAr^F{}_4]$ in C_6D_6. Top left and right show the change in appearance[2] of the respective spectra upon evaporation of C_6D_6 and 10 min after redissolution of the resulting solid in THF-d_8.

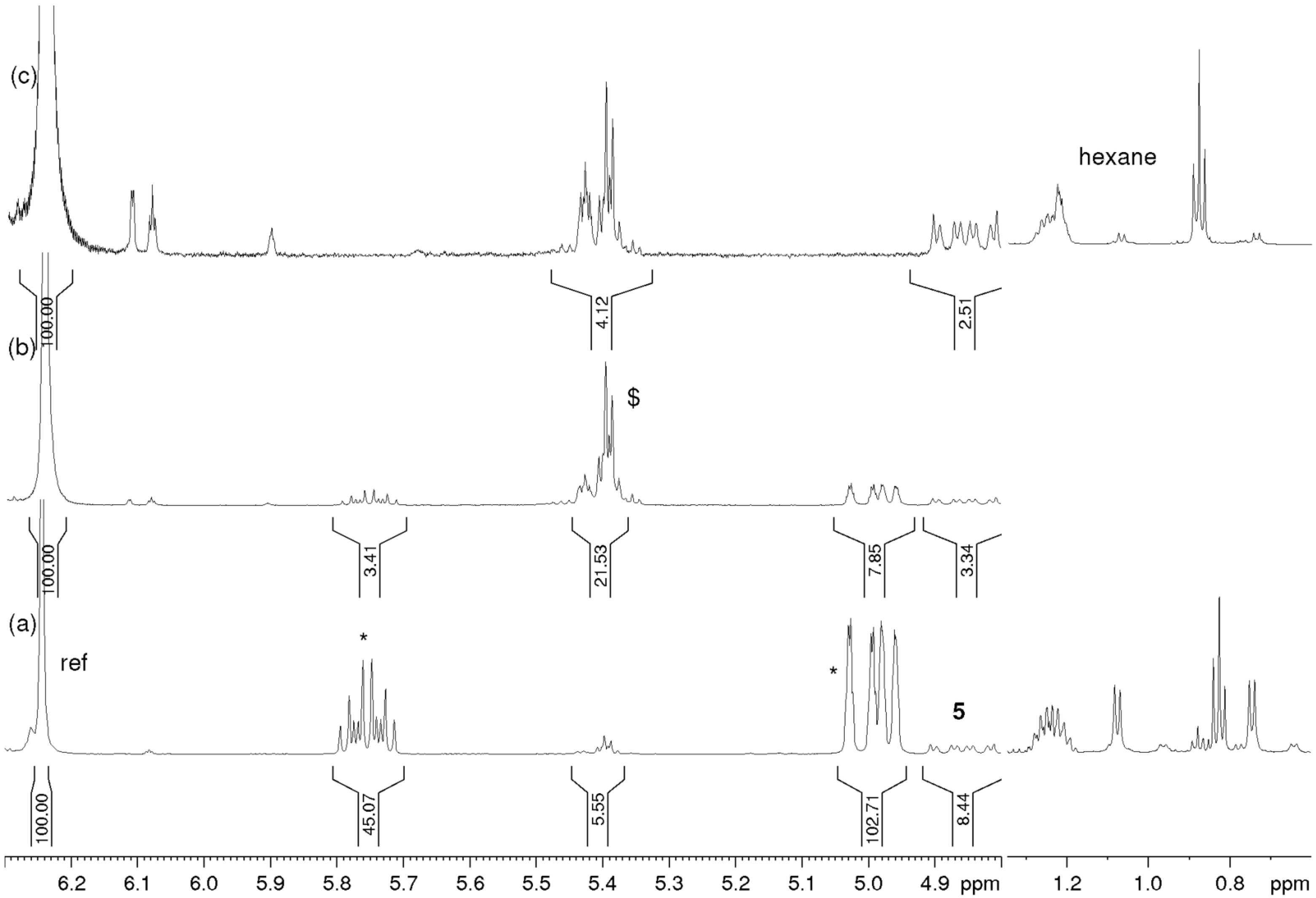

Figure S28. ^{1}H NMR spectrum (C_6D_6, 500 MHz, 298 K) of 1-hexene hydrogenation using $[Ru(IPr)_2(CO)(ZnH)_2H_3][BAr^F_4]$ (**5**). (a) Pre-addition of H_2, (b) after 3 h at room temperature and (c) 92 h at room temperature (ref = 1,3,5-$(MeO)_3C_6H_3$, integral set to 100; * = 1-hexene; $ = 2-hexene). Note the depletion of the integral for **5**, indicative of its limited solubility in benzene.

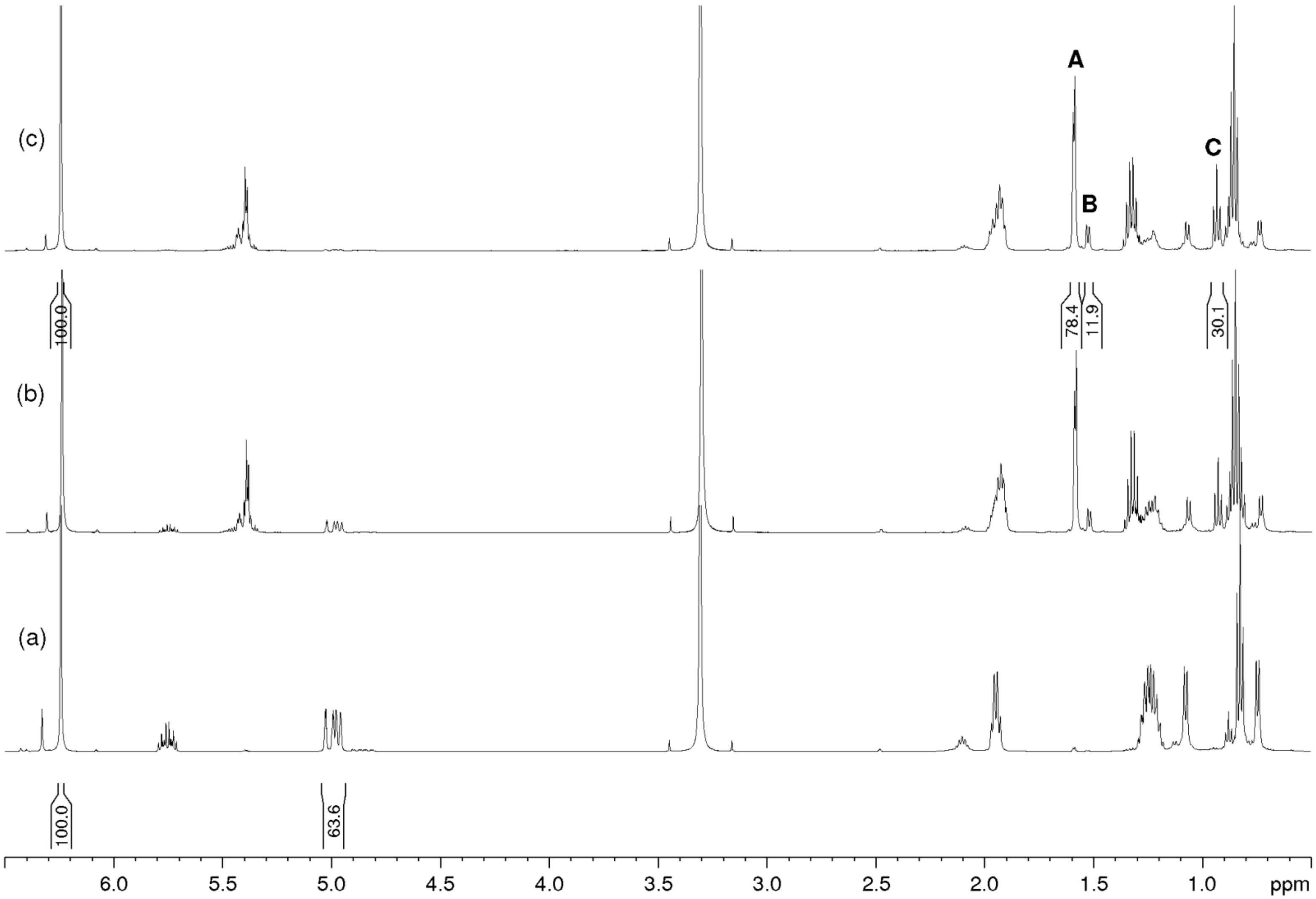

Figure S29. ^{1}H NMR spectrum (C_6D_6, 500 MHz, 298 K) of 1-hexene isomerization using $[Ru(IPr)_2(CO)(ZnH)_2H_3][BAr^F_4]$ (**5**) recorded (a) 15 min, (b) 18 h and (c) 42 h after sample preparation (1,3,5-$(MeO)_3C_6H_3$ reference, integral = 100; **A** = *trans*-2-hexene; **B** = *cis*-2-hexene; **C** = *trans*-3-hexene). Integrals demonstrate the full conversion of 1-hexene (2H) to *trans*-/*cis*-2-hexene (3H) and *trans*-3-hexene (6H).

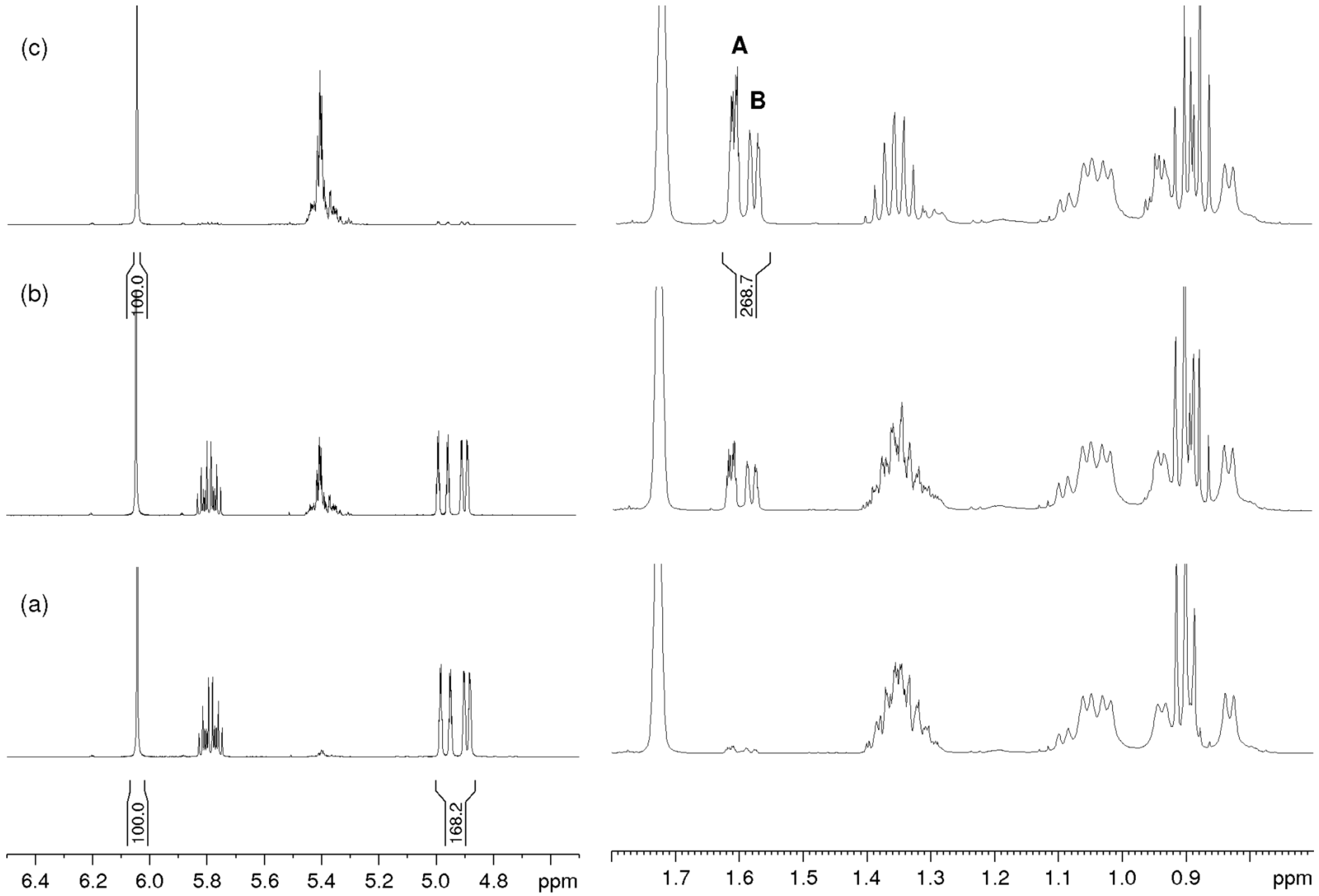

Figure S30. ^{1}H NMR spectrum (THF-d_8, 500 MHz, 298 K) of 1-hexene isomerization using $[Ru(IPr)_2(CO)H][BAr^F{}_4]$ (**1**) recorded (a) 10 min, (b) 3 h 15 min and (c) 18 h after sample preparation (1,3,5-$(MeO)_3C_6H_3$ reference, integral = 100; **A** = trans-2-hexene; **B** = cis-2-hexene). Integrals demonstrate the full conversion of 1-hexene (2H, δ 4.93) to *trans*-/*cis*-2-hexene (3H, ca δ 1.6).

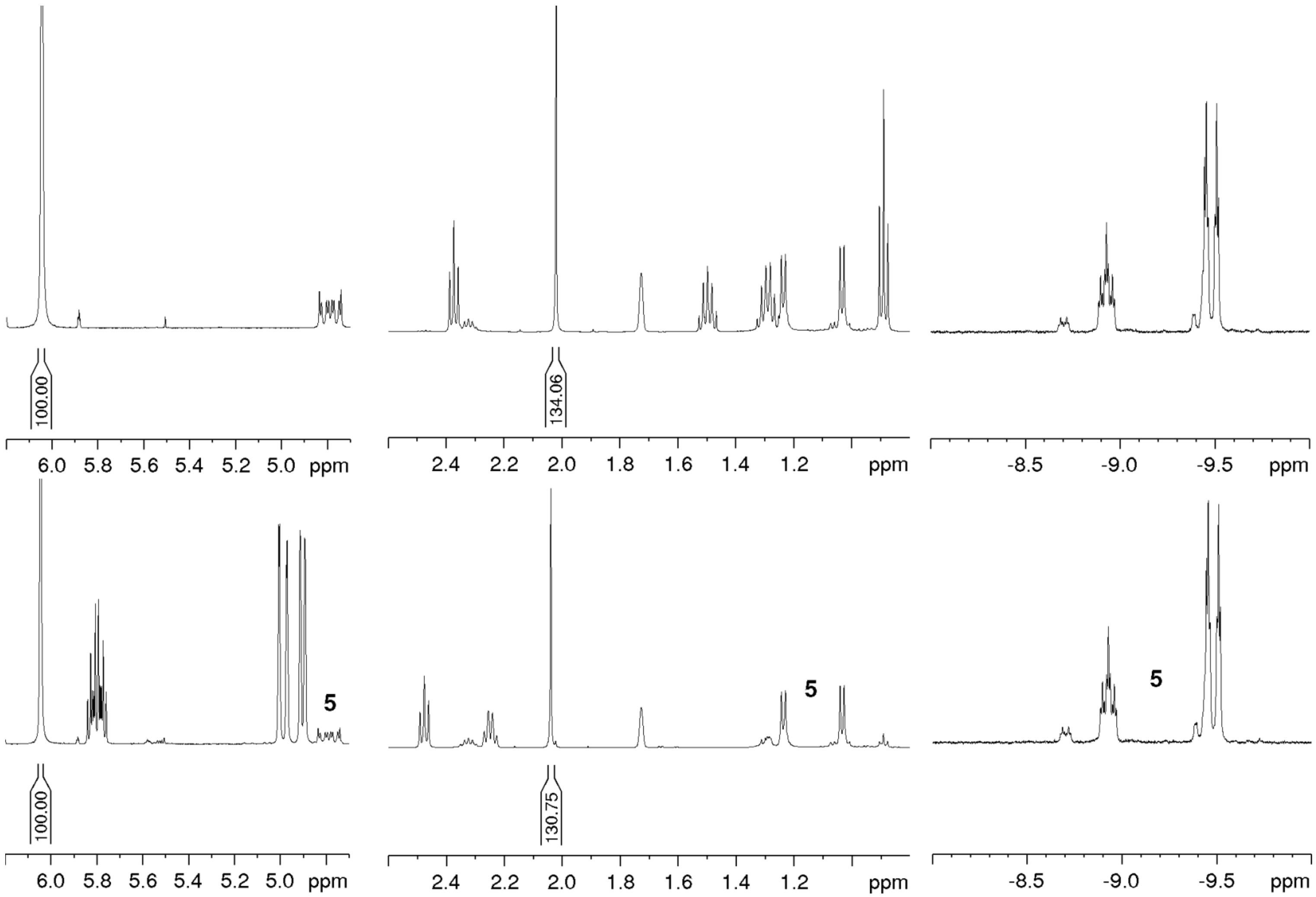

Figure S31. ^{1}H NMR spectrum (THF-d_8, 500 MHz, 298 K) of 5-hexene-2-one hydrogenation using $[Ru(IPr)_2(CO)(ZnH)_2H_3][BAr^F_4]$ (**5**) recorded (bottom) before and (top) 16 min after addition of H_2 (1,3,5-$(MeO)_3C_6H_3$ reference, integral = 100). Integrals shown full conversion of 5-hexene-2-one (δ 2.04) to hexanone (δ 2.02). Full retention of the diagnostic hydride signals of **5** at the end of the reaction are indicated in the traces at the far right of the stack plot.

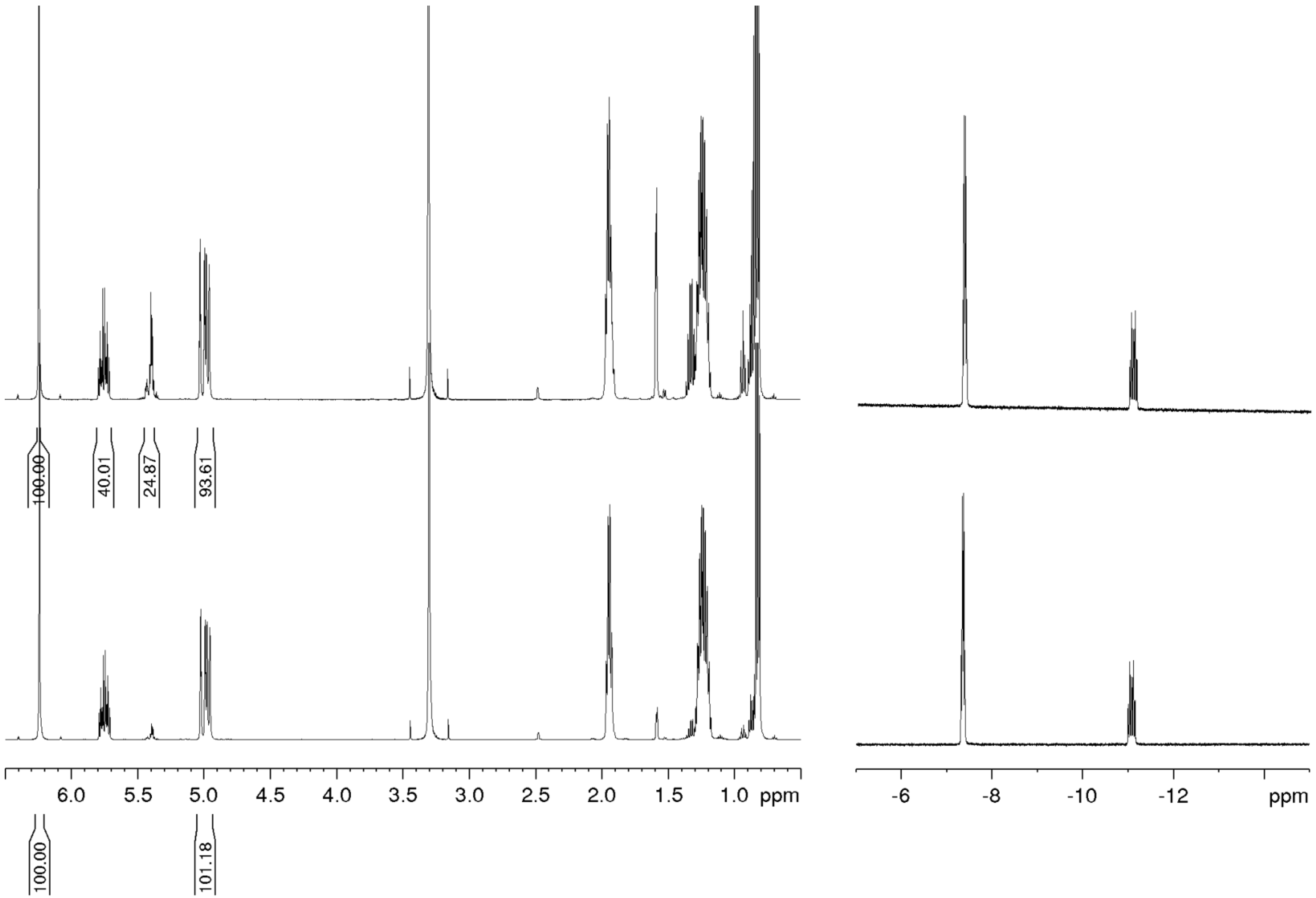

Figure S32. ^{1}H NMR spectrum (C_6D_6, 500 MHz, 298 K) of 1-hexene isomerization using $[Ru(PPh_3)_3(ZnMe)_2H_3][BAr^F{}_4]$ recorded (bottom) 10 min and (top) 90 h after sample preparation (1,3,5-$(MeO)_3C_6H_3$ reference, integral = 100) showing the low conversion to *trans*-/*cis*-2-hexene (δ 5.4).

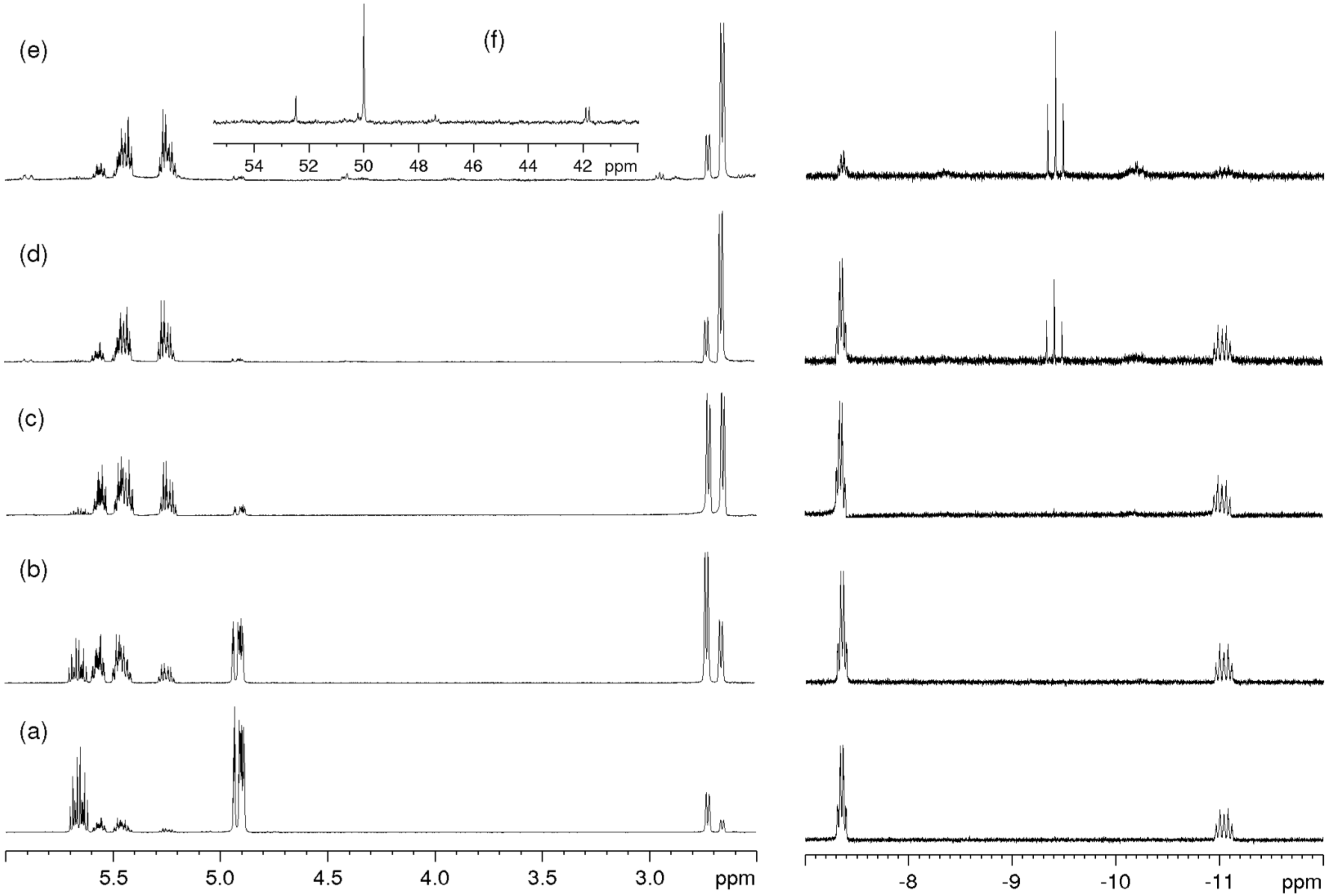

Figure S33. ^{1}H NMR spectrum (C_6D_6, 500 MHz, 298 K) of 5-hexene-2-one isomerization using $[Ru(PPh_3)_3(ZnMe)_2H_3][BAr^F_4]$. Spectra (a) 15 min, (b) 90 min, (c) 5 h, (d) 21 h and (e) 46 h after sample preparation. Formation of *trans*- and *cis*-4-hexene-2-one is supported by resonances at δ 2.7.[3] Degradation of the Ru salt with time gives resonances (δ –9.41, t, $^{2}J_{HP}$ = 37.5 Hz; $^{31}P\{^{1}H\}$ (spectrum f) δ 50.0 (s)) consistent with formation of $[(C_6D_6)Ru(PPh_3)_2H][BAr^F_4]$.[4]

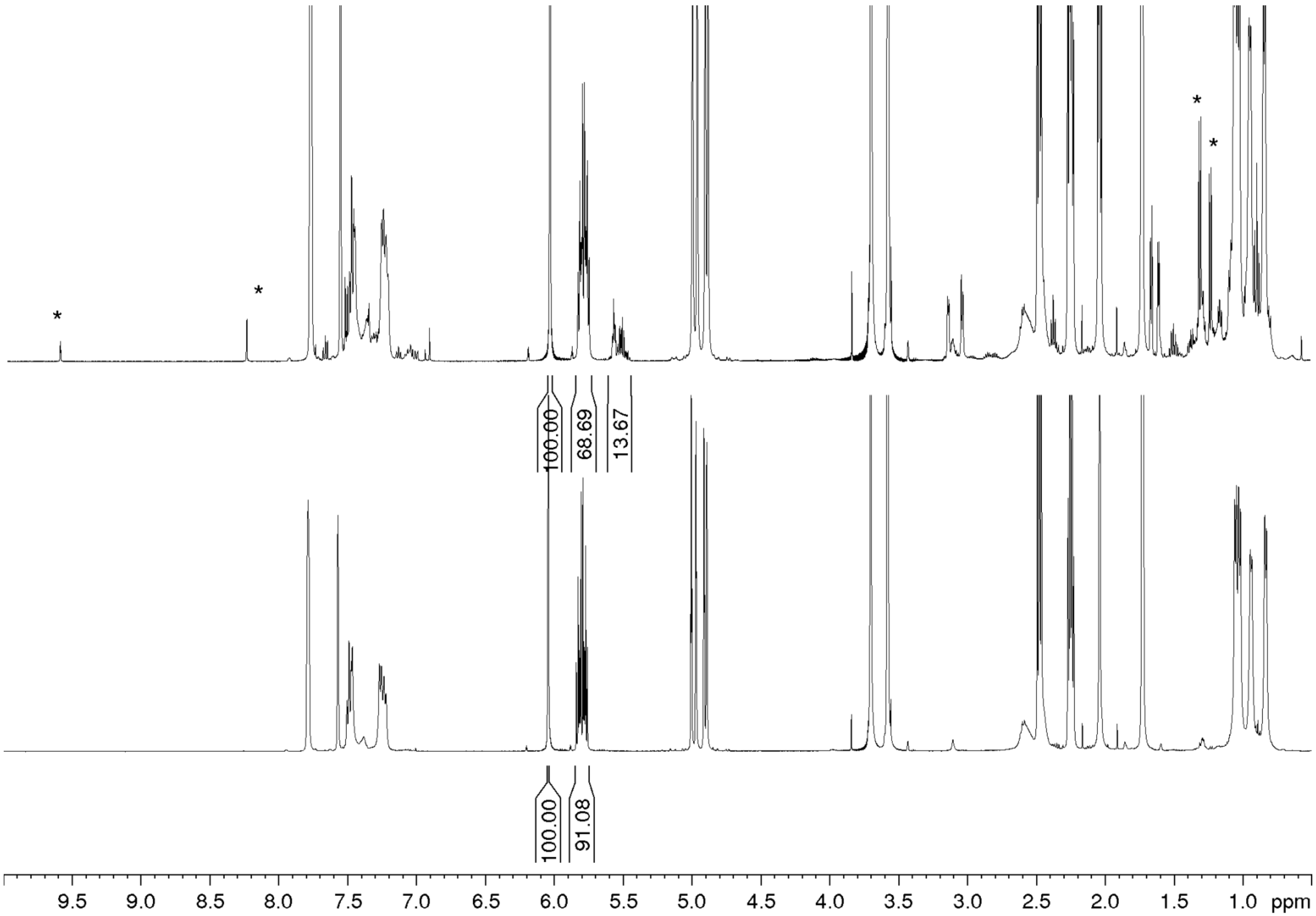

Figure S34. ^{1}H NMR spectrum (THF-d_8, 500 MHz, 298 K) of 5-hexene-2-one isomerization using [Ru(IPr)$_2$(CO)H][BAr$^{F}{}_4$] (**1**) at (a) 10 min and (b) 18 h after sample preparation. The resonances marked * match those of [IPrH][BAr$^{F}{}_4$], suggestive of **1** partially decomposing over the catalytic run.

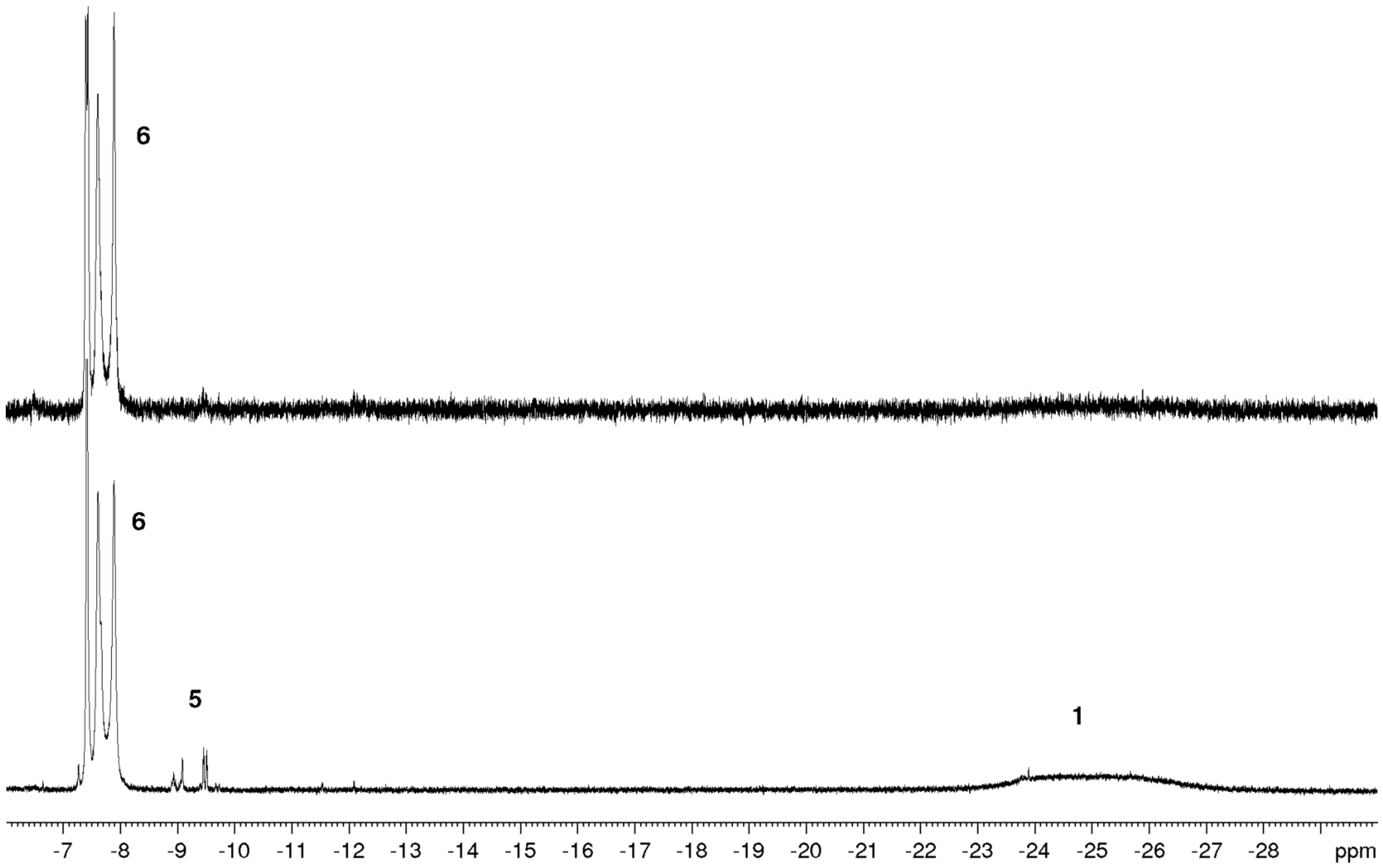

Figure S35. RuH region of the ^{1}H NMR spectrum (THF-d_8, 500 MHz, 298 K) recorded (bottom) 15 min after mixing [Ru(IPr)$_2$(CO)H][BAr$^F{}_4$] (**1**) and 1.8 equiv ZnH_2 and (top) 20 min after mixing [Ru(IPr)$_2$(CO)H][BAr$^F{}_4$] (**1**) and 9.6 equiv ZnH_2. The very broad Ru–H signal at ca. δ –25 is consistent with the presence of unreacted **1**.

S-2 Computational Studies

S 2-1. QTAIM Studies

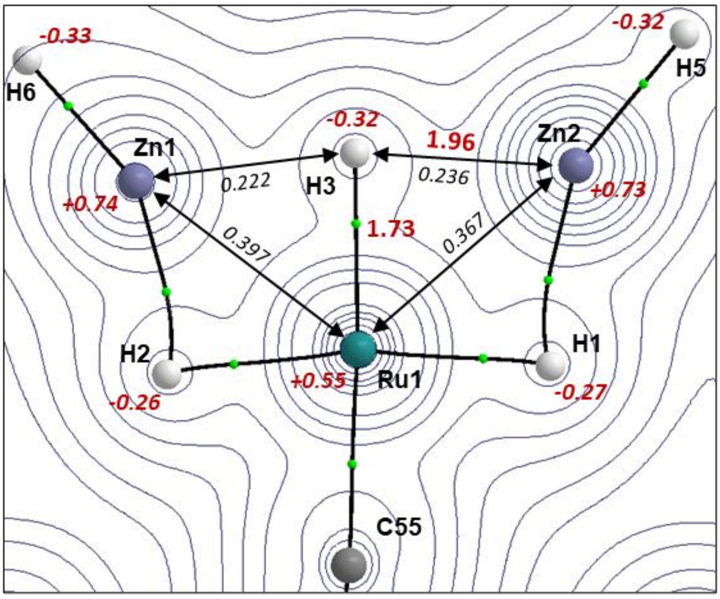

Bond Path	Distance (Å)	ρ(r)	$\nabla^2\rho$(r)	ε	H(r)	*d*(A,B)
**Ru–H1**	1.70	0.104	0.196	0.037	-0.040	0.646
**Ru–H2**	1.70	0.105	0.193	0.030	-0.040	0.667
**Ru–H3**	1.73	0.096	0.202	0.213	-0.035	0.628
**Zn2–H1**	1.79	0.069	0.123	0.391	-0.019	0.332
**Zn2–H5**	1.52	0.116	0.201	0.008	-0.043	0.820
**Zn1–H2**	1.76	0.072	0.129	0.363	-0.020	0.343
**Zn1–H8**	1.53	0.114	0.196	0.005	-0.041	0.822

Figure 36. QTAIM molecular graph with selected BCP metrics (au) tabulated for **5**$^+$**.** Density contours are shown in the {Zn1RuZn2} plane with axial ligands omitted for clarity. BCPs are shown as green spheres with selected atomic charges shown in italics. Delocalization indices for selected atom pairs that do not show a bond path are also shown.

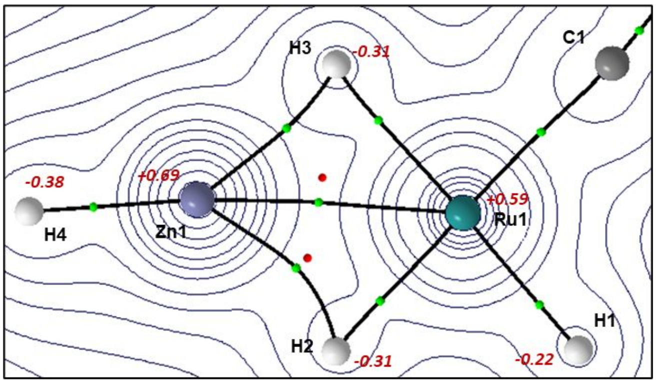

Bond Path	Distance (Å)	ρ(r)	$\nabla^2\rho$(r)	ε	H(r)	d(A,B)
Ru1–H1	1.63	0.129	0.119	0.048	-0.060	0.844
Ru1–H3	1.73	0.089	0.202	0.129	-0.030	0.583
Ru1–H2	1.77	0.098	0.179	0.127	-0.036	0.659
Ru1–Zn1	2.41	0.066	0.118	0.846	-0.020	0.465
Zn1–H3	1.76	0.075	0.126	0.361	-0.022	0.387
Zn1–H2	1.91	0.060	0.086	2.816	-0.016	0.299
Zn1–H4	1.55	0.109	0.195	0.010	-0.038	0.813

Figure S37. QTAIM molecular graph with selected BCP metrics (au) tabulated for **6**$^+$**.** Density contours are shown in the {Zn1Ru1H3} plane with axial ligands omitted for clarity. BCPs and RCPs are shown as green and red spheres respectively, with selected atomic charges shown in italics.

S2.2 Computed Reaction Profiles

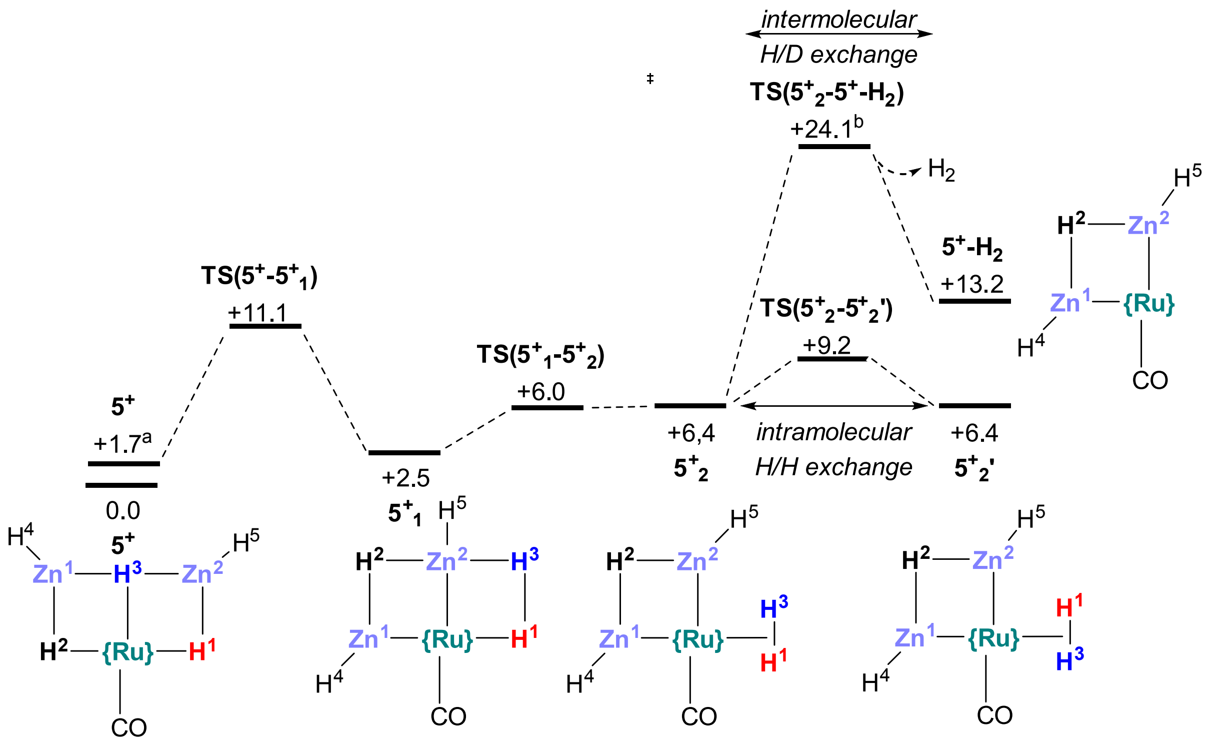

Figure S38. Computed free energy profiles (BP86-D3(PCM=THF)/Def2-TZVP)//BP86-/SDD(Ru,Zn), 6-31G**; kcal/mol) for intramolecular H-exchange and intermolecular H/D exchange in **5^+** (axial IPr ligands are omitted for clarity). [a]Conformation with a different orientation of the NHC ligands was located from the IRC characterization of **TS(5^+-5^+_1)**. We have previously shown[5] that interconversion between such conformations involves a series of low energy barriers and as such this was not considered further here.

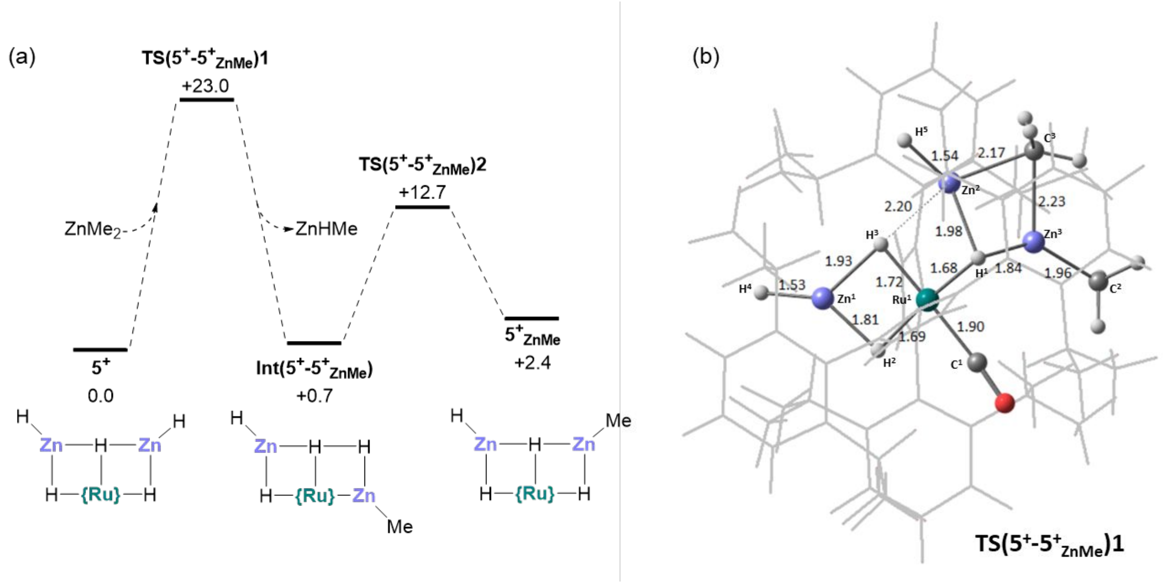

Figure S39. (a) Computed free energy profile for the first ZnH/ZnMe exchange in **5^+** (BP86-D3(PCM=THF)/Def2-TZVP)//BP86-/SDD(Ru,Zn), 6-31G**; kcal/mol; axial IPr ligands are omitted for clarity). (b) Computed geometry of **TS(5^+-5^+_{ZnMe})1** highlighting selected distances (Å); participating atoms in ball and stick mode, spectator NHC ligands depicted as wireframe.

S2.3 Computed Structures (A) and Energies (atomic units).

(i) Structures used in QTAIM Studies (Heavy atoms fixed from X-ray; H atoms optimised)

[5]$^{+}$

```
140
SCF =     -2985.8417406900
Ru -0.07623  0.04894  0.11501
H  -1.18355  1.28290 -0.26122
H   0.88703 -1.15524  0.82528
H   0.64399 -0.05114 -1.45704
Zn  1.85696 -1.36372 -0.66052
H   3.08643 -1.97437 -1.32014
Zn -0.90149  1.08499 -1.99120
H  -1.40023  1.32141 -3.41985
O  -1.26597  0.61294  2.85677
N  -1.24839 -2.59918 -1.14546
N  -2.80748 -1.53607 -0.13879
N   1.15510  2.92265  0.53830
N   2.42551  1.47437  1.47382
C  -1.46216 -1.46076 -0.40075
C  -2.40960 -3.32071 -1.32937
H  -2.41834 -4.24805 -1.89515
C  -3.38823 -2.66009 -0.70990
H  -4.44864 -2.86800 -0.60788
C  -3.60366 -0.66461  0.70487
C  -3.73382 -0.97157  2.05850
C  -4.55232 -0.14705  2.82690
H  -4.66862 -0.34973  3.89491
C  -5.21889  0.90992  2.25579
H  -5.85830  1.54388  2.88083
C  -5.09397  1.18017  0.92839
H  -5.64490  2.01902  0.49150
C  -4.30021  0.38268  0.09331
C  -4.24147  0.64609 -1.39396
H  -3.31790  0.17649 -1.78749
C  -5.42196 -0.02212 -2.10893
H  -5.45064 -1.11150 -1.94240
H  -5.36485  0.14915 -3.19776
H  -6.38091  0.39357 -1.75245
C  -4.18488  2.13274 -1.74845
H  -5.13428  2.64662 -1.51671
H  -4.00511  2.25732 -2.82920
H  -3.37997  2.65950 -1.20919
C  -3.04559 -2.17305  2.68235
H  -2.10614 -2.35993  2.12904
C  -3.93226 -3.42760  2.56501
H  -4.89401 -3.26684  3.08255
H  -3.43886 -4.29422  3.03810
H  -4.15342 -3.70113  1.52188
C  -2.69393 -1.98278  4.16510
H  -2.14680 -1.04764  4.35427
H  -2.06882 -2.82352  4.51000
H  -3.59809 -1.98371  4.79854
C   0.00672 -3.14896 -1.60748
C   0.66908 -4.03324 -0.75127
C   1.78886 -4.71105 -1.29220
H   2.33175 -5.41709 -0.65590
C   2.19248 -4.50211 -2.57705
H   3.05838 -5.04413 -2.97256
C   1.52188 -3.61456 -3.38317
H   1.86295 -3.45742 -4.41125
C   0.40146 -2.90354 -2.92734
C  -0.34126 -1.97981 -3.84284
H  -0.97385 -1.31859 -3.21978
C  -1.29729 -2.76209 -4.76799
H  -0.73531 -3.45434 -5.41925
H  -1.85791 -2.06755 -5.41634
H  -2.02954 -3.35712 -4.19800
C   0.58407 -1.08063 -4.65603
H   1.15953 -1.64720 -5.40864
H   1.30860 -0.55158 -4.01389
H  -0.00516 -0.32448 -5.20098
C   0.21039 -4.33318  0.65634
H  -0.49350 -3.53392  0.95345
C  -0.55012 -5.65294  0.69872
H   0.10129 -6.49643  0.40819
H  -1.41979 -5.65794  0.02074
H  -0.92089 -5.85708  1.71810
C   1.33199 -4.33668  1.67362
H   0.92137 -4.47623  2.68837
H   1.90582 -3.39499  1.66962
H   2.04698 -5.16146  1.50490
C   1.31547  1.57069  0.67176
C   2.10027  3.62988  1.25720
H   2.11405  4.71593  1.27121
C   2.90043  2.72589  1.83713
H   3.77552  2.84178  2.47006
C   3.21216  0.28385  1.71587
C   4.28094  0.04817  0.83850
C   5.02225 -1.12877  1.04344
H   5.85381 -1.35834  0.36940
C   4.72047 -1.98760  2.07856
H   5.30656 -2.90203  2.21944
C   3.70315 -1.67630  2.96701
H   3.50936 -2.34029  3.81504
C   2.92948 -0.52925  2.80906
C   1.89786 -0.14411  3.84593
H   1.20769  0.58107  3.38716
C   1.07172 -1.32808  4.35537
H   0.58764 -1.87432  3.52911
H   0.28329 -0.97208  5.03866
H   1.68724 -2.04646  4.92403
C   2.59504  0.56572  5.01403
H   3.31669 -0.10550  5.51189
H   1.85886  0.88887  5.77046
H   3.14983  1.46008  4.68290
```

C	4.74508	1.06423	-0.21068
H	3.99821	1.87557	-0.25852
C	4.89423	0.50395	-1.59988
H	5.62969	-0.31919	-1.64216
H	5.25472	1.28839	-2.28872
H	3.94938	0.10417	-2.00106
C	6.05634	1.67751	0.25401
H	5.97854	2.12589	1.25946
H	6.38694	2.46902	-0.44119
H	6.85931	0.92084	0.29708
C	0.22587	3.58783	-0.34159
C	0.55806	3.66448	-1.70724
C	-0.33388	4.31710	-2.53968
H	-0.12167	4.38667	-3.61038
C	-1.49124	4.90527	-2.03303
H	-2.17867	5.42415	-2.70866
C	-1.74907	4.84755	-0.69086
H	-2.64985	5.33512	-0.30065
C	-0.91561	4.18705	0.19568
C	-1.22873	4.15761	1.67034
H	-0.52204	3.46928	2.16558
C	-1.03304	5.55649	2.27473
H	-0.00648	5.93316	2.12675
H	-1.23428	5.54419	3.35970
H	-1.72150	6.28769	1.81641
C	-2.64460	3.63881	1.93467
H	-3.41138	4.32907	1.54146
H	-2.82104	3.54175	3.01933
H	-2.81344	2.65265	1.47646
C	1.90127	3.16058	-2.23846
H	2.21204	2.29904	-1.62111
C	1.85519	2.68416	-3.69877
H	1.70435	3.52277	-4.40079
H	1.05222	1.94977	-3.87991
H	2.81589	2.21327	-3.96591
C	2.95540	4.25395	-2.10660
H	3.93393	3.89773	-2.47336
H	3.09314	4.58917	-1.06539
H	2.67776	5.13822	-2.70710
C	-0.84440	0.33553	1.83776

[6⁺]

138

SCF = -2758.1512274400

Ru	0.05557	0.08389	0.06934
H	-0.51515	-1.40127	0.41412
H	0.58388	1.72638	-0.30798
N	-2.85398	0.07315	1.23650
N	-2.44373	1.97500	0.36387
N	3.16258	-0.39263	-0.20768
N	2.08393	-1.91127	-1.26851
C	-1.85132	0.74756	0.56058
C	-3.98154	0.85325	1.43196
H	-4.85939	0.46664	1.94100
C	-3.72498	2.04504	0.88739
H	-4.31649	2.95344	0.82553
C	-2.92891	-1.32322	1.60029
C	-2.62790	-1.69385	2.90979
C	-2.89845	-3.01818	3.27166
H	-2.67346	-3.34264	4.29347
C	-3.45378	-3.90202	2.38735
H	-3.67129	-4.92917	2.70176
C	-3.73090	-3.50781	1.10265
H	-4.16548	-4.22306	0.39485
C	-3.46807	-2.19685	0.66082
C	-2.07477	-0.71771	3.92299
H	-1.69083	0.15637	3.37261
C	-3.18076	-0.23208	4.85906
H	-3.99639	0.26739	4.30930
H	-2.78397	0.48972	5.59502
H	-3.62489	-1.07331	5.42134
C	-0.90263	-1.30705	4.71334
H	-1.23213	-2.10070	5.40805
H	-0.41678	-0.52330	5.31803
H	-0.14548	-1.73832	4.04107
C	-3.80161	-1.76752	-0.74589
H	-3.42380	-0.74095	-0.88364
C	-5.31879	-1.73918	-0.96644
H	-5.76108	-2.74496	-0.85010
H	-5.55828	-1.38802	-1.98559
H	-5.82524	-1.06925	-0.25049
C	-3.10224	-2.63137	-1.79537
H	-2.01018	-2.60060	-1.66395
H	-3.33793	-2.26429	-2.80976
H	-3.42675	-3.68606	-1.74487
C	-1.92410	3.10829	-0.36319
C	-1.12632	4.03148	0.32117
C	-0.76888	5.19604	-0.35688
H	-0.14842	5.94587	0.14328
C	-1.19529	5.40619	-1.65139
H	-0.90504	6.32729	-2.17085
C	-1.97911	4.48683	-2.30354
H	-2.30232	4.67991	-3.33169
C	-2.37525	3.30026	-1.67109
C	-0.70511	3.81792	1.75120
H	-0.81957	2.74782	1.98192
C	-1.61513	4.59798	2.69876
H	-1.54523	5.68554	2.51420
H	-1.32909	4.41816	3.75003
H	-2.67504	4.31070	2.58682
C	0.75546	4.17620	2.00447
H	1.42878	3.62468	1.33052
H	1.03369	3.91172	3.03812
H	0.94747	5.25752	1.88113
C	-3.27519	2.30143	-2.37178
H	-3.25892	1.36373	-1.79005
C	-4.72313	2.79133	-2.44027
H	-5.15097	2.97507	-1.44064
H	-5.36188	2.04612	-2.94558
H	-4.79428	3.73455	-3.01117
C	-2.77780	1.97266	-3.78384
H	-2.85318	2.84448	-4.45791
H	-3.39146	1.16834	-4.22572
H	-1.72508	1.64494	-3.78584

C	1.86855	-0.80499	-0.47921
C	4.10145	-1.19965	-0.81574
H	5.16728	-1.02777	-0.69584
C	3.43293	-2.14587	-1.47709
H	3.77816	-2.98499	-2.07393
C	3.54132	0.64322	0.71787
C	3.77416	1.93397	0.23275
C	4.15665	2.90198	1.16226
H	4.34964	3.92367	0.81950
C	4.29557	2.60018	2.49873
H	4.58349	3.38510	3.20723
C	4.09222	1.31153	2.94736
H	4.23039	1.07522	4.00751
C	3.73007	0.29191	2.06184
C	3.70845	2.25545	-1.24897
H	3.07511	1.49473	-1.74042
C	3.10122	3.62507	-1.52872
H	2.10786	3.73548	-1.06371
H	2.97673	3.77106	-2.61517
H	3.74460	4.44506	-1.16297
C	5.11713	2.17049	-1.85398
H	5.79108	2.90428	-1.37714
H	5.09184	2.38797	-2.93649
H	5.56665	1.17188	-1.72322
C	3.65646	-1.14991	2.53589
H	3.15379	-1.74080	1.75073
C	2.87572	-1.33756	3.82040
H	3.37957	-0.85427	4.67776
H	2.80636	-2.41267	4.06416
H	1.85982	-0.92958	3.76564
C	5.03214	-1.71247	2.74921
H	5.66099	-1.66549	1.84198
H	4.98263	-2.77124	3.05994
H	5.56963	-1.16125	3.54238
C	1.12131	-2.86924	-1.75705
C	0.69966	-3.87742	-0.88063
C	-0.08100	-4.89916	-1.43245
H	-0.43015	-5.70785	-0.78219
C	-0.41625	-4.90600	-2.76098
H	-1.02082	-5.72828	-3.16218
C	-0.01337	-3.89081	-3.59434
H	-0.29967	-3.90147	-4.65102
C	0.76835	-2.83973	-3.10431
C	1.05826	-3.86879	0.59535
H	1.27585	-2.82173	0.86493
C	-0.10220	-4.30447	1.47641
H	-1.00370	-3.71265	1.26655
H	0.15703	-4.16182	2.53971
H	-0.35035	-5.37438	1.34920
C	2.28942	-4.71347	0.88887
H	2.11581	-5.77602	0.63665
H	2.54617	-4.66674	1.96226
H	3.17399	-4.37873	0.31949
C	1.22422	-1.73505	-4.04848
H	1.65388	-0.91851	-3.44081
C	0.04811	-1.14733	-4.83632
H	-0.38333	-1.88214	-5.53908
H	0.38061	-0.27755	-5.42777
H	-0.75451	-0.80859	-4.16158
C	2.31051	-2.24199	-5.00274
H	3.19695	-2.61421	-4.46159
H	2.64480	-1.43528	-5.67867
H	1.93286	-3.06960	-5.62953
Zn	0.38264	1.28519	-1.99618
O	0.77519	0.90186	2.95700
C	0.53873	0.51113	1.87766
H	-0.52680	-0.31659	-1.50569
H	0.71030	1.83401	-3.40767

(ii) Reactivity Studies: Intramolecular H/H Exchange

[5⁺] (0.0 kcal/mol)
140
SCF = -2985.86881596
H(0 K)= -2984.711207
G(298 K)= -2984.821217
SCF(CORR) = -6091.99713827
Low Freq. = 15.9269cm-1, 22.3851cm-1

Ru	-0.07065	0.05203	0.13150
H	-0.90700	1.46954	-0.29802
H	0.65554	-1.29119	0.88346
H	0.60196	-0.25030	-1.44098
Zn	1.57968	-1.75832	-0.56812
H	2.66981	-2.63239	-1.17461
Zn	-0.73040	1.14802	-2.03155
H	-1.16213	1.47506	-3.46156
O	-1.06185	0.88420	2.90211
N	-1.73504	-2.37295	-1.09841
N	-3.09134	-0.98947	-0.11660
N	1.69982	2.68176	0.43829
N	2.76787	1.01213	1.32712
C	-1.73548	-1.18606	-0.36798
C	-3.02804	-2.86437	-1.29800
H	-3.20987	-3.78214	-1.84915
C	-3.87576	-1.99636	-0.68343
H	-4.95737	-1.99308	-0.59000
C	-3.74328	0.02946	0.69507
C	-4.00412	-0.26595	2.06114
C	-4.72623	0.69083	2.80066
H	-4.93765	0.50014	3.85672
C	-5.19225	1.86994	2.20802
H	-5.75683	2.59457	2.80354
C	-4.95861	2.11249	0.84938
H	-5.35649	3.02118	0.38796
C	-4.24013	1.19531	0.05685
C	-4.11146	1.43050	-1.44815
H	-3.29535	0.78360	-1.82649
C	-5.40762	1.00109	-2.17938
H	-5.65039	-0.05960	-2.00263
H	-5.30188	1.14883	-3.26768

```
H  -6.26565  1.60396 -1.83505
C  -3.75793  2.88771 -1.80749
H  -4.58810  3.57693 -1.57543
H  -3.55688  2.97099 -2.88837
H  -2.86532  3.24367 -1.26554
C  -3.61727 -1.59722  2.71296
H  -2.78885 -2.03493  2.12578
C  -4.81125 -2.58484  2.66502
H  -5.67127 -2.17813  3.22452
H  -4.53204 -3.54739  3.12679
H  -5.14586 -2.78921  1.63545
C  -3.13099 -1.45414  4.17139
H  -2.32118 -0.71557  4.26547
H  -2.75985 -2.42721  4.53504
H  -3.95023 -1.15381  4.84710
C  -0.61532 -3.18852 -1.53659
C  -0.15898 -4.21968 -0.66259
C   0.79450 -5.12432 -1.17139
H   1.16252 -5.92841 -0.52706
C   1.26313 -5.02486 -2.48599
H   1.99808 -5.74478 -2.85960
C   0.79114 -4.00978 -3.32619
H   1.15633 -3.94999 -4.35586
C  -0.16549 -3.07534 -2.88210
C  -0.73059 -2.04222 -3.85556
H  -1.27615 -1.28144 -3.26664
C  -1.74887 -2.69900 -4.81934
H  -1.26334 -3.47725 -5.43319
H  -2.17196 -1.94272 -5.50192
H  -2.58426 -3.17108 -4.27621
C   0.37146 -1.31118 -4.65086
H   0.89608 -1.98989 -5.34481
H   1.12679 -0.86403 -3.98273
H  -0.07455 -0.50357 -5.25444
C  -0.71753 -4.41893  0.74766
H  -1.28115 -3.50976  1.02191
C  -1.70167 -5.61404  0.77366
H  -1.18900 -6.55273  0.50125
H  -2.53931 -5.47131  0.07121
H  -2.12411 -5.74126  1.78479
C   0.39230 -4.61059  1.80373
H  -0.05512 -4.67446  2.81002
H   1.11114 -3.77383  1.80153
H   0.95966 -5.54278  1.64024
C   1.62209  1.30114  0.58893
C   2.81696  3.21456  1.08464
H   3.02604  4.28003  1.07707
C   3.48247  2.16851  1.64581
H   4.39594  2.12866  2.23114
C   3.36704 -0.28486  1.61310
C   4.40307 -0.74085  0.74792
C   5.01575 -1.96949  1.06845
H   5.80853 -2.35369  0.41984
C   4.63576 -2.69951  2.19924
H   5.12510 -3.65267  2.42417
C   3.65478 -2.19571  3.06352
H   3.40125 -2.74994  3.97241
C   3.01226 -0.96918  2.80529
C   2.06673 -0.36817  3.84283
H   1.50691  0.44856  3.35892
C   1.04025 -1.38118  4.38884
H   0.46072 -1.84787  3.57554
H   0.33456 -0.87227  5.06594
H   1.52537 -2.18556  4.96816
C   2.88414  0.25398  5.00265
H   3.47463 -0.51666  5.52771
H   2.20950  0.72529  5.73761
H   3.58574  1.02495  4.64202
C   4.94511  0.08492 -0.42481
H   4.21588  0.88368 -0.65108
C   5.15599 -0.73230 -1.71815
H   5.95116 -1.48818 -1.59851
H   5.47087 -0.06057 -2.53475
H   4.23839 -1.25732 -2.02600
C   6.27753  0.76480 -0.01828
H   6.16720  1.40599  0.87132
H   6.65613  1.39139 -0.84371
H   7.04603  0.00654  0.21060
C   0.86470  3.55241 -0.37129
C   1.17342  3.67947 -1.75610
C   0.39464  4.58366 -2.50835
H   0.59685  4.70228 -3.57657
C  -0.61290  5.34950 -1.90838
H  -1.19641  6.05167 -2.51231
C  -0.86025  5.23362 -0.53526
H  -1.63135  5.85661 -0.07157
C  -0.12909  4.33548  0.26869
C  -0.37921  4.28287  1.77585
H   0.12413  3.38451  2.17304
C   0.24136  5.51933  2.47186
H   1.32659  5.59700  2.29162
H   0.08233  5.46478  3.56217
H  -0.22459  6.45146  2.10794
C  -1.87780  4.16797  2.12610
H  -2.43180  5.08207  1.85022
H  -1.99836  4.02883  3.21324
H  -2.35457  3.31400  1.61933
C   2.37896  2.98353 -2.39860
H   2.60141  2.07553 -1.80876
C   2.14290  2.54224 -3.85831
H   2.04612  3.40583 -4.53869
H   1.23413  1.92690 -3.96773
H   3.00441  1.95109 -4.21122
C   3.61985  3.91008 -2.32897
H   4.49737  3.41000 -2.77333
H   3.87464  4.18476 -1.29276
H   3.43876  4.84163 -2.89248
C  -0.72891  0.51191  1.84370
```

[5⁺] from IRC (+1.7 kcal/mol)

```
140
SCF  =          -2985.86716478
H(0 K)=         -2984.709933
G(298 K)=       -2984.821260
```

```
SCF(CORR) =      -6091.99280808
Low Freq. = 12.6161cm-1,
18.7938cm-1

Ru -0.00011  0.18502  0.00002
H   0.35943  0.38406 -1.65449
H  -0.35995  0.38343  1.65460
H   0.00091 -1.54623  0.00006
Zn  0.05877 -1.31335  2.01906
H   0.43036 -2.24907  3.16897
Zn -0.05694 -1.31339 -2.01888
H  -0.42721 -2.24931 -3.16907
O  -0.00201  3.24894  0.00031
N  -2.99445 -0.93222  0.13486
N  -2.96381  0.90246 -1.02968
N   2.99562 -0.92866 -0.13496
N   2.96269  0.90587  1.02975
C  -2.12105  0.06424 -0.30548
C  -4.29965 -0.71331 -0.31380
H  -5.10900 -1.39176 -0.06270
C  -4.27860  0.43409 -1.04305
H  -5.06674  0.97140 -1.56152
C  -2.68602  2.20555 -1.61880
C  -2.98387  3.35968 -0.84613
C  -2.79391  4.61282 -1.45983
H  -3.00738  5.52308 -0.89134
C  -2.34359  4.71479 -2.78052
H  -2.20155  5.70049 -3.23522
C  -2.09763  3.55811 -3.53034
H  -1.77771  3.65103 -4.57210
C  -2.27972  2.27598 -2.97588
C  -2.13681  1.03241 -3.85258
H  -1.92148  0.17188 -3.18998
C  -3.46537  0.72639 -4.58796
H  -4.29862  0.55950 -3.88604
H  -3.36049 -0.17916 -5.20981
H  -3.74386  1.56486 -5.24947
C  -0.98432  1.13801 -4.87038
H  -0.04347  1.45088 -4.38902
H  -1.21087  1.86254 -5.67146
H  -0.82116  0.16173 -5.35707
C  -3.55538  3.29213  0.57293
H  -3.40960  2.26442  0.95210
C  -5.07796  3.57702  0.55085
H  -5.27864  4.59474  0.17400
H  -5.49911  3.50460  1.56820
H  -5.62434  2.86900 -0.09378
C  -2.85555  4.25202  1.56055
H  -1.76683  4.09393  1.58612
H  -3.25603  4.09705  2.57696
H  -3.03857  5.30885  1.30119
C  -2.75771 -2.03138  1.05696
C  -2.92227 -1.78332  2.45154
C  -2.81274 -2.88726  3.32332
H  -2.92765 -2.73001  4.39937
C  -2.58017 -4.17807  2.83719
H  -2.50346 -5.01869  3.53398
C  -2.46973 -4.39926  1.45904
H  -2.32645 -5.41850  1.08797
C  -2.57048 -3.34065  0.53380
C  -2.58736 -3.64186 -0.96538
H  -2.40441 -2.69596 -1.50875
C  -3.98278 -4.16339 -1.39319
H  -4.22038 -5.11023 -0.87807
H  -4.00139 -4.35310 -2.47971
H  -4.78650 -3.44557 -1.16225
C  -1.50032 -4.64455 -1.40155
H  -1.67452 -5.64844 -0.97734
H  -0.49032 -4.31754 -1.10543
H  -1.50753 -4.74606 -2.49925
C  -3.32607 -0.41390  3.00587
H  -3.02050  0.35404  2.27274
C  -4.86508 -0.33513  3.16900
H  -5.22025 -1.10158  3.87933
H  -5.39244 -0.48692  2.21372
H  -5.15684  0.65327  3.56297
C  -2.64265 -0.07746  4.34685
H  -2.85045  0.97018  4.62133
H  -1.54945 -0.20881  4.29800
H  -3.01962 -0.70708  5.17108
C   2.12099  0.06669  0.30543
C   4.30053 -0.70821  0.31380
H   5.11071 -1.38565  0.06267
C   4.27805  0.43911  1.04314
H   5.06553  0.97734  1.56167
C   2.68327  2.20858  1.61892
C   2.27685  2.27843  2.97599
C   2.09315  3.56031  3.53050
H   1.77309  3.65279  4.57226
C   2.33770  4.71732  2.78073
H   2.19441  5.70283  3.23546
C   2.78816  4.61598  1.46004
H   3.00050  5.52653  0.89161
C   2.97969  3.36310  0.84629
C   3.55126  3.29634 -0.57279
H   3.40715  2.26835 -0.95185
C   2.84978  4.25498 -1.56046
H   1.76136  4.09487 -1.58618
H   3.25069  4.10081 -2.57682
H   3.03078  5.31213 -1.30099
C   5.07336  3.58371 -0.55081
H   5.27245  4.60183 -0.17420
H   5.49460  3.51175 -1.56815
H   5.62089  2.87670  0.09396
C   2.13542  1.03462  3.85259
H   1.92106  0.17390  3.18992
C   0.98283  1.13878  4.87044
H   1.20861  1.86342  5.67163
H   0.82077  0.16224  5.35697
H   0.04163  1.45069  4.38915
C   3.46436  0.73008  4.58789
H   4.29777  0.56420  3.88593
H   3.36055 -0.17563  5.20969
H   3.74190  1.56883  5.24945
```

C 2.76026 -2.02806 -1.05712
C 2.57472 -3.33761 -0.53405
C 2.47529 -4.39628 -1.45937
H 2.33332 -5.41573 -1.08837
C 2.58536 -4.17484 -2.83750
H 2.50968 -5.01552 -3.53434
C 2.81625 -2.88370 -3.32356
H 2.93089 -2.72623 -4.39960
C 2.92446 -1.77970 -2.45169
C 3.32657 -0.40975 -3.00593
H 3.02001 0.35778 -2.27279
C 4.86550 -0.32908 -3.16894
H 5.39298 -0.48032 -2.21364
H 5.15610 0.65971 -3.56280
H 5.22166 -1.09504 -3.87931
C 2.64283 -0.07408 -4.34694
H 3.02063 -0.70317 -5.17119
H 2.84937 0.97384 -4.62134
H 1.54979 -0.20677 -4.29816
C 2.59200 -3.63892 0.96511
H 2.40801 -2.69327 1.50856
C 1.50609 -4.64286 1.40119
H 1.68132 -5.64648 0.97675
H 0.49571 -4.31687 1.10525
H 1.51353 -4.74459 2.49888
C 3.98801 -4.15894 1.39284
H 4.00685 -4.34868 2.47935
H 4.79094 -3.44024 1.16192
H 4.22663 -5.10550 0.87768
C -0.00128 2.08129 0.00013

TS[5^{+}-5^{+}_{1}] (+11.0 kcal/mol)
140
SCF = -2985.84896647
H(0 K)= -2984.691401
G(298 K)= -2984.801518
SCF(CORR) = -6091.97938584
Low Freq. = -90.6895cm-1,
12.5543cm-1

Ru -0.07045 0.02838 0.00066
H -0.80649 -1.40072 -0.66780
H 0.60743 1.04260 1.08092
H -0.98985 -0.55328 1.33073
Zn 0.19471 -1.46319 2.07001
H 0.58530 -2.43332 3.18165
Zn -1.32930 -0.66871 -2.11555
H -2.02791 -0.54270 -3.47243
O 1.33395 1.62703 -2.19823
N -3.04415 0.92334 0.67419
N -1.99723 2.55611 -0.31065
N 1.95629 -2.41719 -0.51731
N 3.03199 -0.84540 0.55076
C -1.80785 1.24544 0.10956
C -3.93882 1.99332 0.59808
H -4.94859 1.91882 0.99000
C -3.28281 3.01456 -0.01684
H -3.59583 4.02277 -0.27034
C -1.03711 3.47209 -0.90550
C -0.18222 4.20148 -0.03840
C 0.68114 5.14457 -0.62877
H 1.35519 5.72416 0.00930
C 0.67087 5.37390 -2.01019
H 1.34324 6.12052 -2.44520
C -0.21193 4.66585 -2.83379
H -0.22312 4.86631 -3.90933
C -1.08977 3.69983 -2.30510
C -2.07113 2.97725 -3.22988
H -2.45986 2.09201 -2.69236
C -3.27715 3.88611 -3.57252
H -3.82408 4.20895 -2.67132
H -3.98759 3.35279 -4.22701
H -2.94252 4.79405 -4.10349
C -1.40512 2.47112 -4.52786
H -0.50568 1.87039 -4.31847
H -1.10879 3.30569 -5.18628
H -2.11353 1.84313 -5.09345
C -0.24460 4.06822 1.48329
H -0.75718 3.11956 1.71994
C -1.07987 5.22615 2.08339
H -0.61081 6.20169 1.86735
H -1.15362 5.12183 3.17964
H -2.10394 5.24934 1.67430
C 1.14845 4.00484 2.14002
H 1.75788 3.19101 1.71625
H 1.04338 3.82821 3.22413
H 1.70497 4.95048 2.02052
C -3.44338 -0.29131 1.36578
C -3.28670 -0.34210 2.77826
C -3.73822 -1.50189 3.43940
H -3.63173 -1.57428 4.52584
C -4.33789 -2.55313 2.73500
H -4.68706 -3.44131 3.27128
C -4.51240 -2.45902 1.34921
H -5.00998 -3.27287 0.81348
C -4.07851 -1.32590 0.63207
C -4.37597 -1.21159 -0.86239
H -3.71002 -0.43148 -1.28330
C -5.83049 -0.73238 -1.09361
H -6.54966 -1.46118 -0.68143
H -6.03327 -0.62515 -2.17268
H -6.02396 0.24044 -0.61270
C -4.11630 -2.52060 -1.63680
H -4.83899 -3.30765 -1.36098
H -3.10329 -2.91973 -1.45167
H -4.22329 -2.34519 -2.71998
C -2.72133 0.82739 3.58688
H -2.14438 1.46985 2.89770
C -3.86960 1.67905 4.18377
H -4.48690 1.07482 4.87079
H -4.53316 2.08376 3.40264
H -3.46038 2.53023 4.75410
C -1.76650 0.37735 4.71220
H -1.28583 1.25643 5.17308

```
H  -0.97080 -0.29074  4.33921
H  -2.29851 -0.15940  5.51632
C   1.76572 -1.14817  0.03719
C   3.25541 -2.87749 -0.32023
H   3.57581 -3.85212 -0.67646
C   3.92430 -1.89775  0.35046
H   4.94805 -1.84616  0.70834
C   3.54866  0.39554  1.13614
C   3.58502  0.52730  2.54857
C   4.16353  1.69511  3.08491
H   4.19914  1.81966  4.17158
C   4.71474  2.67785  2.25868
H   5.16034  3.57736  2.69526
C   4.72914  2.49018  0.87231
H   5.20365  3.24111  0.23486
C   4.17271  1.34210  0.27390
C   4.38129  1.11978 -1.22682
H   3.59430  0.43569 -1.58952
C   4.29713  2.41974 -2.05653
H   3.39478  3.00431 -1.82421
H   4.27592  2.17270 -3.13081
H   5.18123  3.06034 -1.89397
C   5.75469  0.44567 -1.48215
H   6.57373  1.08336 -1.10740
H   5.91010  0.29648 -2.56441
H   5.84171 -0.53710 -0.99216
C   3.13162 -0.57025  3.50633
H   2.58130 -1.33392  2.92735
C   2.18242 -0.04808  4.60420
H   2.70070  0.63284  5.30079
H   1.78669 -0.88891  5.19811
H   1.32918  0.50448  4.17485
C   4.35305 -1.28221  4.13739
H   5.01961 -1.71044  3.37010
H   4.02110 -2.10215  4.79686
H   4.95033 -0.57904  4.74306
C   1.04229 -3.16844 -1.36658
C   0.26227 -4.20617 -0.80024
C  -0.59527 -4.91864 -1.66433
H  -1.20963 -5.72756 -1.25683
C  -0.66420 -4.62003 -3.02926
H  -1.34085 -5.18238 -3.68047
C   0.15606 -3.62039 -3.57027
H   0.12255 -3.42090 -4.64511
C   1.04299 -2.88169 -2.76101
C   2.03059 -1.90482 -3.40502
H   2.36648 -1.19465 -2.62869
C   3.27681 -2.67480 -3.91229
H   3.78394 -3.22343 -3.10203
H   4.00516 -1.97490 -4.35577
H   2.99237 -3.40633 -4.68814
C   1.42526 -1.07746 -4.55798
H   1.19100 -1.70394 -5.43569
H   2.14820 -0.31365 -4.88823
H   0.49726 -0.56040 -4.26072
C   0.36378 -4.61823  0.66655
H   0.94688 -3.84675  1.20327
C  -1.01327 -4.71973  1.35629
H  -1.61696 -5.54459  0.94017
H  -1.60029 -3.79110  1.24855
H  -0.88267 -4.91793  2.43317
C   1.14135 -5.95001  0.80376
H   1.24957 -6.22321  1.86702
H   2.15065 -5.88322  0.36409
H   0.60934 -6.77192  0.29421
C   0.76547  1.04076 -1.35687
```

[5^{+}_{1}] (+2.5 kcal/mol)

```
140
SCF  =           -2985.86181653
H(0 K)=          -2984.704768
G(298 K)=        -2984.815296
SCF(CORR) =      -6091.99214192
Low Freq. = 12.4289cm-1,
22.9350cm-1

Ru -0.04081  0.12274 -0.05464
H   0.49233 -1.49414 -0.51729
H  -0.56280  1.40402  0.77926
H  -0.28950 -0.31306  1.57292
Zn  0.43570 -1.91578  1.45007
H   1.09506 -3.06349  2.21030
Zn  0.38839 -0.98080 -2.27946
H   0.35063 -1.44678 -3.73831
O   0.16703  2.49158 -1.96642
N  -2.71568 -1.59364  0.01153
N  -3.11071  0.32952 -0.91029
N   3.15331  0.32615 -0.30722
N   2.46943  1.40025  1.45306
C  -2.07904 -0.40245 -0.33645
C  -4.07169 -1.58143 -0.33202
H  -4.72183 -2.42523 -0.12152
C  -4.31663 -0.37496 -0.90982
H  -5.22738  0.05865 -1.31148
C  -3.09476  1.70571 -1.37632
C  -3.40376  2.72655 -0.43981
C  -3.46128  4.04790 -0.92471
H  -3.69763  4.86196 -0.23223
C  -3.25137  4.33304 -2.27952
H  -3.30757  5.36709 -2.63473
C  -2.99245  3.29770 -3.18587
H  -2.85201  3.53268 -4.24491
C  -2.91349  1.95762 -2.76034
C  -2.69695  0.83503 -3.77751
H  -2.30424 -0.04441 -3.23173
C  -4.03858  0.41961 -4.43096
H  -4.77283  0.06921 -3.68734
H  -3.87687 -0.39550 -5.15701
H  -4.48656  1.27180 -4.97065
C  -1.67155  1.19707 -4.87227
H  -0.73232  1.57951 -4.44189
H  -2.06380  1.96460 -5.56148
H  -1.43650  0.30556 -5.47703
C  -3.78061  2.42638  1.01272
```

H	-3.48455	1.38581	1.23374
C	-5.31576	2.53145	1.19070
H	-5.66567	3.55823	0.98718
H	-5.60348	2.27524	2.22483
H	-5.85664	1.85403	0.50883
C	-3.05722	3.32769	2.03308
H	-1.96293	3.22538	1.96047
H	-3.35659	3.04745	3.05750
H	-3.31480	4.39253	1.89922
C	-2.18705	-2.72629	0.74519
C	-2.40301	-2.76843	2.15429
C	-1.99444	-3.93393	2.83660
H	-2.14481	-3.99839	3.91882
C	-1.42989	-5.01631	2.15219
H	-1.12535	-5.91134	2.70358
C	-1.27031	-4.96659	0.76112
H	-0.86019	-5.83435	0.23616
C	-1.65785	-3.83236	0.01898
C	-1.62962	-3.85810	-1.51026
H	-1.68104	-2.81188	-1.86728
C	-2.88364	-4.59249	-2.04925
H	-2.89238	-5.64466	-1.71611
H	-2.88570	-4.58399	-3.15227
H	-3.81854	-4.12136	-1.70495
C	-0.35418	-4.49283	-2.09829
H	-0.27314	-5.56475	-1.84941
H	0.55982	-3.99174	-1.73750
H	-0.36873	-4.41232	-3.19755
C	-3.15897	-1.66922	2.90535
H	-3.30169	-0.81942	2.21595
C	-4.56135	-2.18339	3.31570
H	-4.48487	-3.02351	4.02724
H	-5.14179	-2.53359	2.44600
H	-5.13453	-1.37808	3.80511
C	-2.39671	-1.13589	4.13515
H	-3.00036	-0.36770	4.64728
H	-1.44162	-0.66926	3.84413
H	-2.18462	-1.93174	4.86957
C	1.99484	0.61942	0.41063
C	4.27989	0.91190	0.27548
H	5.27104	0.79094	-0.15029
C	3.84996	1.58674	1.37670
H	4.38606	2.18410	2.10762
C	1.71148	2.01638	2.53016
C	1.61578	1.32932	3.76856
C	0.91235	1.97216	4.80656
H	0.81498	1.47471	5.77615
C	0.36050	3.24701	4.62874
H	-0.17627	3.72863	5.45253
C	0.52408	3.92166	3.41279
H	0.12646	4.93493	3.30071
C	1.21198	3.32943	2.33494
C	1.49604	4.13679	1.06644
H	1.79673	3.43125	0.27254
C	0.27426	4.91859	0.54338
H	-0.58220	4.25611	0.34303
H	0.53473	5.42694	-0.40000
H	-0.05006	5.69834	1.25412
C	2.68231	5.10264	1.31376
H	2.43426	5.83438	2.10198
H	2.92039	5.66308	0.39354
H	3.59292	4.56720	1.63061
C	2.33782	0.00465	4.03343
H	2.52818	-0.48409	3.05887
C	1.53108	-0.98508	4.89767
H	1.39118	-0.61224	5.92684
H	2.06991	-1.94445	4.96905
H	0.53563	-1.19076	4.47348
C	3.71082	0.27002	4.70249
H	4.35710	0.91735	4.08829
H	4.24625	-0.67966	4.87259
H	3.57458	0.76413	5.67993
C	3.30616	-0.47893	-1.51036
C	3.54680	-1.87675	-1.36707
C	3.75787	-2.62139	-2.54478
H	3.93537	-3.69793	-2.47060
C	3.78111	-2.00589	-3.80305
H	3.95236	-2.60653	-4.70191
C	3.61136	-0.62255	-3.90890
H	3.66558	-0.14731	-4.89229
C	3.38085	0.18039	-2.77119
C	3.33075	1.70254	-2.92354
H	2.84327	2.12311	-2.02706
C	4.76769	2.27957	-2.99895
H	5.36216	2.04524	-2.10127
H	4.73109	3.37734	-3.10177
H	5.30504	1.87437	-3.87367
C	2.52684	2.16269	-4.15840
H	3.05039	1.92274	-5.09972
H	2.39378	3.25651	-4.13040
H	1.52822	1.70042	-4.19696
C	3.70836	-2.54666	-0.00051
H	3.13571	-1.95889	0.74182
C	3.18766	-3.99604	0.05251
H	3.79662	-4.67478	-0.56904
H	2.13939	-4.07463	-0.27771
H	3.23684	-4.36964	1.08823
C	5.19841	-2.51907	0.42902
H	5.31674	-2.98424	1.42235
H	5.59772	-1.49413	0.48650
H	5.81815	-3.08455	-0.28810
C	0.04182	1.52058	-1.31881

TS(5^+_1-5^+_2) (+6.0 kcal/mol)

140

SCF = -2985.85440100

H(0 K)= -2984.700096

G(298 K)= -2984.809850

SCF(CORR) = -6091.98459103

Low Freq. = -495.8983cm-1, 14.3193cm-1

Ru	-0.04194	0.12276	-0.04856
H	0.39896	-1.51717	-0.24631

H	-0.47925	1.30991	1.04621
H	-0.26788	0.41381	1.60985
Zn	0.48838	-1.59597	1.72853
H	1.09590	-2.49651	2.81470
Zn	0.13528	-1.15636	-2.17969
H	-0.04812	-1.88986	-3.51920
O	0.03713	2.33531	-2.17672
N	-2.72822	-1.55870	0.36199
N	-3.16033	0.26590	-0.72940
N	3.12542	0.12683	-0.54007
N	2.60989	1.55923	1.01007
C	-2.10189	-0.42106	-0.14593
C	-4.10151	-1.56060	0.09906
H	-4.74552	-2.36803	0.43427
C	-4.37049	-0.41506	-0.58338
H	-5.29992	-0.01071	-0.97260
C	-3.15916	1.58174	-1.34631
C	-3.34398	2.71196	-0.50758
C	-3.42631	3.97143	-1.13259
H	-3.56998	4.86558	-0.51802
C	-3.35679	4.09576	-2.52576
H	-3.43025	5.08451	-2.98995
C	-3.21400	2.95751	-3.32781
H	-3.17967	3.06682	-4.41572
C	-3.11670	1.67187	-2.76120
C	-3.01767	0.44005	-3.66298
H	-2.62106	-0.39485	-3.05423
C	-4.41555	0.02087	-4.18154
H	-5.11152	-0.21530	-3.36003
H	-4.33525	-0.87250	-4.82418
H	-4.86585	0.83115	-4.78063
C	-2.05590	0.64323	-4.85274
H	-1.07758	1.02934	-4.52459
H	-2.46577	1.35196	-5.59280
H	-1.89228	-0.31499	-5.37305
C	-3.55795	2.59088	1.00242
H	-3.22831	1.58346	1.31329
C	-5.06508	2.71862	1.33629
H	-5.44907	3.71063	1.04211
H	-5.23293	2.59894	2.42047
H	-5.66781	1.95817	0.81225
C	-2.73780	3.61056	1.81819
H	-1.65851	3.52828	1.61090
H	-2.89091	3.43829	2.89719
H	-3.04704	4.64922	1.60945
C	-2.17087	-2.60768	1.19399
C	-2.33297	-2.49555	2.60496
C	-1.88942	-3.57620	3.39606
H	-1.99804	-3.52096	4.48372
C	-1.33051	-4.71910	2.81524
H	-0.99194	-5.54419	3.44964
C	-1.22038	-4.82024	1.42141
H	-0.81622	-5.73594	0.97983
C	-1.65605	-3.78176	0.57410
C	-1.69051	-3.97737	-0.94221
H	-1.74925	-2.97717	-1.41206
C	-2.97245	-4.75028	-1.34458

H	-2.97564	-5.75953	-0.89782
H	-3.02181	-4.86205	-2.44087
H	-3.88776	-4.23216	-1.01501
C	-0.44615	-4.68694	-1.51014
H	-0.36378	-5.72722	-1.15114
H	0.48696	-4.16109	-1.24736
H	-0.50897	-4.72449	-2.60996
C	-3.04829	-1.31428	3.26544
H	-3.24042	-0.55185	2.49070
C	-4.41850	-1.76576	3.82748
H	-4.29368	-2.51866	4.62455
H	-5.05629	-2.20975	3.04517
H	-4.95762	-0.90526	4.25827
C	-2.20377	-0.64405	4.36876
H	-2.75686	0.20294	4.80866
H	-1.25484	-0.25288	3.96495
H	-1.96162	-1.34326	5.18712
C	2.03296	0.60989	0.17449
C	4.31196	0.75993	-0.16147
H	5.26366	0.50907	-0.61954
C	3.98753	1.65843	0.80770
H	4.59615	2.36106	1.36831
C	1.96634	2.39839	2.00997
C	1.93054	1.94165	3.35530
C	1.34486	2.79648	4.31030
H	1.29571	2.47707	5.35530
C	0.84993	4.05641	3.95227
H	0.40564	4.70511	4.71414
C	0.95242	4.50085	2.62911
H	0.59965	5.50308	2.36835
C	1.52109	3.69031	1.62572
C	1.73626	4.25542	0.22031
H	1.90781	3.40932	-0.46710
C	0.52586	5.04746	-0.31565
H	-0.39959	4.45086	-0.29689
H	0.71143	5.34642	-1.36055
H	0.35372	5.97279	0.26102
C	3.00389	5.14682	0.19839
H	2.88786	6.00896	0.87773
H	3.18062	5.53722	-0.81839
H	3.90561	4.59427	0.51061
C	2.59076	0.63435	3.80315
H	2.63686	-0.04491	2.93040
C	1.82116	-0.09784	4.92074
H	1.85817	0.45660	5.87423
H	2.27421	-1.08659	5.10030
H	0.76282	-0.25752	4.66080
C	4.04474	0.89847	4.27203
H	4.66605	1.34587	3.48010
H	4.52373	-0.04505	4.58470
H	4.05234	1.58650	5.13497
C	3.16236	-0.90018	-1.56841
C	3.38893	-2.24908	-1.17379
C	3.50315	-3.21088	-2.19714
H	3.67368	-4.25775	-1.92962
C	3.43675	-2.84912	-3.54914
H	3.53430	-3.61524	-4.32480

```
C   3.26879 -1.50852 -3.90911
H   3.24653 -1.23343 -4.96740
C   3.13616 -0.49743 -2.93357
C   3.07262  0.96947 -3.36528
H   2.68136  1.55940 -2.51838
C   4.49297  1.49463 -3.69404
H   5.17647  1.41629 -2.83298
H   4.44709  2.55544 -3.99347
H   4.93653  0.92502 -4.52889
C   2.13804  1.20387 -4.57069
H   2.54820  0.76948 -5.49851
H   2.01258  2.28529 -4.74321
H   1.13955  0.76668 -4.40837
C   3.62744 -2.64934  0.28395
H   3.16159 -1.88273  0.93206
C   3.01581 -4.01214  0.66456
H   3.50907 -4.84641  0.13684
H   1.93635 -4.05921  0.44606
H   3.14259 -4.18769  1.74527
C   5.14726 -2.65651  0.58999
H   5.32308 -2.91962  1.64684
H   5.61344 -1.67579  0.40313
H   5.66549 -3.40191 -0.03767
C  -0.07356  1.45447 -1.40762
```

[5^{+}_{2}] (+6.4 kcal/mol)

```
140
SCF  =           -2985.85449137
H(0 K)=          -2984.698820
G(298 K)=        -2984.809009
SCF(CORR) =      -6091.98481126
Low Freq. = 13.4447cm-1,
22.0224cm-1
```

```
Ru -0.04226  0.12846 -0.04323
H   0.36152 -1.51856 -0.18859
H  -0.45885  1.32975  1.09600
H  -0.27080  0.53589  1.62155
Zn  0.48417 -1.59251  1.74116
H   1.07397 -2.45867  2.86643
Zn  0.09562 -1.15138 -2.16463
H  -0.10509 -1.90913 -3.48904
O   0.05131  2.32892 -2.18439
N  -2.74932 -1.51921  0.38820
N  -3.16255  0.31133 -0.70108
N   3.12043  0.06348 -0.56244
N   2.64723  1.52732  0.97173
C  -2.10967 -0.39073 -0.12434
C  -4.12406 -1.50110  0.13445
H  -4.77740 -2.29898  0.47440
C  -4.38133 -0.35189 -0.54643
H  -5.30756  0.06539 -0.92962
C  -3.14608  1.62443 -1.32364
C  -3.30226  2.76180 -0.48864
C  -3.37105  4.01949 -1.11877
H  -3.49265  4.91882 -0.50704
C  -3.31587  4.13606 -2.51327
H  -3.37851  5.12368 -2.98144
C  -3.20089  2.99181 -3.31113
H  -3.17710  3.09529 -4.39992
C  -3.11811  1.70741 -2.73936
C  -3.04812  0.47045 -3.63674
H  -2.66427 -0.36972 -3.02722
C  -4.45670  0.07669 -4.14609
H  -5.15271 -0.14240 -3.31988
H  -4.39735 -0.82069 -4.78538
H  -4.89440  0.89312 -4.74619
C  -2.08897  0.65095 -4.83236
H  -1.10162  1.01907 -4.51065
H  -2.48885  1.36516 -5.57262
H  -1.94678 -0.31201 -5.35022
C  -3.49955  2.65148  1.02439
H  -3.17710  1.64216  1.33697
C  -5.00118  2.79628  1.37569
H  -5.37871  3.79014  1.07940
H  -5.15720  2.68533  2.46256
H  -5.61740  2.03836  0.86389
C  -2.65995  3.66790  1.82449
H  -1.58441  3.58037  1.59983
H  -2.79645  3.49923  2.90628
H  -2.96657  4.70784  1.61838
C  -2.20335 -2.57656  1.21734
C  -2.35248 -2.45999  2.62908
C  -1.92113 -3.54684  3.41840
H  -2.02044 -3.48838  4.50677
C  -1.38465 -4.69906  2.83505
H  -1.05458 -5.52873  3.46795
C  -1.28632 -4.80332  1.44047
H  -0.89985 -5.72571  0.99696
C  -1.71200 -3.75914  0.59509
C  -1.75924 -3.95551 -0.92079
H  -1.80467 -2.95505 -1.39149
C  -3.05560 -4.70852 -1.31461
H  -3.07199 -5.71719 -0.86681
H  -3.11328 -4.82056 -2.41049
H  -3.96079 -4.17590 -0.98018
C  -0.52979 -4.68513 -1.49578
H  -0.46197 -5.72646 -1.13670
H   0.41320 -4.17395 -1.23952
H  -0.59995 -4.72189 -2.59516
C  -3.04108 -1.26520  3.29358
H  -3.22214 -0.49885  2.51992
C  -4.41699 -1.68922  3.86294
H  -4.30283 -2.44607  4.65790
H  -5.06828 -2.11856  3.08361
H  -4.93587 -0.81869  4.29844
C  -2.17729 -0.61279  4.39266
H  -2.70774  0.24904  4.83166
H  -1.21932 -0.24776  3.98549
H  -1.95018 -1.31546  5.21232
C   2.04378  0.57797  0.15380
C   4.32292  0.67694 -0.20195
H   5.26546  0.39997 -0.66389
C   4.02499  1.59524  0.75699
```

H	4.65248	2.29289	1.30284
C	2.02976	2.39251	1.96598
C	1.99568	1.95387	3.31765
C	1.43429	2.83209	4.26605
H	1.38702	2.52680	5.31531
C	0.96086	4.09672	3.89580
H	0.53508	4.76343	4.65268
C	1.06148	4.52222	2.56634
H	0.72623	5.52775	2.29548
C	1.60671	3.68827	1.56899
C	1.82241	4.23277	0.15552
H	1.96764	3.37561	-0.52415
C	0.62663	5.04840	-0.37806
H	-0.31382	4.47654	-0.34105
H	0.80793	5.32820	-1.42897
H	0.48498	5.98569	0.18745
C	3.11067	5.09339	0.11270
H	3.02149	5.96499	0.78399
H	3.28700	5.46893	-0.90971
H	4.00188	4.52273	0.42238
C	2.63210	0.63907	3.77767
H	2.65122	-0.05462	2.91533
C	1.86049	-0.05737	4.91654
H	1.92784	0.50805	5.86191
H	2.28913	-1.05581	5.10200
H	0.79423	-0.19125	4.67511
C	4.09752	0.87684	4.22460
H	4.71959	1.29590	3.41797
H	4.55789	-0.07244	4.54762
H	4.13165	1.57873	5.07566
C	3.12812	-0.97482	-1.57988
C	3.33196	-2.32354	-1.17305
C	3.42245	-3.29737	-2.18732
H	3.57571	-4.34448	-1.91035
C	3.35460	-2.94775	-3.54243
H	3.43390	-3.72310	-4.31096
C	3.20788	-1.60800	-3.91488
H	3.18323	-1.34317	-4.97575
C	3.09937	-0.58523	-2.94877
C	3.05539	0.87837	-3.39386
H	2.68014	1.48168	-2.54911
C	4.48065	1.37856	-3.73959
H	5.17024	1.29771	-2.88364
H	4.44848	2.43712	-4.04863
H	4.90821	0.79449	-4.57280
C	2.11378	1.11619	-4.59310
H	2.50796	0.66572	-5.52018
H	2.00453	2.19778	-4.77536
H	1.10979	0.69708	-4.41729
C	3.57169	-2.71353	0.28729
H	3.12908	-1.92882	0.93019
C	2.93029	-4.05714	0.68699
H	3.39880	-4.90850	0.16403
H	1.84827	-4.08074	0.47667
H	3.06120	-4.22475	1.76845
C	5.09292	-2.75311	0.58323
H	5.27001	-3.00862	1.64173
H	5.58037	-1.78560	0.38219
H	5.58922	-3.51722	-0.03963
C	-0.07108	1.46447	-1.39817

140

TS(5$^+_2$-5$^+_2$') (+9.2 kcal/mol)

SCF = -2985.84941383
H(0 K)= -2984.693624
G(298 K)= -2984.804770
SCF(CORR) = -6091.97957984
Low Freq. = -325.8666cm-1, 10.7206cm-1

Ru	-0.01949	0.17355	0.01666
H	0.04675	-1.51547	0.03457
H	-0.54548	1.13009	1.39677
H	0.31506	1.25176	1.39414
Zn	0.29824	-1.54065	1.87080
H	0.76751	-2.35563	3.09347
Zn	-0.22758	-1.14851	-2.05909
H	-0.65828	-1.89951	-3.33670
O	0.32712	2.29403	-2.18008
N	-2.96735	-0.99666	0.53412
N	-3.08588	0.88670	-0.53875
N	3.01322	-0.59777	-0.70141
N	2.97532	1.01795	0.75104
C	-2.15640	0.00105	-0.00416
C	-4.32444	-0.72729	0.33610
H	-5.09683	-1.39733	0.70121
C	-4.39710	0.45440	-0.33363
H	-5.24672	1.03490	-0.68000
C	-2.86050	2.16703	-1.18918
C	-2.73877	3.32358	-0.37519
C	-2.61580	4.56378	-1.03096
H	-2.52105	5.47710	-0.43553
C	-2.64186	4.65125	-2.42840
H	-2.55284	5.62732	-2.91608
C	-2.79917	3.49599	-3.20258
H	-2.83343	3.57919	-4.29290
C	-2.91629	2.22595	-2.60549
C	-3.13079	0.98750	-3.47757
H	-2.89585	0.09576	-2.86646
C	-4.60809	0.87541	-3.92801
H	-5.30114	0.81149	-3.07314
H	-4.75311	-0.02500	-4.54904
H	-4.89875	1.75355	-4.53023
C	-2.20115	0.96012	-4.70965
H	-1.14919	1.12742	-4.42840
H	-2.47842	1.73215	-5.44796
H	-2.27278	-0.01753	-5.21441
C	-2.83620	3.27242	1.15040
H	-2.68067	2.22495	1.46687
C	-4.25521	3.69178	1.60801
H	-4.47111	4.73198	1.30913
H	-4.34253	3.62991	2.70639
H	-5.03450	3.04804	1.16705
C	-1.76597	4.13084	1.85578

H -0.74563 3.85896 1.53935
H -1.83362 3.99389 2.94851
H -1.90724 5.20704 1.65668
C -2.59584 -2.14298 1.34312
C -2.63068 -1.99667 2.75888
C -2.37729 -3.14669 3.53567
H -2.39414 -3.06824 4.62701
C -2.11789 -4.38337 2.93624
H -1.92161 -5.26177 3.55901
C -2.12973 -4.50587 1.53973
H -1.96294 -5.48715 1.08556
C -2.38921 -3.39845 0.70770
C -2.55793 -3.58514 -0.80079
H -2.43952 -2.59635 -1.28290
C -3.99131 -4.08418 -1.11531
H -4.17082 -5.07041 -0.65331
H -4.12854 -4.18779 -2.20495
H -4.76288 -3.39162 -0.74108
C -1.52032 -4.53460 -1.43103
H -1.63388 -5.57009 -1.06637
H -0.48558 -4.21198 -1.22882
H -1.65287 -4.55541 -2.52506
C -3.02532 -0.68686 3.44615
H -3.04749 0.10873 2.68061
C -4.44897 -0.80334 4.04350
H -4.48784 -1.58297 4.82358
H -5.19651 -1.06097 3.27511
H -4.75097 0.15199 4.50508
C -2.01884 -0.25331 4.53179
H -2.31785 0.71764 4.96150
H -1.00218 -0.14557 4.11735
H -1.96531 -0.97950 5.36052
C 2.12456 0.17878 0.03405
C 4.34231 -0.24931 -0.44631
H 5.16820 -0.75559 -0.93632
C 4.31693 0.76494 0.46011
H 5.11718 1.33394 0.92286
C 2.62970 2.05849 1.70912
C 2.59520 1.72498 3.09210
C 2.31130 2.76456 4.00008
H 2.27010 2.54239 5.07010
C 2.10541 4.07797 3.56198
H 1.89236 4.86897 4.28831
C 2.20240 4.38649 2.20054
H 2.07827 5.42271 1.87295
C 2.47872 3.39208 1.24066
C 2.70540 3.79073 -0.21856
H 2.58501 2.88959 -0.84386
C 1.70182 4.84698 -0.72676
H 0.65750 4.53710 -0.56487
H 1.84225 5.00147 -1.80920
H 1.85406 5.82460 -0.23743
C 4.15478 4.30670 -0.40765
H 4.33430 5.20386 0.20963
H 4.32881 4.57994 -1.46238
H 4.90580 3.55023 -0.12606
C 2.94152 0.33021 3.61888
H 2.72723 -0.40358 2.81834
C 2.11905 -0.07963 4.85724
H 2.41216 0.49702 5.75147
H 2.28978 -1.14457 5.08381
H 1.03756 0.06080 4.70147
C 4.45228 0.23572 3.95368
H 5.08953 0.43026 3.07667
H 4.69696 -0.77151 4.33178
H 4.72049 0.96726 4.73538
C 2.73049 -1.64707 -1.66547
C 2.66582 -2.99266 -1.20694
C 2.49836 -3.99840 -2.17927
H 2.44324 -5.04405 -1.86221
C 2.43763 -3.68949 -3.54470
H 2.31508 -4.49056 -4.28045
C 2.55296 -2.36253 -3.97061
H 2.52746 -2.13545 -5.04021
C 2.71031 -1.30701 -3.04796
C 2.93959 0.11921 -3.55440
H 2.75815 0.81374 -2.71546
C 4.40870 0.29958 -4.01206
H 5.12569 0.10223 -3.19854
H 4.57390 1.33159 -4.36526
H 4.64604 -0.38565 -4.84410
C 1.98182 0.51193 -4.69903
H 2.19966 -0.04517 -5.62639
H 2.09077 1.58477 -4.92773
H 0.92826 0.32393 -4.43560
C 2.89031 -3.37135 0.25846
H 2.69212 -2.47601 0.87827
C 1.96302 -4.49730 0.75779
H 2.16928 -5.45536 0.25040
H 0.89730 -4.25640 0.60690
H 2.11866 -4.65770 1.83714
C 4.37094 -3.77063 0.48350
H 4.54400 -4.01583 1.54508
H 5.06538 -2.96151 0.20465
H 4.62947 -4.65904 -0.11822
C 0.09510 1.52248 -1.32393

(iii) Intermolecular H/D Exchange

2
H_2
SCF = -1.17646513234
H(0 K)= -1.166540
G(298 K)= -1.178046
SCF(CORR) = -1.17781477501
Low Freq. = 4356.8361cm-1

H 0.00000 0.00000 0.37523
H 0.00000 0.00000 -0.37523

TS(5^+_2-5^+-H_2) (+24.1 kcal/mol)
140
SCF = -2985.82401586
H(0 K)= -2984.670434

G(298 K)= -2984.782374
SCF(CORR) = -6091.95273951
Low Freq. = -99.1614cm-1, 14.4042cm-1

Ru	0.00661	0.12884	-0.05232
H	-0.12911	-1.57120	0.11681
H	-0.54491	2.12026	3.49149
H	-1.24266	1.85265	3.40925
Zn	0.02984	-1.70838	1.80791
H	0.33471	-2.33038	3.17499
Zn	-0.14415	-1.09469	-2.10661
H	-0.45554	-1.82129	-3.42698
O	0.64602	2.37514	-2.00980
N	-3.03884	-0.68856	0.44182
N	-2.92223	1.20360	-0.62738
N	3.02394	-0.86220	-0.46830
N	2.96864	0.77629	0.95529
C	-2.09659	0.21671	-0.07886
C	-4.34873	-0.26563	0.21464
H	-5.20257	-0.84684	0.54880
C	-4.27363	0.91920	-0.45175
H	-5.04815	1.58778	-0.81538
C	-2.55617	2.42236	-1.33163
C	-2.37347	3.61231	-0.57951
C	-2.12389	4.79766	-1.29812
H	-1.97857	5.73405	-0.75138
C	-2.08189	4.80436	-2.69740
H	-1.89237	5.73968	-3.23385
C	-2.29815	3.62093	-3.41212
H	-2.27828	3.64155	-4.50578
C	-2.54638	2.40290	-2.75083
C	-2.83231	1.13961	-3.56484
H	-2.69447	0.26712	-2.89860
C	-4.29762	1.12453	-4.06555
H	-5.02095	1.16429	-3.23463
H	-4.49737	0.20714	-4.64532
H	-4.49283	1.98997	-4.72216
C	-1.86651	0.96872	-4.75708
H	-0.81327	1.05544	-4.44390
H	-2.04687	1.72452	-5.54072
H	-2.00682	-0.02264	-5.21872
C	-2.52703	3.65695	0.94134
H	-2.40010	2.62851	1.32521
C	-3.95078	4.13479	1.32192
H	-4.13103	5.15872	0.95136
H	-4.07485	4.14471	2.41852
H	-4.73242	3.48281	0.89781
C	-1.46712	4.53920	1.63065
H	-0.44267	4.21918	1.38037
H	-1.58293	4.47746	2.72541
H	-1.57352	5.60232	1.35439
C	-2.83589	-1.92979	1.17037
C	-2.96218	-1.91213	2.59058
C	-2.88577	-3.14947	3.26583
H	-2.97463	-3.16761	4.35568
C	-2.70363	-4.34782	2.57036
H	-2.64236	-5.29404	3.11725
C	-2.62324	-4.34236	1.17060
H	-2.51989	-5.29075	0.63550
C	-2.71530	-3.14489	0.43452
C	-2.80014	-3.18874	-1.09180
H	-2.50411	-2.19527	-1.47867
C	-4.26506	-3.43993	-1.53270
H	-4.62081	-4.41688	-1.16226
H	-4.33505	-3.44672	-2.63358
H	-4.95012	-2.66406	-1.15438
C	-1.87218	-4.23738	-1.73660
H	-2.18133	-5.26769	-1.48964
H	-0.82112	-4.11102	-1.42736
H	-1.90766	-4.13957	-2.83371
C	-3.26218	-0.63830	3.38561
H	-2.99541	0.22811	2.75283
C	-4.77408	-0.54421	3.71291
H	-5.09187	-1.40253	4.32965
H	-5.40051	-0.52953	2.80714
H	-4.98327	0.37737	4.28204
C	-2.44785	-0.54329	4.69349
H	-2.59055	0.44792	5.15495
H	-1.37074	-0.69375	4.51860
H	-2.77515	-1.29324	5.43380
C	2.12889	-0.00828	0.16875
C	4.34502	-0.61232	-0.08944
H	5.17431	-1.18427	-0.49465
C	4.30885	0.41463	0.80340
H	5.10108	0.92391	1.34339
C	2.61126	1.85896	1.86323
C	2.41621	1.55376	3.23877
C	2.15302	2.62959	4.11064
H	1.99486	2.42908	5.17402
C	2.11608	3.94975	3.64692
H	1.91393	4.76890	4.34458
C	2.37228	4.22559	2.29949
H	2.38448	5.26392	1.95613
C	2.64458	3.19532	1.37670
C	3.07597	3.55657	-0.04649
H	2.91550	2.67497	-0.69027
C	2.27609	4.73019	-0.65084
H	1.18913	4.56505	-0.59496
H	2.54295	4.85163	-1.71355
H	2.51014	5.68469	-0.14829
C	4.58927	3.89314	-0.07146
H	4.80664	4.76100	0.57486
H	4.90676	4.14612	-1.09746
H	5.21078	3.05173	0.27585
C	2.58890	0.14400	3.81005
H	2.37983	-0.58279	3.00211
C	1.62760	-0.16346	4.97567
H	1.90303	0.39231	5.88862
H	1.66901	-1.23673	5.22374
H	0.58489	0.08905	4.72711
C	4.04893	-0.07943	4.28073
H	4.77658	0.03350	3.46184
H	4.16444	-1.09515	4.69624

H	4.31398	0.64352	5.07142
C	2.74538	-1.87125	-1.47446
C	2.51760	-3.21059	-1.05418
C	2.34990	-4.18437	-2.05838
H	2.17245	-5.22518	-1.77117
C	2.44169	-3.85175	-3.41645
H	2.31650	-4.62805	-4.17789
C	2.71115	-2.53401	-3.80089
H	2.80107	-2.29055	-4.86355
C	2.87543	-1.51042	-2.84502
C	3.25637	-0.09815	-3.29575
H	3.07526	0.58970	-2.45114
C	4.76311	-0.03263	-3.64877
H	5.40150	-0.31418	-2.79532
H	5.03975	0.99026	-3.95572
H	4.99961	-0.71310	-4.48499
C	2.41195	0.40078	-4.48750
H	2.62820	-0.16392	-5.41059
H	2.63803	1.46057	-4.69008
H	1.33102	0.31478	-4.28817
C	2.56625	-3.62444	0.41774
H	2.40820	-2.71621	1.03168
C	1.48288	-4.65075	0.80669
H	1.63718	-5.62133	0.30482
H	0.46671	-4.30293	0.54991
H	1.51295	-4.83745	1.89290
C	3.96960	-4.17695	0.77283
H	4.01709	-4.44892	1.84098
H	4.76433	-3.43965	0.57422
H	4.19251	-5.08085	0.18008
C	0.31302	1.59994	-1.18943

[5^+-H_2] (+13.2 kcal/mol)
138
SCF = -2984.65371057
H(0 K)= -2983.512731
G(298 K)= -2983.621266
SCF(CORR) = -6090.78142089
Low Freq. = 18.2532cm-1, 23.1214cm-1

Ru	0.04108	0.14727	-0.07405
H	-1.06841	-1.10233	0.23365
Zn	-0.83641	-1.19091	1.95433
H	-0.90189	-1.84403	3.34528
Zn	-0.96628	-0.81049	-2.03985
H	-1.71559	-1.16192	-3.33819
O	1.75764	1.31356	-2.31980
N	-2.92569	1.30821	0.30848
N	-1.61307	2.75879	-0.63945
N	1.71947	-2.54527	-0.41996
N	2.84444	-1.15936	0.81778
C	-1.60254	1.46441	-0.12464
C	-3.69049	2.44886	0.05242
H	-4.74070	2.50270	0.32255
C	-2.86715	3.35910	-0.53762
H	-3.04479	4.37424	-0.88029
C	-0.47307	3.51564	-1.11397
C	0.42584	4.03599	-0.14526
C	1.50911	4.80069	-0.61642
H	2.22558	5.21746	0.09737
C	1.66514	5.06643	-1.98445
H	2.50822	5.67420	-2.32828
C	0.73571	4.57701	-2.90883
H	0.86036	4.80695	-3.97161
C	-0.35722	3.78741	-2.49952
C	-1.36424	3.27976	-3.53333
H	-2.03082	2.55478	-3.03231
C	-2.24088	4.43949	-4.06454
H	-2.78167	4.95657	-3.25391
H	-2.98715	4.06052	-4.78331
H	-1.62444	5.19322	-4.58420
C	-0.68227	2.54146	-4.70693
H	-0.04056	1.71946	-4.35188
H	-0.05443	3.22213	-5.30720
H	-1.44639	2.11858	-5.38077
C	0.20897	3.81031	1.35264
H	-0.87178	3.63255	1.50536
C	0.58590	5.04309	2.20520
H	1.67460	5.22113	2.21731
H	0.27169	4.88825	3.25099
H	0.09504	5.95655	1.83159
C	0.96323	2.56904	1.87420
H	0.60491	1.63103	1.36141
H	0.79316	2.42834	2.95468
H	2.04713	2.64010	1.69324
C	-3.51556	0.22430	1.07557
C	-3.50422	0.32600	2.49756
C	-4.15481	-0.69279	3.22578
H	-4.15815	-0.64826	4.31858
C	-4.80767	-1.74612	2.57664
H	-5.30851	-2.52285	3.16323
C	-4.84184	-1.79493	1.17697
H	-5.38458	-2.60498	0.68112
C	-4.21045	-0.80956	0.39165
C	-4.36789	-0.82652	-1.12912
H	-3.55120	-0.21913	-1.56375
C	-5.70644	-0.16247	-1.53997
H	-6.56251	-0.71888	-1.12060
H	-5.80814	-0.15734	-2.63842
H	-5.77752	0.87996	-1.18904
C	-4.27351	-2.23990	-1.73883
H	-5.13534	-2.86763	-1.45329
H	-3.35212	-2.76429	-1.43420
H	-4.26797	-2.17116	-2.83880
C	-2.92742	1.53820	3.23664
H	-2.20492	2.03423	2.56318
C	-4.05337	2.55322	3.56001
H	-4.80803	2.09571	4.22274
H	-4.56990	2.90713	2.65385
H	-3.63699	3.43415	4.07771
C	-2.18043	1.16871	4.53569
H	-1.68035	2.06215	4.94599
H	-1.42034	0.38809	4.37025

H   -2.87002 0.80181 5.31533
C   1.63681 -1.26778 0.12994
C   2.91130 -3.18729 -0.07635
H   3.14050 -4.19055 -0.42275
C   3.61444 -2.31881 0.70035
H   4.58485 -2.40713 1.17926
C   3.38115 -0.03482 1.57404
C   3.16941 0.00384 2.97847
C   3.79250 1.04008 3.70217
H   3.64922 1.09631 4.78513
C   4.60690 1.98349 3.06603
H   5.08586 2.77595 3.65033
C   4.83027 1.89779 1.68710
H   5.49468 2.61991 1.20368
C   4.24001 0.88165 0.90797
C   4.62238 0.75009 -0.56762
H   3.84974 0.14270 -1.06978
C   4.69676 2.10797 -1.29807
H   3.77765 2.69975 -1.16599
H   4.83907 1.94116 -2.37851
H   5.55285 2.71149 -0.94949
C   5.97548 0.00691 -0.70569
H   6.78147 0.56662 -0.20037
H   6.24899 -0.09415 -1.76985
H   5.94294 -1.00421 -0.26801
C   2.38139 -1.07094 3.72836
H   1.70306 -1.56498 3.00724
C   1.51072 -0.49807 4.86492
H   2.12371 -0.13084 5.70589
H   0.84678 -1.28321 5.26253
H   0.87970 0.33635 4.51674
C   3.33346 -2.15470 4.29324
H   3.91642 -2.65007 3.50010
H   2.75749 -2.93122 4.82505
H   4.04681 -1.71000 5.00854
C   0.79925 -3.20259 -1.33204
C   -0.18830 -4.07474 -0.79787
C   -0.99054 -4.78440 -1.71330
H   -1.75884 -5.46553 -1.33502
C   -0.80075 -4.66057 -3.09583
H   -1.43132 -5.22890 -3.78680
C   0.20418 -3.82485 -3.59415
H   0.35614 -3.75017 -4.67478
C   1.03472 -3.08026 -2.73116
C   2.18685 -2.25460 -3.30935
H   2.53261 -1.55616 -2.52712
C   3.37445 -3.17745 -3.68040
H   3.74633 -3.74345 -2.81059
H   4.21202 -2.58091 -4.07996
H   3.07742 -3.90576 -4.45471
C   1.77041 -1.41082 -4.53348
H   1.52561 -2.04308 -5.40415
H   2.60055 -0.74963 -4.83169
H   0.89154 -0.77980 -4.32074
C   -0.32876 -4.33408 0.70239
H   0.17301 -3.50497 1.23927
C   -1.79474 -4.38645 1.17909
H   -2.32763 -5.26082 0.76759
H   -2.36177 -3.48502 0.88719
H   -1.83106 -4.46976 2.27786
C   0.40556 -5.63969 1.09726
H   0.32751 -5.81036 2.18440
H   1.47544 -5.60631 0.83442
H   -0.04092 -6.50795 0.58256
C   1.08095 0.98462 -1.41502

(iv) ZnH/ZnMe Exchange

9
$ZnMe_2$
SCF = -307.055862891
H(0 K)= -306.986823
G(298 K)= -307.017003
SCF(CORR) = -1859.52284229
Low Freq. = 13.9643cm-1, 141.6280cm-1

Zn 0.00000 -0.00009 0.00000
C   1.94153 -0.02001 0.00000
C   -1.94154 0.02034 -0.00000
H   2.33389 -0.53925 0.89169
H   2.33389 -0.53925 -0.89169
H   2.35028 1.00538 0.00000
H   -2.35056 -1.00500 -0.00000
H   -2.33373 0.53942 0.89182
H   -2.33373 0.53942 -0.89182

6
ZnHMe
SCF = -267.732061735
H(0 K)= -267.691448
G(298 K)= -267.716455
SCF(CORR) = -1820.18581015
Low Freq. = 435.2742cm-1, 435.3444cm-1

Zn 0.00028 -0.42758 0.00000
H   -0.00963 -1.96362 0.00000
C   0.00028 1.51141 -0.00000
H   -1.03043 1.90672 -0.00000
H   0.51502 1.90790 0.89236
H   0.51502 1.90790 -0.89236

TS(5^{+}-5^{+}_{ZnMe})1 (+23.0 kcal/mol)
149
SCF = -3292.88474838
H(0 K)= -3291.656168
G(298 K)= -3291.772548
SCF(CORR) = -7951.50899862
Low Freq. = -55.8966cm-1, 10.3551cm-1

Ru 0.16407 0.08293 0.13293
H   0.85900 1.59921 -0.10769

H	-0.04209	-1.53263	0.54517
H	0.13854	-0.35203	-1.53459
Zn	-0.85754	-2.26719	-1.10445
H	-0.41467	-2.69129	-2.51868
Zn	0.67310	1.46445	-1.90610
H	0.73957	2.29907	-3.18627
O	0.45814	0.91497	3.06670
N	-2.85847	0.76477	-1.03893
N	-2.12608	2.34881	0.26189
N	3.35752	-0.04020	-0.06823
N	2.62636	-1.89062	0.80244
C	-1.78299	1.06932	-0.18740
C	-3.76335	1.82427	-1.13116
H	-4.63619	1.76846	-1.77313
C	-3.31320	2.80563	-0.30962
H	-3.71569	3.78501	-0.07147
C	-1.45863	3.26491	1.18362
C	-1.75056	3.17208	2.57253
C	-1.20335	4.16087	3.41316
H	-1.39467	4.11071	4.48815
C	-0.44448	5.21939	2.90097
H	-0.04163	5.98059	3.57684
C	-0.22727	5.32009	1.52335
H	0.33231	6.17227	1.12586
C	-0.73627	4.35615	0.62962
C	-0.57393	4.58318	-0.87514
H	-0.84601	3.64747	-1.40029
C	-1.53415	5.69237	-1.37271
H	-2.59106	5.45828	-1.16667
H	-1.42479	5.83199	-2.46170
H	-1.30418	6.65415	-0.88259
C	0.87313	4.93513	-1.27415
H	1.17493	5.92031	-0.87851
H	0.96197	4.97888	-2.37223
H	1.59642	4.18987	-0.90612
C	-2.70520	2.12414	3.15258
H	-2.62283	1.20834	2.53939
C	-4.17062	2.62531	3.06788
H	-4.29335	3.55919	3.64331
H	-4.85509	1.87282	3.49607
H	-4.49067	2.82025	2.03189
C	-2.38826	1.74100	4.61319
H	-1.33394	1.45301	4.74529
H	-3.02016	0.89013	4.91653
H	-2.61170	2.56773	5.30973
C	-3.23947	-0.45831	-1.74407
C	-4.27641	-1.26692	-1.17884
C	-4.74299	-2.35052	-1.94869
H	-5.52929	-2.99161	-1.54016
C	-4.24589	-2.61035	-3.23155
H	-4.62757	-3.46133	-3.80470
C	-3.29373	-1.75722	-3.79445
H	-2.94701	-1.93379	-4.81631
C	-2.79383	-0.64653	-3.08434
C	-1.93409	0.37537	-3.82313
H	-1.44501	1.02145	-3.07090
C	-2.83360	1.29455	-4.68745
H	-3.35843	0.71086	-5.46335
H	-2.21892	2.05878	-5.19281
H	-3.59524	1.81574	-4.08409
C	-0.82986	-0.26032	-4.68945
H	-1.25091	-0.81066	-5.54837
H	-0.21295	-0.96423	-4.10672
H	-0.18015	0.53034	-5.09941
C	-4.98064	-0.95677	0.14877
H	-4.32040	-0.30521	0.75069
C	-6.30978	-0.19699	-0.10605
H	-6.99184	-0.81086	-0.71913
H	-6.16317	0.76279	-0.62340
H	-6.81347	0.01345	0.85258
C	-5.30241	-2.21697	0.98381
H	-5.69878	-1.91774	1.96816
H	-4.41897	-2.85031	1.15315
H	-6.07918	-2.83502	0.50231
C	2.17009	-0.69168	0.24661
C	4.47922	-0.77985	0.31019
H	5.48605	-0.40564	0.15395
C	4.02037	-1.93511	0.85667
H	4.54443	-2.78507	1.28058
C	1.89524	-3.06698	1.26041
C	1.75999	-4.17124	0.36813
C	1.11998	-5.32462	0.86812
H	0.99598	-6.19013	0.21117
C	0.65861	-5.39319	2.18772
H	0.16020	-6.29956	2.54648
C	0.87547	-4.32139	3.06168
H	0.56592	-4.40716	4.10716
C	1.52824	-3.14703	2.63295
C	1.95242	-2.09664	3.66217
H	2.10279	-1.13914	3.13586
C	0.91699	-1.86584	4.78083
H	-0.08551	-1.65901	4.37806
H	1.22281	-1.00535	5.39808
H	0.84327	-2.73650	5.45531
C	3.30785	-2.50426	4.29749
H	3.21579	-3.47077	4.82232
H	3.62383	-1.74609	5.03420
H	4.11163	-2.60186	3.55031
C	2.38139	-4.20838	-1.03524
H	2.46684	-3.16864	-1.40195
C	1.55512	-5.00807	-2.06608
H	1.55809	-6.08880	-1.84206
H	2.00487	-4.88865	-3.06611
H	0.51331	-4.65863	-2.12865
C	3.80524	-4.82356	-0.97453
H	4.49242	-4.25469	-0.33037
H	4.24530	-4.85782	-1.98565
H	3.75973	-5.85734	-0.59073
C	3.58960	1.22538	-0.74322
C	3.77233	1.21166	-2.15580
C	4.15280	2.42652	-2.76437
H	4.29353	2.45848	-3.84838
C	4.37942	3.58031	-2.00574
H	4.68753	4.50636	-2.50156

```
C   4.23391  3.54870 -0.61247
H   4.44256  4.44989 -0.02756
C   3.84296  2.37216  0.05559
C   3.79277  2.33693  1.58354
H   3.29168  1.40102  1.88590
C   5.22608  2.31757  2.16989
H   5.81151  1.45623  1.80727
H   5.18773  2.26165  3.27104
H   5.77604  3.23483  1.89703
C   2.98331  3.50553  2.18109
H   3.46427  4.47932  1.98344
H   2.91385  3.39176  3.27579
H   1.95924  3.54223  1.77825
C   3.69930 -0.07376 -2.98945
H   3.06277 -0.79765 -2.44730
C   3.08684  0.13420 -4.39094
H   3.75553  0.71920 -5.04582
H   2.11730  0.65518 -4.34571
H   2.93333 -0.84307 -4.87805
C   5.11097 -0.69791 -3.13570
H   5.05770 -1.62278 -3.73492
H   5.56029 -0.95186 -2.16274
H   5.79037  0.00196 -3.65218
C   0.27920  0.57705  1.95977
Zn -1.68079 -1.88399  1.29794
C  -2.53517 -1.60915  3.03919
H  -1.94471 -0.94366  3.68569
H  -3.53882 -1.16942  2.92304
H  -2.65001 -2.58255  3.54692
C  -2.00519 -3.69986  0.04421
H  -2.35195 -4.02149  1.04516
H  -2.87133 -3.78068 -0.63010
H  -1.25286 -4.45704 -0.23739
```

Int(5$^+$-5$^+_{ZnMe}$)1 (+0.7 kcal/mol)

```
143
SCF  =          -3025.18844336
H(0 K)=         -3024.001326
G(298 K)=       -3024.113038
SCF(CORR) =     -6131.33764696
Low Freq. = 12.6633cm-1,
22.4218cm-1
```

```
C  -1.22464  3.97304 -0.93507
C  -1.81262  3.24900  0.13959
C  -1.87304  3.75586  1.46836
C  -1.25965  5.00333  1.71101
C  -0.63014  5.71367  0.68413
C  -0.61738  5.20469 -0.62260
N  -2.53622  2.02666 -0.15492
C  -2.05050  0.72752 -0.23019
N  -3.20632 -0.01420 -0.44633
C  -4.34364  0.79684 -0.49571
C  -3.92287  2.07795 -0.31733
C  -3.37102 -1.45572 -0.51608
C  -3.52633 -2.16790  0.70703
C  -3.80760 -3.54555  0.61974
C  -3.97152 -4.17730 -0.62078
C  -3.85528 -3.44244 -1.80558
C  -3.55603 -2.06370 -1.78856
Ru -0.03242  0.06091 -0.13272
Zn -0.44803 -2.39976 -0.67769
C  -0.37083 -4.11800 -1.58283
C  -3.51993 -1.46825  2.06773
C  -4.97714 -1.21758  2.53184
C  -3.53674 -1.27338 -3.09952
C  -4.98266 -0.99888 -3.58430
C  -2.64150  3.05612  2.59294
C  -3.90149  3.87223  2.97140
C  -1.34800  3.50836 -2.38657
C  -0.05586  3.68597 -3.20764
Zn  0.75365  2.15442  1.06975
C   1.98553 -0.59598  0.21070
N   2.53361 -1.21760  1.33514
C   3.88466 -1.51900  1.14643
C   4.22123 -1.08437 -0.09768
N   3.07177 -0.52457 -0.65708
C   1.92484 -1.52713  2.62432
C   1.38511 -2.82443  2.83290
C   0.87507 -3.12020  4.11325
C   0.91974 -2.18193  5.14942
C   1.51315 -0.93376  4.93398
C   2.04874 -0.58093  3.67906
C   3.15775  0.09724 -1.97172
C   3.02526 -0.71942 -3.12544
C   3.20425 -0.10154 -4.37896
C   3.51846  1.25751 -4.48233
C   3.68617  2.02846 -3.32608
C   3.52685  1.46800 -2.04346
C   2.77594 -2.22801 -3.05073
C   1.84079 -2.74614 -4.16443
C   3.86340  2.30235 -0.80497
C   3.37527  3.76267 -0.89175
C   1.44276 -3.92938  1.77572
C   0.19436 -4.83599  1.76791
C   2.82801  0.72996  3.54068
C   2.24074  1.88756  4.37362
C  -0.08097 -0.12888 -2.01150
O  -0.19021 -0.06285 -3.18033
C   5.39072  2.27833 -0.54252
C   4.11601 -3.00505 -3.10128
C   4.31203  0.52527  3.94191
C   2.70945 -4.80080  1.97241
C  -2.73622 -1.97594 -4.21714
C  -2.73534 -2.23392  3.15089
C  -2.52832  4.24553 -3.06908
C  -1.76918  2.79650  3.83910
H   0.51363  1.57732 -0.62545
H  -0.63626 -1.31129  0.68553
H  -0.07724  0.58314  1.51185
H   1.47387  3.31582  1.74453
H   4.46992 -2.01034  1.91720
H   5.16224 -1.11098 -0.63823
H   3.10156 -0.69939 -5.28883
```

H   3.65306 1.71405 -5.46823
H   3.96843 3.08128 -3.41818
H   3.36729 1.83790 0.06866
H   5.77141 1.25445 -0.39742
H   5.63473 2.86228 0.36135
H   5.93614 2.72311 -1.39276
H   3.91448 4.32901 -1.67049
H   3.55527 4.27419 0.06803
H   2.29631 3.82680 -1.10773
H   2.29537 -2.44627 -2.07814
H   4.64657 -2.79824 -4.04674
H   3.93207 -4.09177 -3.04630
H   4.78899 -2.73512 -2.27179
H   0.90231 -2.17204 -4.21294
H   1.59482 -3.80509 -3.98193
H   2.32204 -2.69562 -5.15619
H   0.45230 -4.11043 4.30475
H   0.51750 -2.43478 6.13571
H   1.58439 -0.22373 5.76255
H   2.80708 1.03520 2.47629
H   4.38350 0.19249 4.99182
H   4.86636 1.47457 3.84759
H   4.81925 -0.22359 3.31426
H   2.40091 1.73199 5.45448
H   1.16045 2.01857 4.20420
H   2.73882 2.83262 4.10267
H   1.52293 -3.44996 0.78045
H   2.69156 -5.28952 2.96183
H   3.63654 -4.20970 1.90556
H   2.75599 -5.59048 1.20311
H   0.24890 -5.54264 0.92440
H -0.74110 -4.25954 1.66993
H   0.12345 -5.43853 2.68937
H -4.46678 3.01711 -0.28205
H -5.33460 0.37947 -0.64541
H -3.93505 -4.12402 1.53985
H -4.20833 -5.24542 -0.66204
H -4.00870 -3.94233 -2.76651
H -3.05373 -0.29976 -2.90456
H -1.70796 -2.20999 -3.90024
H -2.67574 -1.31981 -5.10120
H -3.21911 -2.91418 -4.53995
H -5.51335 -1.94470 -3.78907
H -4.96702 -0.41051 -4.51730
H -5.57337 -0.43796 -2.84168
H -3.02803 -0.48781 1.94429
H -3.18650 -3.21586 3.37578
H -2.73717 -1.65568 4.09014
H -1.68488 -2.39164 2.85910
H -5.54475 -0.61709 1.80170
H -4.98580 -0.67942 3.49502
H -5.51397 -2.17162 2.67252
H -1.28576 5.42296 2.72149
H -0.15878 6.67824 0.89739
H -0.15257 5.78854 -1.42278
H -1.58556 2.43091 -2.37818
H -3.47960 4.08343 -2.53504
H -2.65481 3.88725 -4.10482
H -2.34432 5.33322 -3.10566
H   0.22557 4.74794 -3.31403
H -0.20528 3.28642 -4.22439
H   0.79259 3.14547 -2.75806
H -2.97909 2.07355 2.21982
H -1.37968 3.73311 4.27285
H -0.90865 2.15070 3.59680
H -2.36248 2.29010 4.61921
H -4.48280 3.34318 3.74533
H -4.56108 4.03629 2.10292
H -3.62827 4.86267 3.37395
H -0.58861 -3.99322 -2.65503
H   0.62894 -4.57059 -1.47679
H -1.12017 -4.79881 -1.15048

TS(5^+-5^+_{ZnMe})1 (+12.7 kcal/mol)
143
SCF =   -3025.16835952
H(0 K)=   -3023.981932
G(298 K)=   -3024.094264
SCF(CORR) =   -6131.31717438
Low Freq. = -88.0749cm-1,
11.9089cm-1

C   0.82881 -4.15229 0.34791
C -0.14081 -3.57742 -0.51578
C -0.18586 -3.84734 -1.91335
C   0.82764 -4.67609 -2.44036
C   1.80988 -5.23642 -1.61658
C   1.80441 -4.98074 -0.23867
N -1.25981 -2.87231 0.08652
C -1.46100 -1.51151 0.32218
N -2.74543 -1.50204 0.86036
C -3.28763 -2.78620 0.96759
C -2.35333 -3.64432 0.48355
C -3.64942 -0.39987 1.13647
C -4.53408 -0.00392 0.08608
C -5.60882 0.83911 0.42686
C -5.81735 1.25384 1.74735
C -4.93984 0.84874 2.76017
C -3.84818 0.00034 2.49023
Ru 0.01630 0.04591 0.12100
C   0.26732 -0.03786 1.98803
O   0.39175 -0.22515 3.14076
C -4.39837 -0.50989 -1.35159
C -5.56712 -1.45889 -1.70934
C -2.98486 -0.51217 3.64131
C -3.76011 -1.55363 4.48505
C -1.34069 -3.38784 -2.81182
C -2.39684 -4.51455 -2.94360
C   0.79150 -3.96394 1.86441
C   2.15502 -3.53066 2.43977
Zn -2.12116 1.76111 0.44453
Zn 1.11355 -1.04218 -1.84620
C   1.70583 1.32407 -0.30307
N   1.72285 2.42405 -1.16057

C	3.01241	2.92613	-1.33284
C	3.83815	2.16222	-0.56808
N	3.04431	1.19959	0.05998
C	0.60057	3.21191	-1.64886
C	0.19413	4.32178	-0.85844
C	-0.84389	5.12887	-1.36499
C	-1.43973	4.85513	-2.60171
C	-0.98388	3.78190	-3.37783
C	0.05895	2.94577	-2.93137
C	3.68046	0.31359	1.02891
C	3.65124	0.67897	2.40470
C	4.36764	-0.13793	3.30045
C	5.11984	-1.23122	2.85281
C	5.18136	-1.52569	1.48759
C	4.47159	-0.75905	0.54071
C	2.99165	1.96781	2.90883
C	2.51780	1.89640	4.37557
C	4.65615	-1.05592	-0.94873
C	4.47317	-2.54932	-1.28558
C	0.89358	4.72054	0.44504
C	-0.07674	4.89066	1.63322
C	0.63735	1.88070	-3.86056
C	-0.44321	1.06498	-4.59792
C	6.04476	-0.57311	-1.43631
C	3.96896	3.16274	2.75450
C	1.61299	2.52711	-4.87504
C	1.71646	6.01500	0.23265
C	-2.47103	0.63540	4.53848
C	-4.30259	0.64801	-2.37016
C	0.29206	-5.25484	2.55818
C	-0.88775	-2.94111	-4.21864
H	1.11910	-1.22293	0.02980
H	-0.51884	1.61914	-0.00141
H	-0.36316	0.00890	-1.56846
H	3.20747	3.78759	-1.96449
H	4.90944	2.20875	-0.39923
H	4.35531	0.10289	4.36668
H	5.67768	-1.84077	3.57101
H	5.80138	-2.35835	1.14142
H	3.88853	-0.49599	-1.51407
H	6.19842	0.50540	-1.26704
H	6.15849	-0.76524	-2.51680
H	6.85271	-1.10963	-0.90940
H	5.27418	-3.16756	-0.84421
H	4.50713	-2.69534	-2.37817
H	3.50752	-2.93588	-0.92334
H	2.10569	2.16711	2.27813
H	4.88400	2.98934	3.34684
H	3.49858	4.09002	3.12492
H	4.26832	3.33195	1.70806
H	1.89060	1.01346	4.56712
H	1.92768	2.79683	4.61562
H	3.36972	1.87975	5.07740
H	-1.17766	5.99552	-0.78524
H	-2.24482	5.49639	-2.97458
H	-1.42659	3.60464	-4.36265
H	1.22649	1.17503	-3.24549

H	1.08527	3.25083	-5.52010
H	2.06096	1.75466	-5.52314
H	2.43352	3.06409	-4.37056
H	-1.00990	1.68518	-5.31355
H	-1.16159	0.61556	-3.89216
H	0.02841	0.25345	-5.17690
H	1.60133	3.91914	0.71782
H	1.06054	6.86485	-0.02325
H	2.45144	5.90442	-0.58194
H	2.26492	6.27763	1.15316
H	0.48704	5.15558	2.54388
H	-0.63854	3.96415	1.83681
H	-0.80897	5.69710	1.45626
H	-2.35059	-4.72543	0.38476
H	-4.28443	-2.95228	1.36477
H	-6.30642	1.15589	-0.35490
H	-6.67116	1.89480	1.98923
H	-5.12069	1.17116	3.79021
H	-2.10560	-1.01823	3.20956
H	-1.93268	1.40014	3.95467
H	-1.77603	0.23724	5.29560
H	-3.29507	1.13532	5.07595
H	-4.65936	-1.10576	4.94217
H	-3.12109	-1.93435	5.29957
H	-4.08445	-2.41586	3.87879
H	-3.46061	-1.08774	-1.42373
H	-5.21494	1.26877	-2.37295
H	-4.17587	0.24413	-3.38893
H	-3.44556	1.31063	-2.15938
H	-5.61974	-2.31791	-1.02025
H	-5.44260	-1.85024	-2.73318
H	-6.53624	-0.93272	-1.66742
H	0.83019	-4.90062	-3.51076
H	2.57688	-5.88722	-2.04828
H	2.56295	-5.44488	0.39939
H	0.07054	-3.15920	2.08900
H	-0.70946	-5.55366	2.20568
H	0.23668	-5.10508	3.64982
H	0.97903	-6.09754	2.36767
H	2.92088	-4.31538	2.31286
H	2.06089	-3.33668	3.52126
H	2.52501	-2.61180	1.95898
H	-1.82664	-2.52238	-2.32428
H	-0.51485	-3.79025	-4.81677
H	-0.08765	-2.18398	-4.17715
H	-1.74340	-2.51271	-4.76684
H	-3.23123	-4.18395	-3.58543
H	-2.81697	-4.80934	-1.96884
H	-1.94941	-5.41154	-3.40538
H	1.99130	-1.43506	-3.03554
C	-3.33210	3.29266	0.60465
H	-3.87193	3.28844	1.56333
H	-2.73815	4.21519	0.52589
H	-4.06805	3.27702	-0.21443

$\mathbf{5^{+}_{ZnMe}}$ (+2.4 kcal/mol)
143

SCF = -3025.18408214
H(0 K)= -3023.997739
G(298 K)= -3024.109764
SCF(CORR) = -6131.33385978
Low Freq. = 16.4234cm-1,
20.1888cm-1

C	-0.02242	-4.39105	0.01117
C	-0.95506	-3.49383	-0.56857
C	-1.25313	-3.48593	-1.96213
C	-0.52097	-4.37063	-2.78095
C	0.42657	-5.24882	-2.23930
C	0.66007	-5.26542	-0.85953
N	-1.75184	-2.65443	0.30918
C	-1.66837	-1.28037	0.51753
N	-2.78336	-1.03020	1.31815
C	-3.47782	-2.20406	1.61882
C	-2.83709	-3.22055	0.98159
C	-3.42963	0.24085	1.62194
C	-4.53950	0.61482	0.81068
C	-5.21796	1.80208	1.15296
C	-4.82267	2.57760	2.24885
C	-3.76446	2.15273	3.06162
C	-3.06341	0.96111	2.78850
Ru	0.07012	-0.05021	0.13037
C	0.63667	-0.64241	1.83299
O	0.90092	-1.11171	2.87247
C	-5.08778	-0.25447	-0.32750
C	-6.43920	-0.88469	0.09293
C	-2.05876	0.42097	3.80266
C	-2.81229	-0.17521	5.01814
C	-2.41191	-2.67176	-2.55065
C	-3.72005	-3.50133	-2.48419
C	0.21756	-4.48228	1.51876
C	1.71539	-4.40777	1.88214
Zn	-1.54520	1.84275	-0.51988
Zn	0.73569	-1.06322	-2.07040
C	1.84318	1.08659	-0.24028
N	1.98806	2.29564	-0.91698
C	3.32887	2.67402	-1.03312
C	4.06299	1.71109	-0.41675
N	3.16413	0.75886	0.06750
C	0.99915	3.25204	-1.38859
C	0.64569	4.31958	-0.51482
C	-0.08094	5.39384	-1.06490
C	-0.43851	5.41351	-2.41837
C	-0.09264	4.34325	-3.25153
C	0.64598	3.24610	-2.76573
C	3.70627	-0.35205	0.83887
C	3.92234	-0.16365	2.23172
C	4.57018	-1.20161	2.92922
C	5.01392	-2.35546	2.27273
C	4.82698	-2.49320	0.89266
C	4.17924	-1.49323	0.14009
C	3.56770	1.13715	2.95962
C	3.06026	0.92044	4.40172
C	4.10056	-1.62363	-1.38141
C	3.65587	-3.02543	-1.84515
C	1.08058	4.37440	0.95064
C	-0.10646	4.65560	1.89815
C	1.10350	2.15481	-3.73198
C	-0.05822	1.60049	-4.58367
C	5.45592	-1.25353	-2.03313
C	4.79088	2.08943	2.98798
C	2.24863	2.66479	-4.63974
C	2.19783	5.42607	1.15362
C	-1.03229	1.47434	4.26464
C	-5.25202	0.50381	-1.66207
C	-0.41016	-5.77558	2.09401
C	-2.17340	-2.19529	-3.99860
H	0.85550	-1.47966	-0.34805
H	-0.60349	1.31041	0.90312
H	-0.52840	0.37117	-1.44652
H	3.61782	3.59275	-1.53458
H	5.13351	1.60499	-0.27127
H	4.74423	-1.09450	4.00362
H	5.52407	-3.14284	2.83688
H	5.20601	-3.38431	0.38334
H	3.35129	-0.89546	-1.74920
H	5.77037	-0.22737	-1.78183
H	5.38666	-1.32794	-3.13180
H	6.25058	-1.94067	-1.69463
H	4.41971	-3.78964	-1.62005
H	3.50028	-3.02972	-2.93674
H	2.71448	-3.34105	-1.36497
H	2.76064	1.63499	2.39068
H	5.63273	1.61974	3.52541
H	4.53517	3.02590	3.51270
H	5.14042	2.35448	1.97762
H	2.23774	0.19107	4.44468
H	2.70012	1.87777	4.81466
H	3.86642	0.57003	5.06914
H	-0.35379	6.23727	-0.42287
H	-0.99029	6.26671	-2.82574
H	-0.37325	4.37115	-4.30896
H	1.50663	1.31638	-3.13396
H	1.91035	3.50894	-5.26518
H	2.59096	1.86016	-5.31234
H	3.11593	3.00796	-4.05162
H	-0.45927	2.36170	-5.27447
H	-0.89134	1.24689	-3.95271
H	0.29378	0.75269	-5.19442
H	1.49163	3.38587	1.22145
H	1.84101	6.43788	0.89457
H	3.08093	5.21021	0.52991
H	2.52234	5.44341	2.20790
H	0.23234	4.62215	2.94747
H	-0.91233	3.91220	1.77548
H	-0.54099	5.65530	1.72670
H	-3.04546	-4.28504	0.93260
H	-4.37018	-2.19242	2.23731
H	-6.07839	2.11508	0.55278
H	-5.36169	3.49959	2.48941
H	-3.50015	2.73448	3.95013

H	-1.50530	-0.40224	3.32498
H	-0.48567	1.90500	3.40984
H	-0.29845	1.01113	4.94472
H	-1.51222	2.30121	4.81608
H	-3.39082	0.60195	5.54687
H	-2.09670	-0.61321	5.73471
H	-3.51681	-0.96750	4.71362
H	-4.37835	-1.08026	-0.50848
H	-5.96403	1.34230	-1.57536
H	-5.64685	-0.18018	-2.43269
H	-4.29479	0.91302	-2.02017
H	-6.35369	-1.46755	1.02496
H	-6.81062	-1.55870	-0.69753
H	-7.20356	-0.10606	0.25799
H	-0.71452	-4.38590	-3.85702
H	0.97252	-5.93393	-2.89561
H	1.38238	-5.97440	-0.44283
H	-0.28078	-3.62195	1.99775
H	-1.49307	-5.83934	1.89459
H	-0.26399	-5.81825	3.18659
H	0.06079	-6.67227	1.65527
H	2.26925	-5.28592	1.50696
H	1.83499	-4.38820	2.97820
H	2.19224	-3.50392	1.47230
H	-2.54982	-1.77195	-1.92368
H	-2.17118	-3.03608	-4.71360
H	-1.21712	-1.65701	-4.11015
H	-2.98788	-1.51761	-4.30425
H	-4.56456	-2.91851	-2.89040
H	-3.97383	-3.79367	-1.45231
H	-3.62417	-4.42242	-3.08472
H	1.20285	-1.35047	-3.49798
C	-2.84658	3.09095	-1.24465
H	-3.79065	3.01989	-0.68604
H	-2.45261	4.11432	-1.15884
H	-3.03832	2.88249	-2.30990

S3. References

1. Ritter, F.; Spaniol, T. P.; Douair, I.; Maron, L.; Okuda, J. Molecular zinc hydride cation $[ZnH]^+$: Synthesis, structure, and CO_2 hydrosilylation catalysis. *Angew. Chem. Int. Ed.* **2020,** *59*, 23335-233342.

2. The smaller resonances at δ -8.8 and -10.6 ($^{31}P\{^1H\}$ signals at δ 58 and 52) are assigned to *mer*- and *fac*-$[Ru(PPh_3)_3(ZnMe)H_3]$. The hydride multiplet at δ -11.7 and $^{31}P\{^1H\}$ signal near to δ 50 are characteristic of $[Ru(PPh_3)_3H_3]^-$; we thus tentatively assign the product as $[Ru(PPh_3)_3H_3Zn(THF][BAr^F{}_4]$. See; Miloserdov, F. M.; Rajabi, N. A.; Lowe, J. P.; Mahon, M. F.; Macgregor, S. A.; Whittlesey, M. K. Zn-Promoted C–H reductive elimination and H_2 activation via a dual unsaturated heterobimetallic Ru–Zn intermediate. *J. Am. Chem. Soc.* **2020**, *142*, 6340-6349).

3. Kocen, A. L.; Klimovica, K.; Broolhart, M.; Daugulis, O. Alkene isomerization by ″sandwich″ by diimine-palladium catalysts. *Organometallics* **2017**, *36*, 787-790.

4. Siedle, A. R.; Newmark, R. A.; Pignolet, L. H.; Wang, D. X.; Albright, T. A. Organometallic chemistry of fluorocarbon acids. Synthesis and structural and dynamic properties of $(\pi\text{-arene})RuH(PPh_3)_2^+$ derivatives. *Organometallics* **1986**, *5*, 38-47.

5. Walsh, A. M.; Sotorrios, L.; Cameron, R. G.; Pécharman, A.-F.; Procacci, B.; Lowe, J. P.; Macgregor, S. A.; Mahon, M. F.; Hunt, N. T.; Whittlesey, M. K. Isolobal cationic iridium dihydride and dizinc complexes: A dual role for the ZnR ligand enhances H_2 activation. *Inorg. Chem.* **2024**, *63*, 22944-22954
